# The Complete Chloroplast Genomes of Nine Smilacaceae Species from Hong Kong: Inferring Infra- and Inter-Familial Phylogeny

**DOI:** 10.3390/ijms24087460

**Published:** 2023-04-18

**Authors:** Kwan-Ho Wong, Tin-Yan Siu, Stacey Shun-Kei Tsang, Bobby Lim-Ho Kong, Hoi-Yan Wu, Grace Wing-Chiu But, Jerome Ho-Lam Hui, Pang-Chui Shaw, David Tai-Wai Lau

**Affiliations:** 1 Shiu-Ying Hu Herbarium, School of Life Sciences, The Chinese University of Hong Kong, Shatin, Hong Kong, China; kwanhowong@cuhk.edu.hk (K.-H.W.); joycesiuty@hotmail.com (T.-Y.S.); stacey.tsang@link.cuhk.edu.hk (S.S.-K.T.); 2 School of Life Sciences, The Chinese University of Hong Kong, Shatin, Hong Kong, Chinajeromehui@cuhk.edu.hk (J.H.-L.H.); 3 Li Dak Sum Yip Yio Chin R & D Centre for Chinese Medicine, The Chinese University of Hong Kong, Shatin, Hong Kong, China; 4 Research Area of Ecology and Biodiversity, School of Biological Sciences, The University of Hong Kong, Pokfulam, Hong Kong, China; 5 Simon F.S. Li Marine Science Laboratory and State Key Laboratory of Agrobiotechnology, The Chinese University of Hong Kong, Shatin, Hong Kong, China; 6 State Key Laboratory of Research on Bioactivities and Clinical Applications of Medicinal Plants (The Chinese University of Hong Kong) and Institute of Chinese Medicine, The Chinese University of Hong Kong, Shatin, Hong Kong, China

**Keywords:** Smilacaceae, *Smilax*, *Heterosmilax*, chloroplast genomes, phylogeny, monophyly, nomenclatural revision, generic delimitation, *Ripogonum*

## Abstract

The Smilacaceae is a cosmopolitan family consisting of 200–370 described species. The family includes two widely accepted genera, namely *Smilax* and *Heterosmilax*. Among them, the taxonomical status of *Heterosmilax* has been continuously challenged. Seven *Smilax* and two *Heterosmilax* species can be found in Hong Kong, with most of them having medicinal importance. This study aims to revisit the infra-familial and inter-familial relationships of the Smilacaceae using complete chloroplast genomes. The chloroplast genomes of the nine Smilacaceae species from Hong Kong were assembled and annotated, which had sizes of 157,885 bp to 159,007 bp; each of them was identically annotated for 132 genes, including 86 protein-coding genes, 38 transfer RNA genes, and 8 ribosomal RNA genes. The generic status of *Heterosmilax* was not supported because it was nested within the *Smilax* clade in the phylogenetic trees, echoing previous molecular and morphological studies. We suggest delimitating the genus *Heterosmilax* as a section under the genus *Smilax*. The results of phylogenomic analysis support the monophyly of Smilacaceae and the exclusion of *Ripogonum* from the family. This study contributes to the systematics and taxonomy of monocotyledons, authentication of medicinal Smilacaceae, and conservation of plant diversity.

## 1. Introduction

### 1.1. The Family Smilacaceae

Smilacaceae Vent. is a cosmopolitan family composed of around 200 [1,2] to 370 [3] species. The family is characterised by a few major features, including climbing habit, paired tendrils derived from the petiole, reticular venation on leaves, dioecy, and fruiting berries [4,5,6]. It was first named in 1799 by Étienne-Pierre Ventenat in *Tableau du Règne Végétal, Selon la Méthode de Jussieu* [7]. The species of the Smilacaceae had long been treated as members of the family Liliaceae, which can be seen in a number of classification systems, including those of Bentham and Hooker in 1880 [8], Engler in 1888 [9] and Baker in 1898 [10]. However, based on their distinctive features which are absent from other Liliaceae species, they were separated and placed in the family Smilacaceae by Hutchinson in 1959 [11]. The current Angiosperm Phylogeny Group (APG) IV system accepted the status of this family based on molecular evidence, placing them in the order Liliales Perleb [12]. 

### 1.2. The Argument on the Genus Heterosmilax 

The family consists of two genera, namely the large genus *Smilax* L. and the small genus *Heterosmilax* Kunth with 12 species [3]. The Asiatic genus *Heterosmilax* was first described by Carl Sigismund Kunth in 1850 in his publication *Enumeratio Plantarum Omnium Hucusque Cognitarum* [13] and was later accepted in the monographic treatment *Monographiæ Phanerogamarum* by Alphonse de Candolle in 1878 [14]. Since then, the genus has been accepted in a number of classification systems, such as those of Bentham and Hooker in 1880 [8], Hutchinson in 1934 [15], Cronquist in 1981 [4], Dahlgren et al. in 1985 [16], and Takhtajan in 1997 [3]. However, the delimitation of this small genus is highly controversial. The only remarkable feature that distinguishes *Heterosmilax* from *Smilax* is the connated perianth segments, contrary to the six free tepals in *Smilax* [14,17]. Intermediate characters between these two genera were observed, such as the partly united tepals in *Smilax corbularia* Kunth, making the status of *Heterosmilax* more dubious [5,6]. In the past two decades, taxonomists have attempted to annul this genus, leaving the family Smilacaceae monogeneric [18,19]. However, the taxonomical treatment of this questionable taxon is nomenclaturally variable among botanists. In 1998, Walter Stephen Judd included *Heterosmilax* as a section of *Smilax* in *The Smilacaceae in the Southeastern United States* [20]. In 2006, Chen et al. suggested treating *Heterosmilax* as a section of *Smilax* based on morphological [6] (5 *Heterosmilax* versus 72 *Smilax*) and palynological (7 *Heterosmilax* versus 116 *Smilax*) evidence [5]. Meanwhile, in 2007, Kong et al. [2] and Chen et al. [21] suggested treating *Heterosmilax* as a subgenus of *Smilax* based on karyotypic evidence (3 *Heterosmilax* versus 35 *Smilax*) and seed coat morphology (3 *Heterosmilax* versus 54 *Smilax*). With the availability of molecular data, Qi et al. suggested placing *Heterosmilax* as a section of *Smilax* in 2013 based on short-fragment barcode sequences (6 *Heterosmilax* versus 119 *Smilax*), including nuclear locus ITS and chloroplast loci *matK* and *rpl16* [22].

### 1.3. Adulteration of Medicinally Valuable Smilacaceae Species and How Chloroplast Genomes Would Help in Authentication 

The rhizomes of two *Smilax* species i.e., *Smilax china* L. and *Smilax glabra* Roxb., have been applied in traditional Chinese medicine (TCM) under the Latin names Smilacis Chinae Rhizoma and Smilacis Glabrae Rhizoma, respectively. These two TCM materia medica have been prescribed in the *Pharmacopoeia of the People’s Republic of China* (PPRC) [23]. Besides the official drugs listed in PPRC, species of the genus *Heterosmilax*, such as *Heterosmilax japonica* Kunth and *Heterosmilax gaudichaudiana* (Kunth) Maxim., have been regionally applied as herbal medicines in China [24] under the names of “Baitufuling (white Tufuling)” [24], “Tubixie” [25], and “Baibixie (white Bixie)” [26]. Sharing highly similar appearances, the rhizomes of Smilacaceae species are easily confused with one another, causing historical substitution and adulteration [24,26,27,28]. Smilacis Glabrae Rhizoma, or Tufuling, have been adulterated by a number of Smilacaceae species [24,27,29,30]. *Smilax lanceifolia* Roxb. var. *opaca* A. DC. [28,31], *H. japonica* [24,26,31,32,33], and *H. gaudichaudiana* [24,28] have been applied as “Baitufuling (white Tufuling)” or “Baituling”, while *S. china* has been applied as “Hongtufuling (red Tufuling)” or “Hongtuling” [28,33] and “Hongbixie (red Bixie)” [26]. Inaccurate collection and medical prescription are more likely to affect the efficacy of medical treatment.

As herbal drugs are administered in decoction pieces or even in powder forms, the morphological characteristics for accurate authentication may be lost during processing [34]. The molecular approach for TCM authentication should be investigated to overcome this obstacle. Meanwhile, these medicinally valuable species have been recorded in the flora of Hong Kong; molecular markers are needed as baseline data to support the prosecution of those illegally collection of these species, which is a measure of nature conservation. The use of complete chloroplast genomes in the species authentication of medicinal plants had been successfully applied in *Hedyotis* [35], “Bang-Poong” (Apiaceae species) [36], and *Dipsacus* and its adulterant *Phlomoides* [37]. Divergence hotspots can be identified from the alignment of chloroplast genomes; hence, unique primers for amplifying targeted regions with informative nucleotide sites can be designed for the studied taxa. Amplicons with differentiating power can be subsequently utilised for species authentication.

### 1.4. Chloroplast Genomes of Smilacaceae and Its Potential Application 

The first complete chloroplast genome (cpGenome) of the Smilacaceae reported by Liu et al. in 2012 [38] was from *S. china* (GenBank accession number: HM536959). Other cpGenomes of Smilacaceae species were later reported, including those of *Smilax glycophylla* Sm. (NC_049023 = MT261169) and *Smilax nipponica* Miq. (NC_049024 = MT261170) in 2020 [39], *Smilax microphylla* C. H. Wright (NC_056390 = MW423607) [40] and *S. glabra* (NC_058534 = MZ566572) [41] in 2021, and *Smilax scobinicaulis* C. H. Wright (OL693684) [42] in 2022. 

Recently, the chloroplast genome was applied as a biological marker of new species. In 2022, the new species *Smilax weniae* P. Li, Z.C. Qi & Yan Liu from the limestone areas between Guizhou and Guangxi Provinces was named [43]. Apart from the traditional practices of publishing a new species using morphological description, photo documentation, illustration, and specimen support, this protologue was reported with the complete chloroplast genome (NC_067604 = OL444944). 

Despite previous efforts to elucidate the cpGenomes, a comparative study on the cpGenomes of Smilacaceae has not yet been conducted. This study aims to revisit the relationship of Smilacaceae species in Hong Kong using cpGenomes and to further infer the infra-familial and inter-familial phylogeny of the Smilacaceae. The cpGenomes of the nine Smilacaceae species in Hong Kong were assembled, annotated, and comparatively analysed with those publicly available, with respect to the genome content, structure, and phylogenomics. The results could contribute to the systematics and taxonomy of monocotyledons, medicinal plant authentication, and nature conservation.

## 2. Results

### 2.1. Genome Size, Structure, and Order

The cpGenome sizes of Smilacacaeae species in Hong Kong ranged from 157,885 bp to 159,007 bp in size (Table 1). The sizes of the large single copies (LSCs) of the nine cpGenomes ranged from 85,241 bp to 85,640 bp, while the size of the small single copies (SSCs) varied from 18,352 bp to 18,577 bp. Both LSC and SSC were separated by a pair of inverted repeats (IRs), which ranged from 27,098 bp to 27,478 bp in size. All nine cpGenomes showed the quadripartite structure typical of angiosperms (Figure S1). 

The number and content of genes in the nine newly assembled cpGenomes were the same. Each of them had 132 genes, including 86 protein-coding genes (PCGs), 38 transfer RNA (tRNA) genes, and 8 ribosomal RNA (rRNA) genes (Table 2). Eighteen genes, including *atpF*, *petB*, *petD*, *ndhA*, *ndhB* (x2), *rpoC1*, *rpl16*, *rpl2* (x2), *rps12*, *rps16*, *trnA-UGC*, *trnG-UCC*, *trnI-GAU*, *trnK-UUU*, *trnL-UAA*, and *trnV-UAC*, contained one intron, while two genes, i.e., *clpP1* and *pafI*, contained two introns. Each cpGenome shared the trans-spliced gene *rps12* and two double-copied open-reading frames (ORFs), namely *ycf1* and *ycf2*. Of the 132 genes, 19 genes had two copies (Table 2). 

The contents of nucleotides were relatively constant. The GC content ranged from 37.03% (*S. glabra*) to 37.31% (*H. japonica*). 

### 2.2. Genome Content and Structural Analysis

The content and structure of the nine cpGenomes were integrated with those of five GenBank-available species—*S. glycophylla* (NC_049023), *Smilax riparia* A. DC. (NC_062359), *S. scobinicaulis* (OL693684), *S. microphylla* (NC_056390), and *S. nipponica* (NC_049024)—to conduct content and structural analyses.

#### 2.2.1. Simple Sequence Repeats (SSRs)

**Figure 1:**
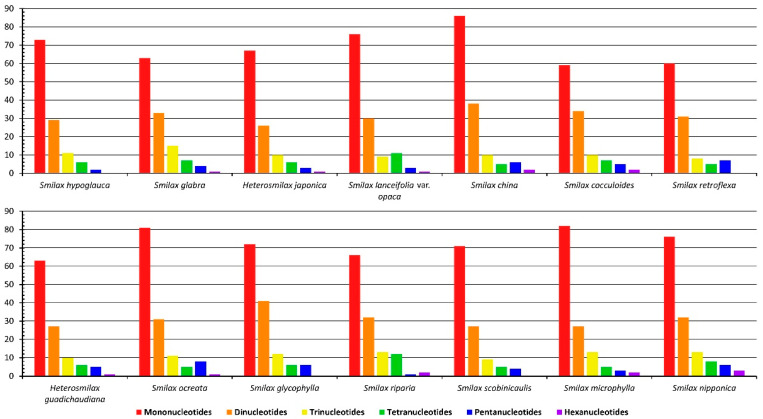
Types of SSRs detected from fourteen Smilacaceae species.

The total number of SSRs per cpGenome varied from 111 (*S. retroflexa*) to 147 (*S. china*). Mono-, di-, tri-, tetra-, and penta-nucleotide repeats were detected from all fourteen cpGenomes (Figure 1). The longer the SSRs were, the lower the abundances observed in each cpGenome. The mononucleotide repeat was the most abundant type of SSR in all studied genomes, ranging from 59 in *S. cocculoides* to 89 in *S. china*, followed by the dinucleotide repeat, ranging from 26 in *H. japonica* to 41 in *S. glycophylla*. The trinucleotide repeat was the third-most abundant type of SSR in most of the studied cpGenomes. Meanwhile, in the cpGenome of *S. lanceifolia* var. *opaca*, the tetranucleotide repeat (as 11) was the third-most abundant type of SSR instead of trinucleotide repeats (as 9). The pentanucleotide repeat was the commonly shared type of SSR with the least abundance in all cpGenomes, except in *S. china* (as 6), *S. retroflexa* (as 7), and *S. ocreata* (as 8), in which pentanucleotides repeats were the third-most abundant in these three accessions. The hexanucleotide repeat existed in ten cpGenomes other than those of *S. hypoglauca*, *S*. *retroflexa*, *S. glycophylla*, and *S. scobinicaulis*, with low abundance from 1 to 3.

**Figure 2:**
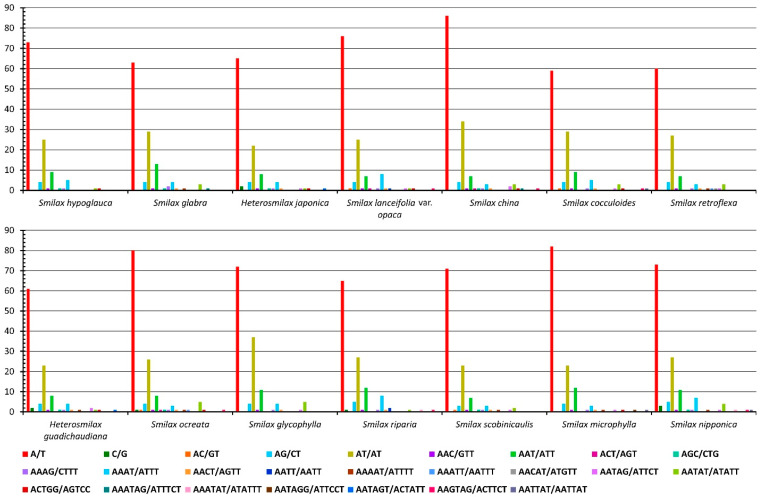
Complementarity of SSRs detected from fourteen Smilacaceae species.

Regarding the complementarity of SSRs, A/T repeats were the most abundant SSRs, ranging from 59 in *S. cocculoides* to 89 in *S. china* (Figure 2). AT/AT repeats were the second-most abundant SSR complementarity, ranging from 22 in *H. japonica* to 37 in *S. glycophylla*. AAT/ATT was the third-most abundant SSR complementarity, ranging from 7 (*S. lanceifolia* var. *opaca*, *S. china*, *S. retroflexa* and *S. scobinicaulis*) to 13 (*S. glabra*). It is noteworthy that the AACAT/ATGTT repeat was only detected in *S. retroflexa*, while the AATAGG/ATTCCT repeat was only detected in *S. microphylla*. These two species-specific SSRs could be utilised as potential molecular markers in species authentication.

#### 2.2.2. Long Tandem Repeats (LTRs)

**Figure 3:**
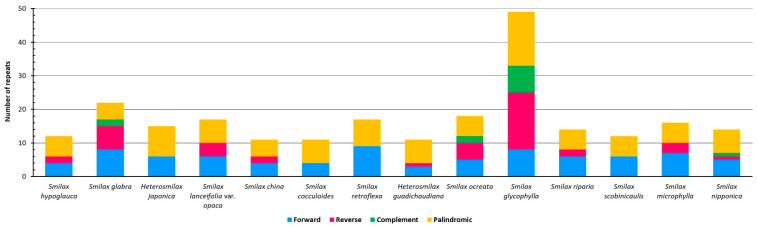
Types of LTRs detected from fourteen Smilacaceae species.

The total number of LTRs ranged from 11 (*S. china*, *S. cocculoides*, and *H. gaudichaudiana*) to 49 (*S. glycophylla*) (Figure 3). Only forward and palindromic repeats were commonly shared in the cpGenomes of all 14 species, with ranges of 3 to 9 and 5 to 16, respectively. Reverse repeats were identified in 10 cpGenomes other than those of *H. japonica*, *S. cocculoides*, *S. retroflexa*, and *S. scobinicaulis*, with numbers ranging from 1 to 17. Complement repeats were only identified in four cpGenomes, i.e., those of *S. glabra*, *S. ocreata*, *S. glycophylla,* and *S. nipponica*, with numbers ranging from 1 to 8. The cpGenome of *S. glycophylla* had the highest number of LTRs in total, including 8 forward repeats, 17 reverse repeats, 8 complement repeats, and 16 palindromic repeats, which was distinctively higher than that of other cpGenomes, which had less than 23 LTRs. 

**Figure 4:**
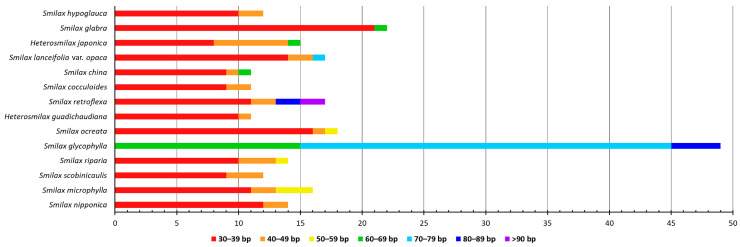
Length interval (bp) of LTRs detected from fourteen Smilacaceae species.

The identified LTRs were classified into seven length intervals (Figure 4). The cpGenome of *S. glycophylla* was again distinct from that of the others, which consisted of 15 LTRs in the length interval of 60–69 bp, 30 LTRs in 70–79 bp, and 4 LTRs in 80–89 bp. For the remaining thirteen cpGenomes, the majority of LTRs fell in the interval of 30–39 bp, from 8 (*H. japonica*) to 21 (*S. glabra*). Only the cpGenome of *S. retroflexa* had LTRs over 90 bp, which could be explored as potential markers for authenticating this species. 

### 2.3. Codon Usage Bias 

**Figure 5:**
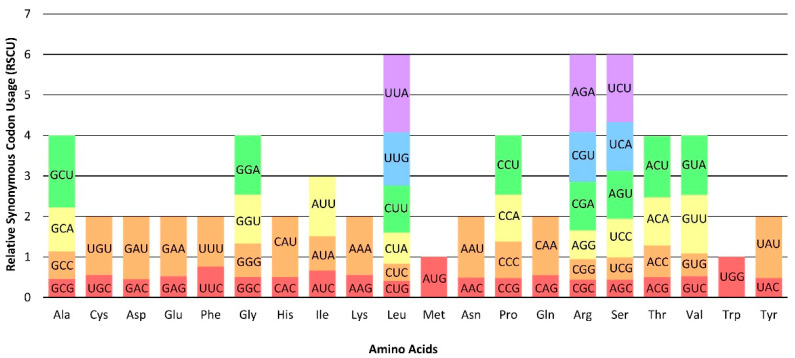
Codon usage in the fourteen Smilacaceae species.

Codon bias analysis was conducted for all fourteen Smilacaceae species. Except for methionine (Met) and tryptophan (Trp), the other amino acids showed a preference for two or more codons (Figure 5). Three amino acids, namely leucine (Leu), arginine (Arg), and serine (Ser), preferred six codons. Codon usage preferences were indicated by the relative synonymous codon usage (RSCU) value. Among the 64 codons, 30 of them were preferentially used, indicated by RSCU >1, while 32 of them were not preferred, as indicated by RSCU <1. The codons UUA for Leu and AGA for Arg had the highest RSCU of 1.91, indicating that these two codons were more preferred in the Smilacaceae species. The codon CUG for Leu had the lowest RSCU of 0.41, showing that this codon was not preferred in the Smilacaceae species.

### 2.4. Selection Pressure Analysis

A total of 78 PCGs commonly existed in the 14 Smilacaceae cpGenomes. The ratio of non-synonymous/synonymous substitution rate (Ka/Ks) indicated the selection of genes. Most of the PCGs were negatively selected, as shown by the Ka/Ks value being <1 (Table S1). Twelve PCGs showed positive selection in at least one pairwise comparison, including *rbcL*, *accD*, *rpl20*, *rpl14*, *ycf2*, *ndhF*, *cssA*, *ndhD*, *psaC*, *ndhE, ndhG*, and *ndhI*, indicated by the Ka/Ks value being >1. Certain genes were positively selected in the pairwise comparison of a particular species versus others. In the pairwise comparison between *S. scobinicaulis* versus the other thirteen cpGenomes, *rpl14* was positively selected, as supported by the Ka/Ks ranging from 1.45 to 1.97. Four PCGs, namely *psaC, ndhD*, *ndhE*, and *ndhG*, exhibited positive selection in the pairwise comparison between *S. cocculoides* and the others, with the values of Ka/Ks being greater than 1.9, 1.2, 1.3, and 1.5 accordingly. The greatest Ka/Ks value of 3.74 was found on *ndhF* in the pairwise comparison of *S. microphylla* versus *S. riparia*, showing that the gene was strongly positively selected between these two species. The gene *ndhI* only showed positive selection in one pairwise comparison, which was that of *S. ocreata* versus *S. glycophylla*, with Ka/Ks = 1.39.

### 2.5. Boundary and Structural Variation

**Figure 6:**
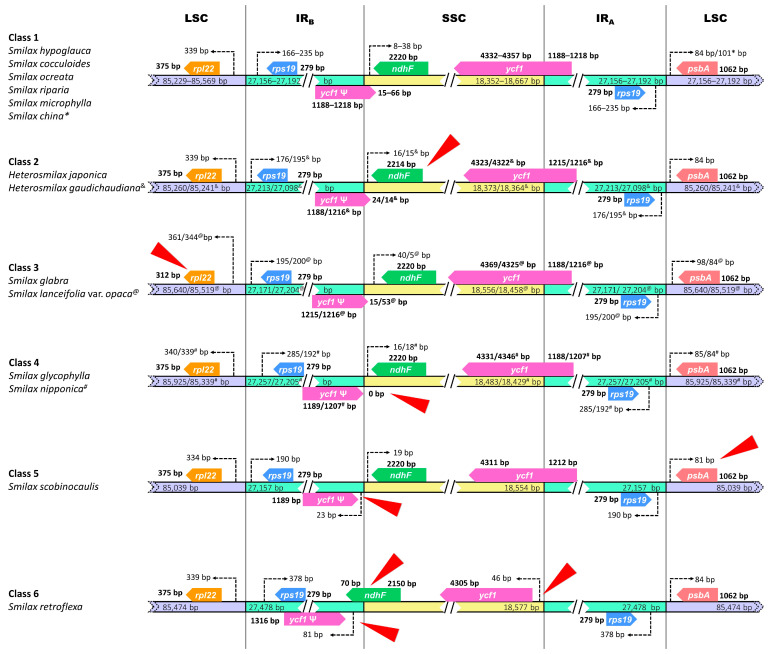
Boundary variations observed for the fourteen Smilacaceae species. The variations are highlighted with red triangles. The values marked with *, ^&^, ^@^, and ^#^ belong to *S. china*, *H. gaudichaudiana*, *S. lanceifolia* var. *opaca*, and *S. nipponica*, respectively.

Boundary comparison showed species-specific variations in border-flanking genes that were classified into six classes (Figure 6). Class 1 consisted of six species, including *S. hypoglauca*, *S. coculoides*, *S. ocreata*, *S. riparia*, *S. microphylla*, and *S. china*. Class 2 consisted of the two studied *Heterosmilax* species, where the gene *ndhF* at the IR_B_-SSC border had a size of 2214 bp, shorter than that of other *Smilax* species by 6 bp. Class 3 consisted of *S. glabra* and *S. lanceifolia* var. *opaca*, and the gene *rpl22* at the LSC-IR_B_ border had a size of 312 bp, shorter than those of other classes by 63 bp. The pseudogene *ycf1* at the IR_B_-SSC border showed variations in Classes 4 to 6. Class 4 consisted of *S. glycophylla* and *S. nipponica*, in which the short fragment of pseudogene *ycf1* in SSC was deleted, leaving no gap from the border. In Classes 5 (*S. scobinocaulis*) and 6 (*S. retroflexa*), gaps of 23 bp and 81 bp were found between the border and the pseudogene *ycf1*, respectively. Class 6 differed from Class 5 by the functioning *ycf1* gene at the SSC-IR_A_ border, which lost the short fragment in IR_A_, leaving a 46 bp gap from the border, and the gene *ndhF* crossed the IR_B_-SSC border. The gene *psbA* in Class 5 showed a gap of 81 bp from the IR_A_-LSC border, while the gap in Class 6 was 3 bp longer. The variation in the boundaries was also supported by the result of structural variation (Figure S5), where the four borders were significantly “cleaved” in the alignment visualization due to the low percentage of identity.

### 2.6. Divergence Hotspots

**Figure 7:**
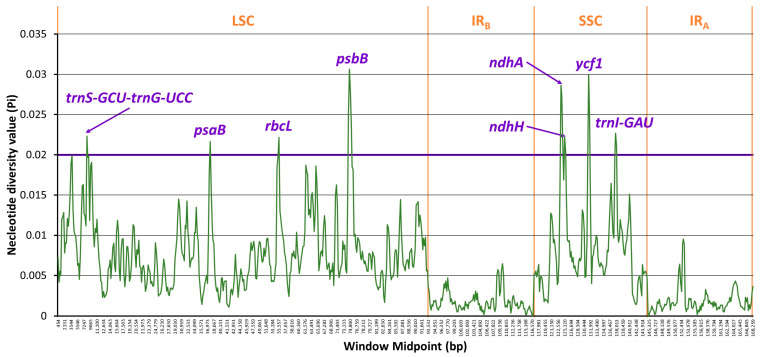
Sliding window analysis of eighteen Smilacaceae species revealing candidates of divergence hotspots. Threshold value of Pi = 0.02.

Eight divergence hotspots were identified with threshold Pi > 0.02, namely *trnS-GCU*-*trnG-UCC*, *psaB*, *rbcL*, *psbB*, *ndhA*, *ndhH*, *ycf1*, and *trnI-GAU* (Figure 7). None of them were located in IR regions. The highest Pi was found on *psbB* (Pi = 0.03064) in LSC. The locus *ycf1* in SSC showed the second-highest Pi value (Pi= 0.02996). These divergence hotspots could be explored as potential molecular markers in species authentication.

### 2.7. Phylogenomic Analysis

#### 2.7.1. Family Level

Nine cpGenomes of Smilacaceae available in NCBI Genbank were included in the phylogenomic analysis with the nine newly assembled ones. Six cpGenomes from Liliales, namely those of *Philesia magellanica* (NC_049020), *Ripogonum scandens* (NC_049021), *Lilium candidum* (NC_042399), *Tulipa gesneriana* (NC_063831), *Colchicum autumnale* (NC_030064), and *Veratrum grandiflorum* (NC_061622), were selected as outgroup species. 

**Figure 8:**
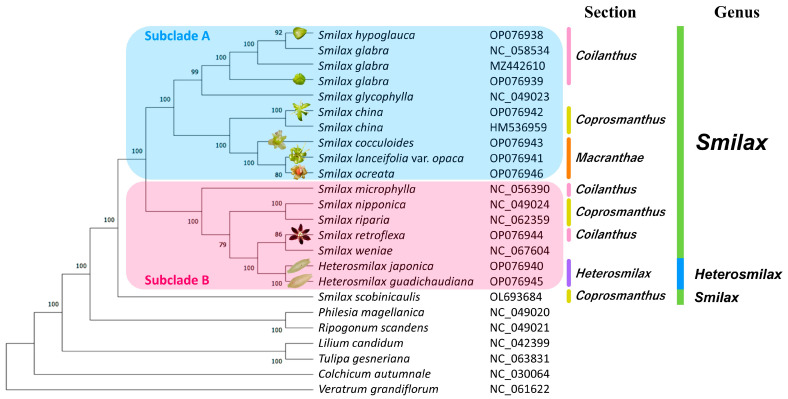
Phylogenomic analysis of eighteen Smilacaceae cpGenomes. The accessions of *Colchicum autumnale* (NC_030064), *Lilium candidum* (NC_042399), *Philesia magellanica* (NC_049020), *Ripogonum scandens* (NC_049021), *Tulipa gesneriana* (NC_063831), and *Veratrum grandiflorum* (NC_061622) were included as an outgroup. The bootstrap percentage (BP) is shown next to the node of branches. The section-level classification was referred to that of *Flora Reipublicae Popularis Sinicae* (FRPS). Accessions with flower images were newly assembled in this study. The flower images of the studied nine species were taken by T. Y. Siu and K. H. Wong, except for that of *S. ocreata*, which was taken from the Hong Kong Plant Database, AFCD, the Government of HKSAR (hyperlink: https://www.herbarium.gov.hk/en/hk-plant-database/plant-detail/index.html?pType=species&oID=9895 (accessed on 28 March 2023)). The flower images are not to scale.

The accession of *S. scobinicaulis* (OL693684) was sister to a large clade with strong support (Bootstrap Percentage (BP) = 100) (Figure 8). The large clade was divided into two well-supported subclades. Subclade A consisted of 10 cpGenomes, i.e., those of *S. hypoglauca*, *S. glabra* (NC_058534, MZ442610 and OP076939), *S. glycophylla*, *S. china* (OP076942 and HM536959), *S. cocculoides*, *S. lanceifolia* var. *opaca*, and *S. ocreata*. Subclade B included seven cpGenomes, i.e., those of *S. microphylla*, *S. nipponica*, *S. riparia*, *S. retroflexa*, *S. weniae*, *H. japonica*, and *H. gaudichaudiana*. 

Although the three accessions of *S. glabra* were clustered together, their phylogenetic positions were paraphyletic, as *S. hypoglauca* was embedded in the cluster with high bootstrap support (BP = 92). The sister clade of *Heterosmilax* species in subclade B, although well supported, with maximum support (BP = 100), was embedded in the *Smilax* cluster. 

All sectional divisions were not supported by the result of phylogenomic analysis, except for the sections *Macranthae* and *Heterosmilax*, which formed monophyletic clades. However, more cpGenomes of the sections *Macranthae* and *Heterosmilax* should be considered to verify their monophyly.

#### 2.7.2. Order Level 

**Figure 9:**
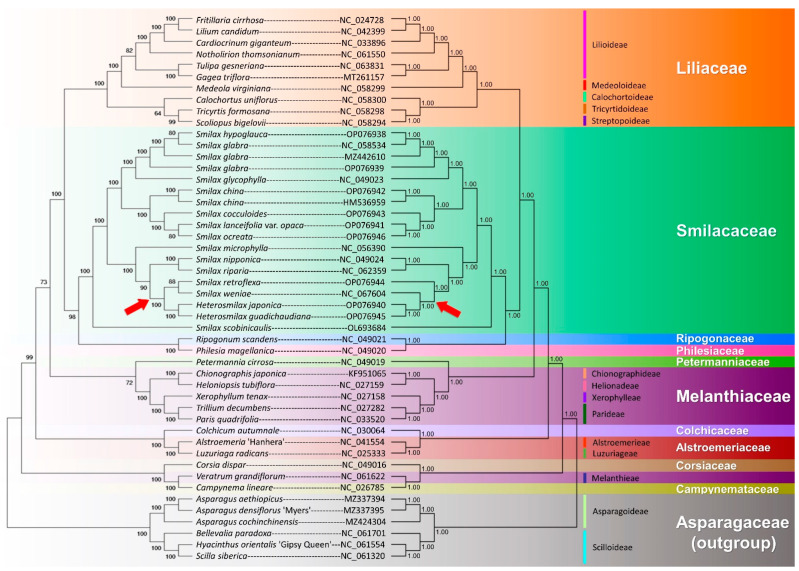
Phylogenomics analysis of forty-two Liliales cpGenomes. Six cpGenomes of Asparagales-Asparagaceae were selected as the outgroups. ML tree on the left. BI tree on the right. The bootstrap percentage (BP) and posterior probability (PP) are shown next to the nodes of the branches in the ML and BI trees, respectively. The right side of the BI tree presents the classified subfamilies (if stated) and families sensu APG IV for each accession. The arrows highlight the topological difference of the clade in the ML and BI trees.

Forty-four accessions of Liliales cpGenomes, representing the ten families of Liliales sensu APG IV, were integrated with six outgroups of Asparagales for phylogenomic analysis. Both the ML and BI trees showed almost the same topologies (Figure 9), except for the clade consisting of *S. retroflexa*, *S. weniae*, *H. japonica*, and *H. gaudichaudiana* (highlighted by red arrows). In the ML tree, the two *Smilax* species were clustered together in moderate support (BP = 88), and then sister to the two *Heterosmilax* species in high support (BP = 100). In contrast, in the BI tree, the two *Heterosmilax* species were sister to *S. weniae* and these three species were sister to *S. retroflexa*, with high support (PP = 1.00).

The cluster consisting of all Smilacaceae species was sister to a small cluster of two species, namely *Philesia magellanica* (Philesiaceaae) and *Ropogonum scandens* (Ripogonaceae). The large clade consisting of these three families was sister to the accessions from five subfamilies of Liliaceae sensu stricto. 

The phylogenetic position of *Veratrum grandiflorum* was unexpected. Classified under the family Melanthiaceae subfamily Melanthieae sensu APG IV, this species was expected to be clustered with the other five accessions of Melanthiaceae. Instead, it was embedded in the (*Corsia*, (*Veratrum*, *Campynema*)) clade in both the ML and BI trees, with the highest support (BP = 100; PP = 1.00). This “weird” phylogenetic position will be further discussed.

## 3. Discussion

### 3.1. The Chloroplast Genomes of Smilacaceae

The structure of Smilacaceae cpGenomes generally agreed with those of other photosynthetic land plants, as a quadripartite structure was observed. Identical numbers and contents of genes were observed in the newly assembled cpGenomes in this study, and similar GC contents (37.03–37.31%) were observed. The sizes of these cpGenomes were also comparable, from 157,885 bp to 159,007 bp. The distinctiveness of the LTRs in terms of quantity and length was observed in *S. glycophylla*, and they could serve as potential molecular markers to distinguish this species from the other thirteen. 

#### 3.1.1. Selection Pressure of PCGs and the Correlation with Environmental Factors

The most positively selected gene was *ndhD* in the pairwise comparison between *S. microphylla* and *S. riparia*, with Ka/Ks = 3.739. This PCG was also positively selected in *Nicotiana*, with an average Ka/Ks value of 6.181, probably for the adaptation of unknown environmental factors [44]. By overcoming specific environmental stresses, such as reduced light sources, the photosynthetic gene *ndhD* may be positively selected. According to the *Flora of China* (2000) [45], the habitats of *S. microphylla* include forests, thickets, and shaded places on slopes, whereas the habitats of *S. riparia* are forests, thickets, grassy slopes, and hillsides along valleys. This might imply differences in the amount of accessible light existing between the individuals of these two cpGenome accessions. 

Pairwise comparisons between a particular species and the other thirteen studied species showed positive selections in a few PCGs. Among these, *rpl14* showed positive selection in *S. scobinicaulis* versus the others (Ka/Ks = 1.452 to 1.973). Coding ribosomal protein L14, this PCG is involved in self-replication [46]. In the study by Li et al. in 2020 [46], *rpl14* was negatively selected (average Ka/Ks > 0.244) in the pairwise comparison between five non-alpine *Allium* species and the two alpine species collected above an elevation of 4000 m. The reason for the negative selection of this PCG may be related to the energy conversion efficiency in higher-altitude environments [46]. According to *Flora of China* (2000) [45], *S. scobinicaulis* inhabits elevations ranging from 600 to 1200 m, while the other studied species, excluding the Australian *S. glycophylla*, could be found near sea level to the altitude of 2200 m in China in similar habitats. The positive selection of *rpl14* showed no correlation with the elevation of habitats. The reason for this needs further investigation. 

In the pairwise comparison between *S. cocculoides* and the others, four PCGs were positively selected (Ka/Ks > 1.926 in *psaC*, >1.167 in *ndhD*, >1.308 in *ndhE*, and >1.511 in *ndhG*). These four photosynthetic PCGs code for photosystem I iron–sulfur centre subunit VII, NADH–plastoquinone oxidoreductase chain 4, NADH–plastoquinone oxidoreductase chain 4L, and NADH–plastoquinone oxidoreductase chain 6, respectively. It has been reported that *psaC* is a very conserved PCG in *Quercus* [47] and *Lilium* [48], as reflected by the Ka/Ks ratio being equal to zero, suggesting that the selection pressure of this gene was purified. *ndhG* was positively selected in *Paulownia* [49], and the enzyme coded by *ndhG* could protect higher plants from light and water stress [50]. In this study, the individual of *S. cocculoides* was located in close proximity to the population of *S. retroflexa*, which were both in a shaded forest in Sunset Peak. However, in the pairwise comparison of these two species, the Ka/Ks ratios of these PCGs (Ka/Ks = 2.119 in *psaC*, 1.241 in *ndhD*, 1.393 in *ndhE* and 1.565 in *ndhG*) were comparable to the Ka/Ks ratios in the pairwise comparison between *S. retroflexa* and the species collected in other locations. No correlation was observed between the positive selection of these four PCGs and the discrepancy in the habitat types with other species; instead, it tended to be species-specific for an unknown reason, which should be investigated in future. 

#### 3.1.2. Identification of Divergence Hotspots

Eight divergence hotspots, including *trnS-GCU*-*trnG-UCC*, *psaB*, *rbcL*, *psbB*, *ndhA*, *ndhH*, *ycf1*, and *trnI-GAU*, were identified (Figure 7). Among them, the locus *psbB* was the most variable, with a Pi value equal to 0.03064. This functional gene is responsible for coding the photosystem II (PSII) chlorophyll-binding protein and is not a frequent hotspot candidate in angiosperms. Instead, it has been reported as a divergence hotspot of *Pinus sylvestris* L., which is a gymnosperm [51]. The gene cluster *psbB*-*psbT*-*psbH*-*petB*-*petD* has been reported to be highly conserved among vascular plants [52]. However, the intergenic spacers between *psbB* and other PCGs were reported as hotspots of other angiosperms, such as *psbB*-*psbT* in *Bulbophyllum* (Pi = 0.12543) [53], *psbB*-*psbH* in *Lilium* (Pi = 0.01287) [54], and *rps12*-*psbB* in *Actinidia* (Pi = 0.03353) [55]. This hotspot region could be a reference for developing molecular markers for Smilacaceae species.

The second-most-variable hotspot was *ycf1*, with a Pi value equal to 0.02996. It showed species-specific variation across the borders (Figure 7). It is noteworthy that *ycf1* is also a hypervariable locus in the cpGenomes of angiosperm genera, including *Hedyotis* (Pi = 0.083) [35], *Dalbergia* (Pi = 0.037 for *ycf1a* and 0.032 for *ycf1b*) [56], *Asparagus* (Pi = 0.0154) [57], *Artemisia* (Pi ≅ 0.09) [58], *Allium* (Pi = 0.03716) [59], *Primula* (Pi = 0.05036) [60], and *Aponogeton* (Pi ≅ 0.0225) [61]. However, the sequence of *ycf1* over a few thousand base pairs would be too long for polymerase chain reaction (PCR) amplification. It was not chosen for primer design due to the high variability in the case of *Hedyotis* [35]. In the case of the Smilacaceae, the aligned *ycf1* sequences (File S1) also showed hypervariable nucleotides across almost the full length; hence, the locus might not be an ideal candidate barcode marker due to difficulties in primer design. 

### 3.2. The Status and Taxonomical Revision of Heterosmilax

Previous phylogenetic studies based on morphological [6,22] and molecular [1,22] data have shown that the genus *Heterosmilax* is nested within the genus *Smilax*, with a close morphological relationship with the *Smilax* section *Coilanthus* [6,17]. In particular, polyphyly of *Heterosmilax* was found in the study by Cameron and Fu (2006) [1] based on nuclear ITS data. In this study, although the two accessions of *Heterosmilax* cpGenomes formed a monophyletic clade, the limited sample number did not support the monophyly of the genus *Heterosmilax*. Further, they were nested within the *Smilax* species, that is consistent with the studies by Chen et al. (2006) [6] and Qi et al. (2013) [22], which were based on solely morphological and molecular (ITS + *matK* + *rpl16*) plus morphological data, respectively. However, *Heterosmilax* did not show a close relationship with the *Smilax* section *Coilanthus* (*S. hypoglauca*, *S. glabra*, *S. microphylla*, and *S. retroflexa*) (Figure 8), contrasting the work by Chen et al. (2006) [6] and Koyama (1984) [17]. 

It is clear that the genus *Heterosmilax* should be downranked; however, whether it should be treated as a subgenus or a section is arguable, as different practices have been adopted by different botanists. Referring to Dr George K. Brizicky’s article *Subgeneric and Sectional Names Their Starting Points and Early Sources* published in 1969, “subgeneric and sectional names must be interpreted in terms of the word actually used to denote the rank of the taxon to which the name is applied and not in terms of the author’s concept of infrageneric categories; what the author actually did is of greater importance than his guessed intentions” [62]. Therefore, it is suggested to follow the practice of Judd in 1998 [20], treating *Heterosmilax* as a section of the genus *Smilax*, according to nomenclatural priority. 

Accordingly, the names of the species originally classified under the genus *Heterosmilax* have to be transferred to the genus *Smilax*. The two *Heterosmilax* species found in Hong Kong, namely *Heterosmilax gaudichaudiana* (Kunth) Maxim. and *Heterosmilax japonica* Kunth, are still named under the genus *Heterosmilax* Kunth in the *Flora of Hong Kong* [63] and in the online Hong Kong Plant Database [64]. Various practices have been undertaken in different online plant databases. For *Heterosmilax gaudichaudiana* (Kunth) Maxim., the International Plant Names Index (IPNI) [65], Plants of the World (POWO) [66], and World Flora Online (WFO) [67] all resurrect the name *Smilax gaudichaudiana* Kunth. For *Heterosmilax japonica* Kunth, POWO [68] and WFO [69] combine the species under *Smilax bockii* Warb. and *Smilax bockii* Warb. ex Diels., respectively. IPNI combines it under the nomen novum *Smilax goeringii* Kladwong, Chantar. & D. A. Simpson [70], while the online version of *Flora Reipublicae Popularis Sinicae* (FRPS) [71] uses the combinatio nova *Smilax japonica* (Kunth) P. Li & C. X. Fu, which was proposed by Qi et al. in 2013 [19]. However, the latter was later found to be illegitimate by Kladwong et al. in 2018 [72], as Qi et al. missed *Smilax japonica* (Kunth) A. Gray (1858), which was combined into the current *Smilax china* L. [73], causing a later homonym. In contrast to all of the above databases, Tropicos still accepts *Heterosmilax gaudichaudiana* (Kunth) Maxim. [74] and *Heterosmilax japonica* Kunth [75]. The nomenclatural revisions of *Heterosmilax* species require further discussion.

### 3.3. The Phylogenomics of Liliales 

The phylogenomic analysis at the order level showed a degree of difference with the previous study performed by Do et al. in 2020 [39]. A sister relationship between the Smilacaceae and Liliaceae was observed in their study, with the posterior probability being 0.85 in the BI tree and the bootstrap percentage being 98 in the ML tree constructed using 78 plastid PCGs [39]. In contrast, sister relationships of Liliaceae and (Smilacaceae, (Philesiaceae, Ripogonaceae)) were observed in this study, with maximum support values (PP = 1.00, BP = 100). Such a relationship was also observed and strongly supported in the phylogenetic trees constructed using four plastid loci (*matK*, *rbcL*, *atpB*, and *atpF-atpH*) (PP = 1; BP = 97) in the study by Kim et al. (2013) [76]. The Smilacaceae species appeared to form a distinct clade from the “true-lilies”, Liliaceae sensu stricto, further strengthening the family status of Smilacaceae and the abandonment of the circumscription of Liliaceae sensu lato.

The genus *Ripogonum* J.R. Forst. & G. Forst. has been treated as a member of Smilacaceae in several classification systems including Bantham and Hooker in 1883 [8], Hutchinson in 1959 [11] and 1973 [77], Dahlgren et al. in 1985 [16], and Judd et al. in 2002 [78]. However, based on molecular data, this genus was separated from the Smilacaceae, and placed under the monotypic family Ripogonaceae by Conran and Clifford in 1985 [79]. This was later adopted by other botanists, including Takhtajan in 1997 [3] and 2009 [80], Thorne in 1992 [81], and Judd et al. in 2008 [82]. In the phylogenetic trees of this study, *Ripogonum* was clearly separated from the clusters of Smilacaceae, further supporting the exclusion of this genus from the Smilacaceae. In fact, Chen et al. studied the Smilacaceae based on morphological [6], palynological [5], and seed coat [21] data, which all supported the status of the Ripogonacaceae. Do et al. suggested the inclusion of the Ripogonacaceae in the Philesiaceae [39]. Although a sister relationship of the accessions from these two families was observed in this study based on cpGenome data, due to the limited sample size and data, this treatment could neither be supported nor rejected. 

In the phylogenetic trees, *Veratrum* (Melanthieae, Melanthiaceae) was found to lay outside of the clade formed by other members of Melanthiaceae. The possible reason for this is likely to be the inclusion of *Campynema* and *Corsia* in the dataset. In the phylogenetic trees of this study, the evergreen *Campynema lineara* was closely related to *Veratrum grandiflorum* (Figure 9). In the strict consensus tree of Rudall et al. (2000) [83] combined with morphological and molecular (*trnL-trnF* and *rbcL*) data, a sister relationship of *Campynema* with the clade (Liliaceae, (*Smilax,* (*Philesia*, *Ripogonum*))) was found, in which the large complex was sister to Melanthiaceae, and not in the basal position of the Liliales. Rudall et al. pointed out that, although the Australian family Campynemataceae was clearly lilioid, supported by the absence of septal nectaries, the relationship between it and other Liliales was still “equivocal” [83]. It is noteworthy that the Campynemataceae were placed under the Melanthiales by Dahlgren et al. in 1985 [84], and the family was described as an intermediate between the Melanthiales and Burmanniales (the current Dioscoreales) which should not be associated with the Liliales as raphides are present. Goldblatt (1995) [85] conducted a cladistic analysis to review the order Liliales and Melanthiales sensu Dahlgren et al. (1985) based on a data matrix of 23 morphological characters with reference to previous *rbcL* data. The result showed that the Campynemataceae were closer to the core Liliales families, which should be further studied. Our chloroplast genome phylogeny shows a close relationship between *Veratrum* and *Campynema* (BP = 100; PP = 1.00), supporting Dahlgren et al.’s treatment, to a certain degree.

The inclusion of the Corsiaceae in the Liliales was first seen in the APG II system in 2003 [86] based on 26S rDNA data [87], followed by Fay et al. in 2006 [88], based on the combined matrix of plastid *rbcL*, *trnL*-intron, *trnL-trnF*, *matK*, *ndhF* and mitochondrial *atp1*. This mycoheterotrophic family was previously placed under the Burmanniales by Hutchinson (1959) [11] and Dahlgren et al. (1985) [89], and was once unplaced from any order by Chase et al. (2000) [90]. Still, Fay et al. emphasised that the tentative placement of the Corsiaceae in the Liliales would still be “problematic” until its monophyly could be verified [88]. The polyphyly of the Corsiaceae was inferred from partial 26S rDNA sequences [91] and morphological [92] data. In 2015, Mennes et al. [93] inferred the monophyly of the Corsiaceae and its sister relationship with the Campynemataceae using three loci, namely nuclear 18S rDNA and mitochondrial *atpA* and *matR*, based on both BI and ML analyses with maximum support (PP = 1; BP = 100). Meanwhile, the Melanthiaceae (*Veratrum* and *Trillium*) were found to be the sister group of the complex consisting of the Corsiaceae (*Corsia* and *Arachnitis*) and Campynemataceae (*Campynema* and *Capynemathe*), showing that these three families would be closely related. However, the study by Mennes et al. (2015) [93] did not combine the limited nuclear and mitochondrial data into the plastid matrix of 82 PCGs for analysis.

As the placement of the Campynemataceae and Corsiaceae in the Liliales has been questioned by botanists, we further analysed the typology of the phylogenetic tree by removing the accessions of *Campynema lineare* (NC_026785) and *Corsia dispar* (NC_049016) from the data set. The accession of *Veratrum grandiflorum* (NC_061622) was then sister to its relatives from the Melanthiaceae in both the ML tree (Figure S2) and BI tree (Figure S3), with high support (BP = 100; PP = 1.00). However, further studies are urgently needed, particularly those enriching nuclear and mitochondrial data, to resolve the ambiguity of taxonomical placement and phylogenetic positions of these taxa.

## 4. Methods

### 4.1. Plant Materials and DNA Extraction

**Figure 10:**
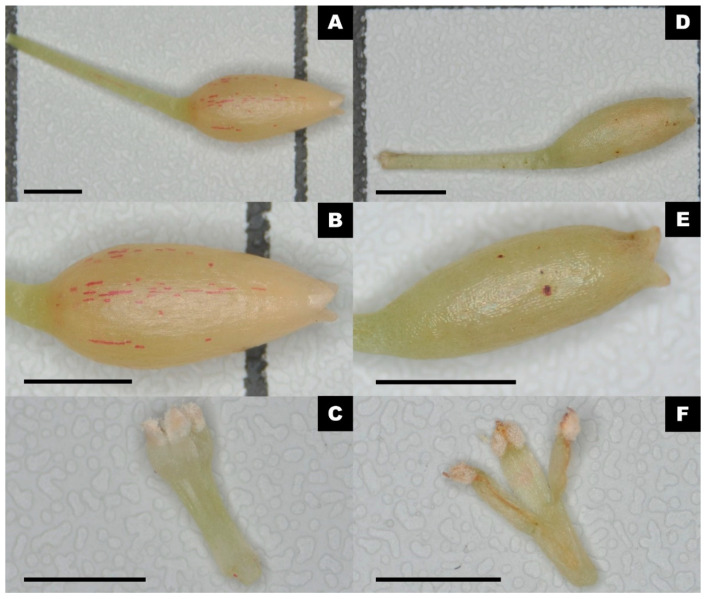
Floral dissection of *Heterosmilax gaudichaudiana* (Kunth) Maxim. and *Heterosmilax japonica* Kunth. (**A**–**C**), *H. gaudichaudiana*, (**D**–**F**), and *H. japonica*. (**A**,**D**); Flowers including the pedicle (scale bar = 0.2 cm). (**B**,**E**) Corolla (scale bar = 0.2 cm). (**C**,**F**) Stamen (scale bar = 0.1 cm). The filament of *H. gaudichaudiana* is nearly fused for its full length as a single column, while the filament of *H. japonica* is fused at its lower half.

Voucher specimens with plant tissue samples of the nine studied Smilacaceae species were legally collected in Hong Kong from February 2020 to July 2021 (Table 1, Figure 10). All collectors in this study were authorised to collect plant materials in Hong Kong Country Parks with the *Permission to Make Field Collection for Research Purpose* issued by AFCD of HKSAR Government. All specimens were authenticated according to *Flora of Hong Kong* Volume 4 [63], *Flora Reipublicae Popularis Sinicae* Volume 15 [94], and *Flora of China* Volume 24 [45]. All voucher specimens were deposited in the Shiu-Ying Hu Herbarium (herbarium code: CUHK), School of Life Sciences, Chinese University of Hong Kong, China. Figure S4 includes the digitised voucher specimens. The authenticating characteristics of *H. gaudichaudiana* and *H. japonica* are shown in Figure 10. 

Plant tissue samples were stored at −80 °C until their use in DNA extraction. Around 50 mg of healthy leaves was cut into small pieces and then homogenised using Precellys^®^ Evolution (Bertin, Montigny-le-Bretonneux, France). The total genomic DNA was extracted using an i-genomic Plant DNA Extraction Mini Kit (iNtRON Biotechnology, Daejeon, Republic of Korea) following the instructions of the manufacturer. The quality of the extracted DNA was checked using 1.5% agarose gel electrophoresis, while the quantity of DNA was measured using a NanoDrop Lite Spectrophotometer (Thermo Fisher Scientific, Waltham, MA, USA). Shotgun sequencing of the qualified genomic DNA was conducted by Novogene Bioinformatic Technology Co. Ltd. (http://en.novogene.com/ (accessed on 28 March 2023), Beijing, China). 

### 4.2. Genome Sequencing, Assembly and Annotation

The NovaSeq 6000 platform (Illumina Inc., San Diego, CA, USA) was used to construct and sequence paired-end libraries, with an insert size of 150 bp. CpGenomes were assembled using CLC Assembly Cell 5.1.1 (CLC Inc., Aarhus, Denmark). Three to four gigabytes (GB) of raw reads per sample (Table 1) were paired up and quality-trimmed with a Phred score below 33. A CLC de novo assembler was used in contig assembly. Gapcloser in SOAPdenovo 3.23 was used to fill gaps. NUCmer 3.0 was used to retrieve and order the contigs, which were aligned against the reference cpGenome (*S. china*, NC_049022). The NCBI blastn suite (https://blast.ncbi.nlm.nih.gov/Blast.cgi?PROGRAM=blastn&PAGE_TYPE=BlastSearch&LINK_LOC=blasthome (accessed on 28 March 2023)) was used to check the directionality and coverage of contigs. The aligned contigs with high query coverage were then connected into a complete cpGenome with directionality adjustment, further mapping, and manual corrections.

The GeSeq platform (https://chlorobox.mpimp-golm.mpg.de/geseq.html (accessed on 28 March 2023)) [95] was used to perform gene annotation on the chloroplast sequences. Two NCBI-verified cpGenomes, namely *S. china* (NC_049022) and *S. nipponica* (NC_049024), were selected as reference genomes for gene annotation. The positions of the exons and introns were manually corrected when necessary. OrganellarGenomeDRAW (OGDRAW) [96] was used to draw the circular genome maps (Figure S1). The annotated cpGenomes were submitted to NCBI GenBank with the accession numbers OP076938 to OP076946 (Table 1).

### 4.3. Genome Content and Structural Analysis

Analyses of the content and structural analysis were performed on cpGenomes of fourteen Smilacaceae species. Apart from the nine newly assembled cpGenomes (Table 1), another five cpGenomes available in NCBI GenBank were selected for analysis, including *S. glycophylla* (NC_049023), *S. riparia* (NC_062359), *S. scobinicaulis* (OL693684), *S. microphylla* (NC_056390), and *S. nipponica* (NC_049024). Each species was represented by one cpGenome. As a voucher specimen was absent for the previously published accession HM536959 (*S. china*) and the voucher specimen for NC_058534 = MZ566572 (*S. glabra*) was inaccessible, the newly assembled accessions OP076942 and OP076939 were selected to represent these two species, and the authentications were supported by our voucher specimens (Figure S4). The stated species of the accession OL693684 was “*Smilax moranensis*”, which is native from Mexico to Nicaragua [97], without record in China [45]. Referring to the publication of Ji et al. in 2022 [42] reporting OL693684, the sample was collected from Henan, China. Meanwhile, the reference list included studies related to *S. scobinicaulis*. When the authors searched “*Smilax scobinicaulis*” in the NCBI Taxonomy database [98] (https://www.ncbi.nlm.nih.gov/taxonomy/?term=Smilax+scobinicaulis (accessed on 28 March 2023)), the system showed “*Smilax moranensis*” (Taxonomy ID: 1080332) instead. As *S. scobinicaulis* [99] and *Smilax moranensis* M. Martens & Galeotti [97] are accepted species without overlapping distributions, the accession OL693684 is treated as a representative of *S. scobinicaulis* throughout this paper. 

#### 4.3.1. Sequence Repeats Analysis

MIcroSAtellite identification tool (MISA, https://webblast.ipk-gatersleben.de/misa/index.php?action=1 (accessed on 28 March 2023)) [100] was used to detect simple sequence repeats (SSRs). Mono-, di, tri-, tetra-, penta-, and hexa-nucleotide repeats were screened by setting the minimum numbers of repetitions to 10, 5, 4, 3, 3, and 3, respectively.

REPuter (https://bibiserv.cebitec.uni-bielefeld.de/reputer (accessed on 28 March 2023)) [101] was used to identify long tandem repeats (LTRs) classified as forward, reverse, complement, and palindromic sequences under the condition of a 50 bp maximum computed repeat size and 30 bp minimum repeat size.

#### 4.3.2. Boundary and Structural Variation Analysis

A diagram of the boundary variation was manually drawn using the results of annotation, including the size and position of border-flanking genes and the length of each compartment within a cpGenome.

Structural variations were visualised (Figure S5) using mVISTA (https://genome.lbl.gov/vista/mvista/submit.shtml (accessed on 28 March 2023)) [102]. The cpGenome of *S. hypoglauca* (OP076938) was taken as reference. The alignment program Shuffle-LAGAN [103] was chosen.

#### 4.3.3. Codon Usage Bias and Selection Pressure Analysis

The protein-coding genes (PCGs) were extracted using FeatureExtract version 1.2 (https://services.healthtech.dtu.dk/service.php?FeatureExtract-1.2 (accessed on 28 March 2023)) [104]. 

The PCGs of each cpGenome were concatenated into a single sequence and aligned by MAFFT 7 (https://mafft.cbrc.jp/alignment/server/ (accessed on 28 March 2023)) [105]. Codon usage bias was tested using DNA Sequence Polymorphism (DnaSP) version 6.12.03 [106], which generated the average codon frequency and RSCU for each codon of amino acids. 

The rates of synonymous (Ks) and non-synonymous (Ka) substitution of each commonly shared PCG were calculated by DnaSP version 6.12.03 [106] in pairwise comparisons between the fourteen cpGenomes. The Ka/Ks ratios were then calculated for each comparison by dividing the Ka values by the Ks values. The ratios with Ks equal to zero were classified as “Undefined”. Positive and negative selections are visualised in Table S1 by applying conditional formatting with a three-color scale (R248B105G107 for the minima, R255B255G255 for Ka/Ks equal to one, and R90B138G198 for the maxima). 

#### 4.3.4. Nucleotide Diversity Analysis

Nine complete cpGenomes of Smilacaceae publicly available in NCBI GenBank, including *S. china* (HM536959), *S. scobinicaulis* (OL693684), *S. nipponica* (NC_049024 = MT261170), *S. microphylla* (NC_056390 = MW423607), *S. glycophylla* (NC_049023 = MT261169), *S. riparia* (NC_062359 = OK244690), *S. weniae* (NC_067604), and two for *S. glabra* (MZ442610, NC_058534 = MZ566572) were aligned with the nine newly assembled cpGenomes using MAFFT 7 [105]. The nucleotide diversity values (Pi) from the aligned sequences were calculated using DnaSP version 6.12.03 [106], with the condition of a 600 bp window length and 200 bp step size. 

### 4.4. Phylogenomic Analysis

Phylogenomic analysis was performed at both the family level and order level to infer the infra- and inter-familial phylogeny of the Smilacaceae, respectively. 

#### 4.4.1. Family Level

Eighteen cpGenomes of the Smilacaceae, including the nine newly assembled and the nine NCBI GenBank-available cpGenomes, were aligned with the six outgroup accessions, namely *Colchicum autumnale* (NC_030064), *Lilium candidum* (NC_042399), *Philesia magellanica* (NC_049020), *Ripogonum scandens* (NC_049021), *Tulipa gesneriana* (NC_063831), and *Veratrum grandiflorum* (NC_061622), using MAFFT 7. A maximum likelihood (ML) tree was constructed using MEGA-X version 10.2.5 [107]. The general time reversible (GTR) model and gamma distributed with invariant sites (G + I) were selected as the substitution model and the rates among sites, respectively, as this combination had the lowest Bayesian information criterion (BIC) scores in Model Selection of Mega X. The bootstrap replicates were set to 1000.

#### 4.4.2. Order Level

Forty-two cpGenomes of Liliales, including the nine newly assembled and thirty-three NCBI GenBank-available cpGenomes, were selected to represent eleven families of Liliales. They were aligned with six outgroup cpGenomes from the Asparagales–Asparagaceae, namely *Asparagus aethiopicus* (MZ337394), *A. densiflorus* ‘Myers’ (MZ337395), *A. cochinchinensis* (MZ424304), *Bellevalia paradoxa* (NC_061701 = OM320811), *Hyacinthus orientalis* ‘Gipsy Queen’ (NC_061554 = OM320803), and *Scilla siberica* (NC_061320 = OM320810) using MAFFT 7. An ML tree was constructed using MEGA-X version 10.2.5. The best-fit nucleotide substitution model, GTR + G + I, was selected, as it had the lowest BIC scores. A Bayesian inference (BI) tree was constructed using BEAST version 1.10.4 (https://beast.community/ (accessed on 28 March 2023)) [108] with default settings, except the substitution model, site heterogeneity model and tree prior were set to “GTR”, “Gamma + Invariant Sites”, and “Yule Process” [109,110], respectively. FigTree version 1.4.4 (http://tree.bio.ed.ac.uk/software/figtree/ (accessed on 28 March 2023)) was used to visualise the BI tree. 

## 5. Conclusions

In this study, cpGenomes were assembled for the nine Smilacaceae species in Hong Kong. The structure and content of these cpGenomes were relatively conserved. Integrated with another five Smilacaceae available in NCBI GenBank, sequence repeats, codon usage biases, selection pressures of PCGs, boundary variations, and divergence hotspots were analysed for fourteen Smilacaceae species. The distinctiveness of long tandem repeats (LTRs) of *S. glycophylla* could be used to develop molecular markers to distinguish this species from the other *Smilax* members. The positive selection for *ndhD* implies differences in the light sources accessed by individual plants. After including the cpGenomes of additional Smilacaceae and Liliales species for phylogenomic analysis, the infra- and inter-familial relationships among the Smilacaceae were revisited and discussed. Nesting within the *Smilax* cluster in phylogenetic trees, the generic status of *Heterosmilax* was not supported, echoing previous molecular and morphological studies. The generic delimitation of the genus *Heterosmilax* Kunth as *Smilax* L. section *Heterosmilax* was reemphasised. Meanwhile, the monophyly of Smilacaceae was observed in a distinct clade from the Liliaceae sensu stricto, further fortifying the status of this family. The exclusion of *Ripogonum* from the Smilacaceae is supported by the distinct clustering from Smilacaceae. A close relationship between *Veratrum* and *Campynema* supported the treatment of placing the Campynemataceae under *Malanthiales* sensu Dahlgren et al. (1985). However, further studies of the placement of the autotrophic Campynemataceae and the heterotropic Corsiaceae are urgently needed. This study contributes to the systematics and taxonomy of the monocots, authentication of medicinal Smilacaceae resources, and conservation of plant diversity in Hong Kong and worldwide.

## Figures and Tables

**Table 1:** Information of the nine newly assembled and annotated chloroplast genomes.

Accession	OP076938	OP076939	OP076940	OP076941	OP076942	OP076943	OP076944	OP076945	OP076946
*Information of the vouchers*									
Species	*Smilax hypoglauca* Benth.	*Smilax glabra* Roxb.	*Heterosmilax japonica* Kunth	*Smilax lanceifolia* Roxb. var. *opaca* A. DC.	*Smilax china* L.	*Smilax cocculoides* Warb.	*Smilax retroflexa* (F. T. Wang & Tang) S. C. Chen	*Heterosmilax guadichaudiana* (Kunth) Maxim.	*Smilax ocreata* A. DC.
Collector Number	T. Y. Siu 426	T. Y. Siu 658	T. Y. Siu 812	K. H. Wong 150	K. H. Wong 154	K. H. Wong 160	K. H. Wong 162	K. H. Wong 169	K. H. Wong 176
Collection date	1 February 2020	4 November 2020	12 July 2021	4 March 2021	4 March 2021	16 March 2021	16 March 2021	7 June 2021	16 July 2021
Inventory no.	CUSLSH2478	CUSLSH2797	CUSLSH3069	CUSLSH2953	CUSLSH2957	CUSLSH2972	CUSLSH2974	CUSLSH3033	CUSLSH3071
Sheet no.	CUHK5490	CUHK5496	CUHK6147	CUHK6148–CUHK6150	CUHK6151–CUHK6152	CUHK6153–CUHK6154	CUHK6155–CUHK6157	CUHK6158	CUHK6159–CUHK6168
Location	Ma On Shan	Keung Shan	Lantau Peak	Tai Mo Shan	Tai Mo Shan	Sunset Peak	Sunset Peak	CUHK	Tsuen Wan
Sex	Female	Female	Male	Female	Female	Female	Male	Male	Female
*Information of the genomic data*									
Raw data (GB)	3.8	3.0	3.2	3.8	3.4	4.0	3.7	3.8	3.8
Coverage (×)	456	110	169	155	143	134	192	95	151
*Information of the assembly and annotation*									
Genome size (bp)	158,118	158,538	158,059	158,385	158,269	158,418	159,007	157,885	158,223
LSC size (bp)	85,410	85,640	85,260	85,519	85,433	85,566	85,474	85,241	85,469
SSC size (bp)	18,352	18,556	18,373	18,458	18,524	18,478	18,577	18,364	18,370
IR size (bp)	27,178	27,171	27,213	27,204	27,156	27,187	27,478	27,098	27,192
Gene number	132	132	132	132	132	132	132	132	132
mRNA	86	86	86	86	86	86	86	86	86
tRNA	38	38	38	38	38	38	38	38	38
rRNA	8	8	8	8	8	8	8	8	8
ORF	4	4	4	4	4	4	4	4	4
1-intron gene	18	18	18	18	18	18	18	18	18
2-intron gene	2	2	2	2	2	2	2	2	2
Trans-spliced gene	1	1	1	1	1	1	1	1	1
GC content	37.07%	37.03%	37.31%	37.11%	37.15%	37.10%	37.10%	37.29%	37.14%

**Table 2:** Annotated genes in the nine Smilacaceae chloroplast genomes.

Gene Category	Gene Function	Gene Name
Photosynthesis-related genes	Rubisco	*rbcL*
	Photosystem I	*psaA, psaB, psaC, psaI, psaJ*
	Assembly/stability of photosystem I	pafI **, pafII, pbf1
	Photosystem II	*psbA, psbB, psbC, psbD, psbE, psbF, psbH, psbI, psbJ, psbK, psbL, psbM, psbT, psbZ*
	ATP synthase	atpA, atpB, atpE, atpF *, atpH, atpI
	Cytochrome b/f complex	petA, petB *, petD *, petG, petL, petN
	Cytochrome c synthesis	*ccsA*
	NADPH dehydrogenase	ndhA *, ndhB * (x2), ndhC, ndhD, ndhE, ndhF, ndhG, ndhH, ndhI, ndhJ, ndhK
Transcription- and translation-related genes	Transcription	rpoA, rpoB, rpoC1 *, rpoC2
	Ribosomal protein	rpl14, rpl16 *, rpl2 * (x2), rpl20, rpl22, rpl23 (x2), rpl32, rpl33, rpl36, rps2, rps3, rps4, rps7 (x2), rps8, rps11, rps12 * (x2, trans-spliced), rps14, rps15, rps16 *, rps18, rps19 (x2)
RNA genes	Transfer RNA	trnA-UGC * (x2), trnC-GCA, trnD-GUC, trnE-UUC, trnF-GAA, trnfM-CAU, trnG-GCC, trnG-UCC *, trnH-GUG (x2), trnI-CAU (x2), trnI-GAU * (x2) ^, trnK-UUU *, trnL-CAA (x2), trnL-UAA *, trnL-UAG, trnM-CAU, trnN-GUU (x2), trnP-UGG, trnQ-UUG, trnR-ACG (x2), trnR-UCU, trnS-GCU, trnS-GGA, trnS-UGA, trnT-GGU, trnT-UGU, trnV-GAC (x2), trnV-UAC *, trnW-CCA, trnY-GUA
	Ribosomal RNA	*rrn16 (x2), rrn23 (x2), rrn4.5 (x2), rrn5 (x2)*
Miscellaneous group	Maturase	*matK*
	Inner membrane protein	*cemA*
	ATP-dependent protease	clpP1 **
	Acetyl-CoA carboxylase	*accD*
	Unknown functions	*ycf1 (x2), ycf2 (x2)*

*^*: The cope in IR_B_ is a fragment in *H. gaudichaudiana* (OP076945). *: Gene containing one intron. **: Gene containing two introns.

## Data Availability

The annotated chloroplast genome sequences were deposited in National Center for Biotechnology Information (NCBI) GenBank (https://www.ncbi.nlm.nih.gov/, accessed on 28 March 2023)) under the accession numbers OP076938 to OP076946. All voucher specimens were deposited in Shiu-Ying Hu Herbarium (herbarium code: CUHK), and the digitised specimens were supplemented in Figure S3.

## Supplementary Materials

The following supporting information can be downloaded at: https://www.mdpi.com/article/10.3390/ijms24087460/s1.
