# Supplementary material for: The Complete Chloroplast Genomes of Nine Smilacaceae Species from Hong Kong: Inferring Infra- and Inter-Familial Phylogeny
Source: Int J Mol Sci. 2023 Apr 18;24(8):7460. doi: 10.3390/ijms24087460 (PMC10138973; doi:10.3390/ijms24087460)
Supplement: Supplementary file 1 [file ijms-24-07460-s001.zip › Figure S1 to S5.pdf]

Figure S1a. Circular map of the chloroplast genome of *Smilax hypoglauca* (OP076938)

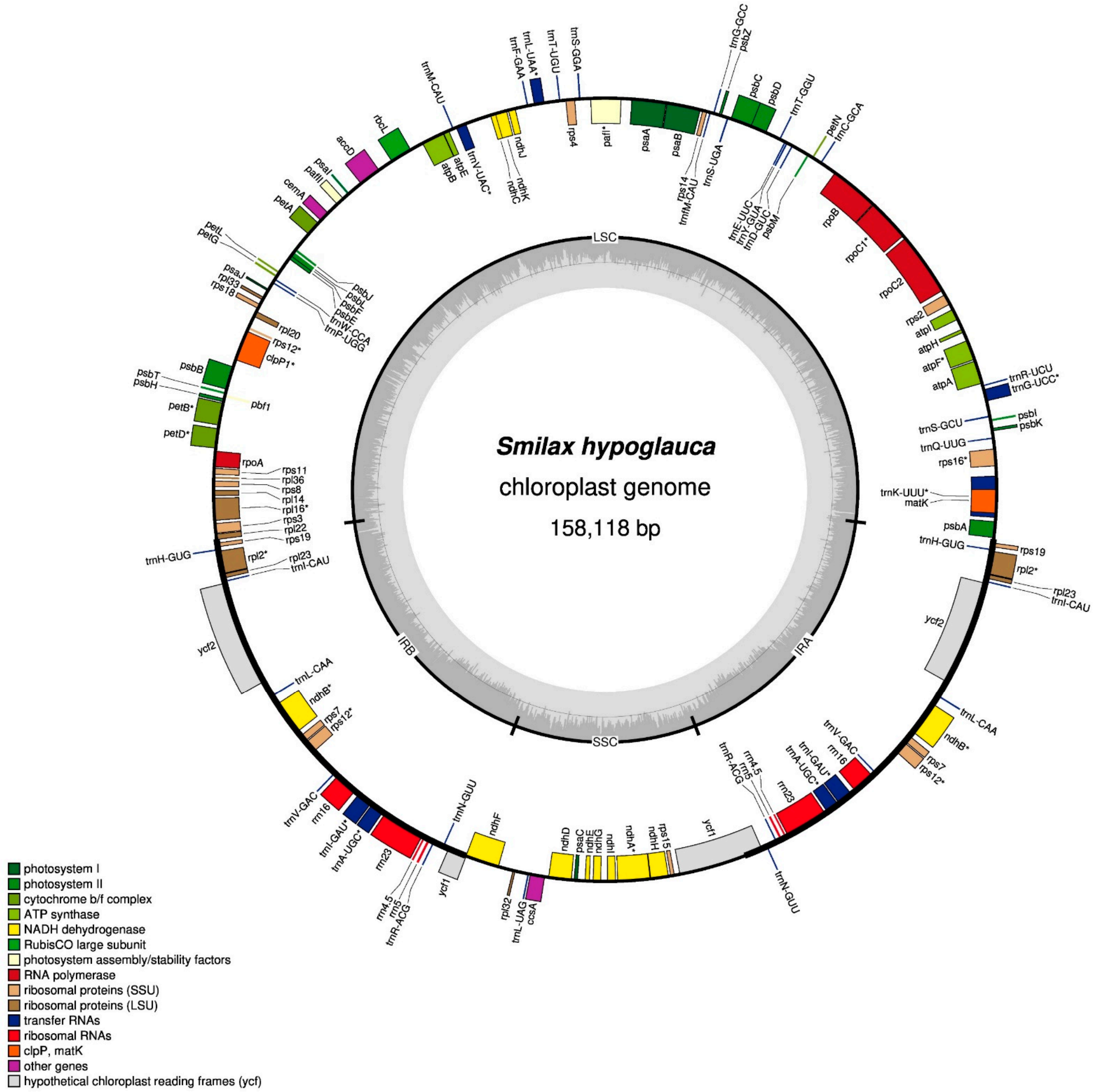

Genes are color-coded based on their functions shown in the key. Genes located outside of the outer circle are transcribed anticlockwise, while those insides are transcribed clockwise. In the inner circle, the gradient in dark grey represents GC content, whereas light grey represents AT content.

Figure S1b. Circular map of the chloroplast genome of *Smilax glabra* (OP076939)

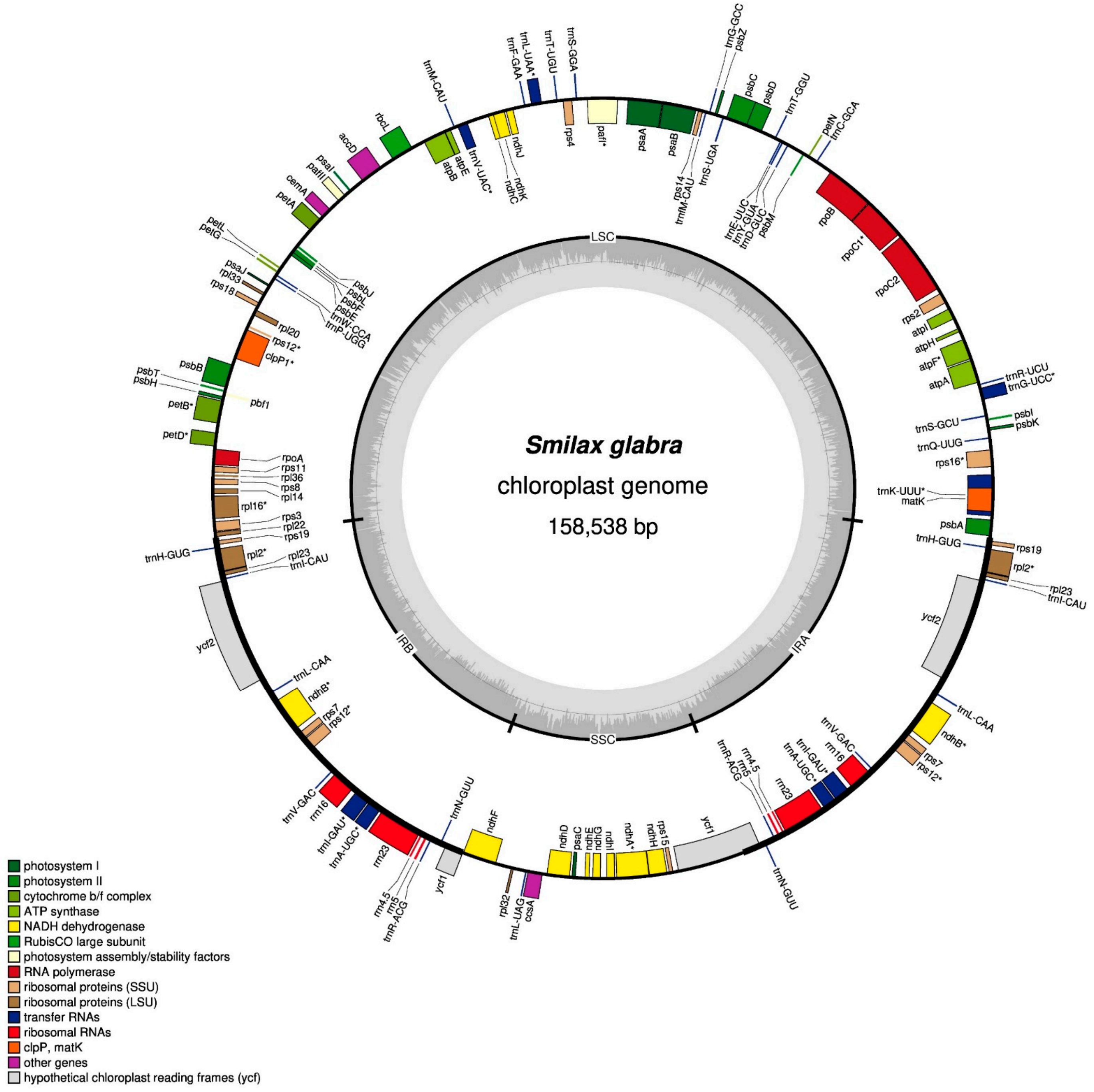

Genes are color-coded based on their functions shown in the key. Genes located outside of the outer circle are transcribed anticlockwise, while those insides are transcribed clockwise. In the inner circle, the gradient in dark grey represents GC content, whereas light grey represents AT content.

Figure S1c. Circular map of the chloroplast genome of *Heterosmilax japonica* (OP076940)

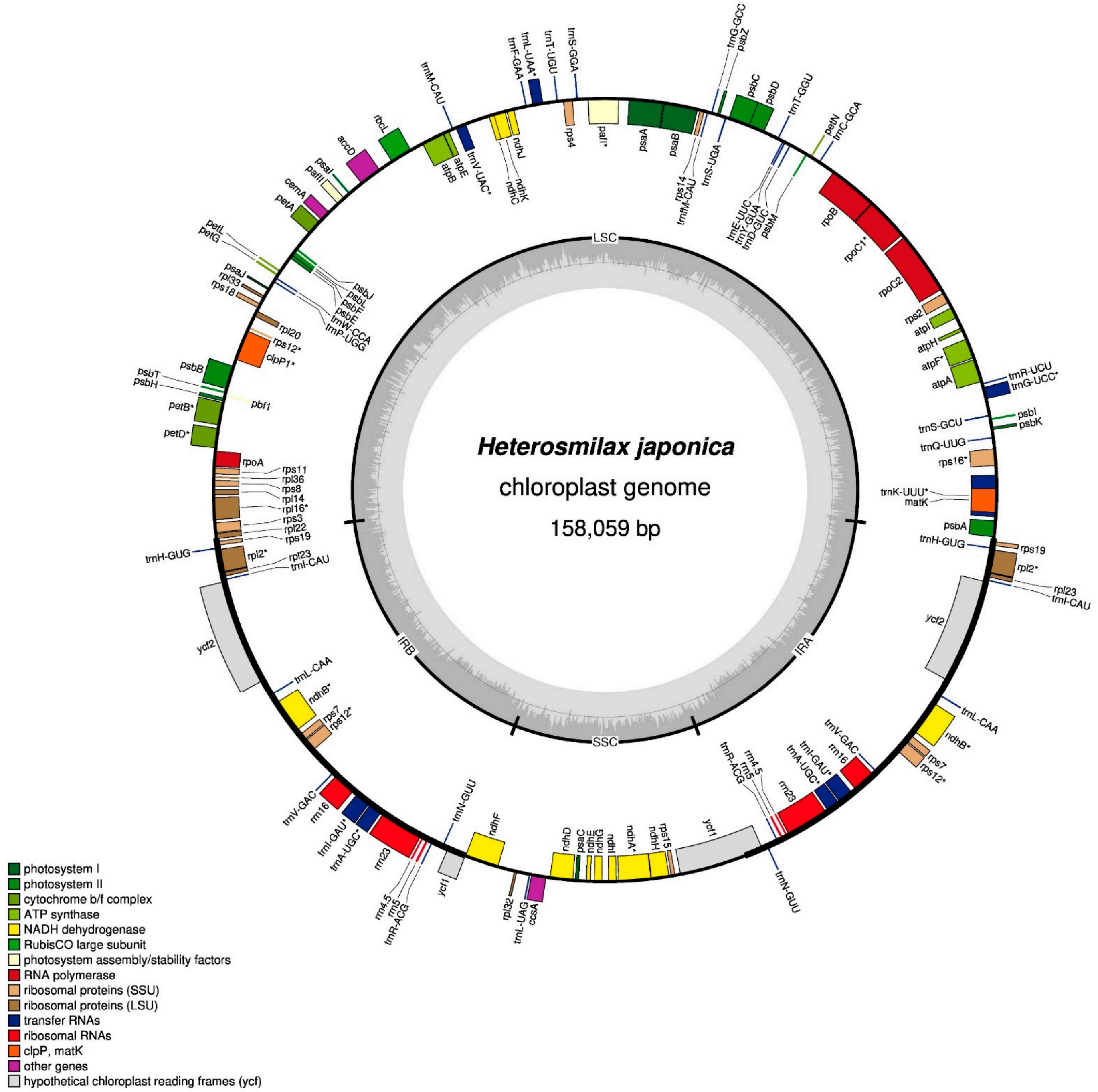

Genes are color-coded based on their functions shown in the key. Genes located outside of the outer circle are transcribed anticlockwise, while those insides are transcribed clockwise. In the inner circle, the gradient in dark grey represents GC content, whereas light grey represents AT content.

Figure S1d. Circular map of the chloroplast genome of *Smilax lanceifolia* var. *opaca* (OP076941)

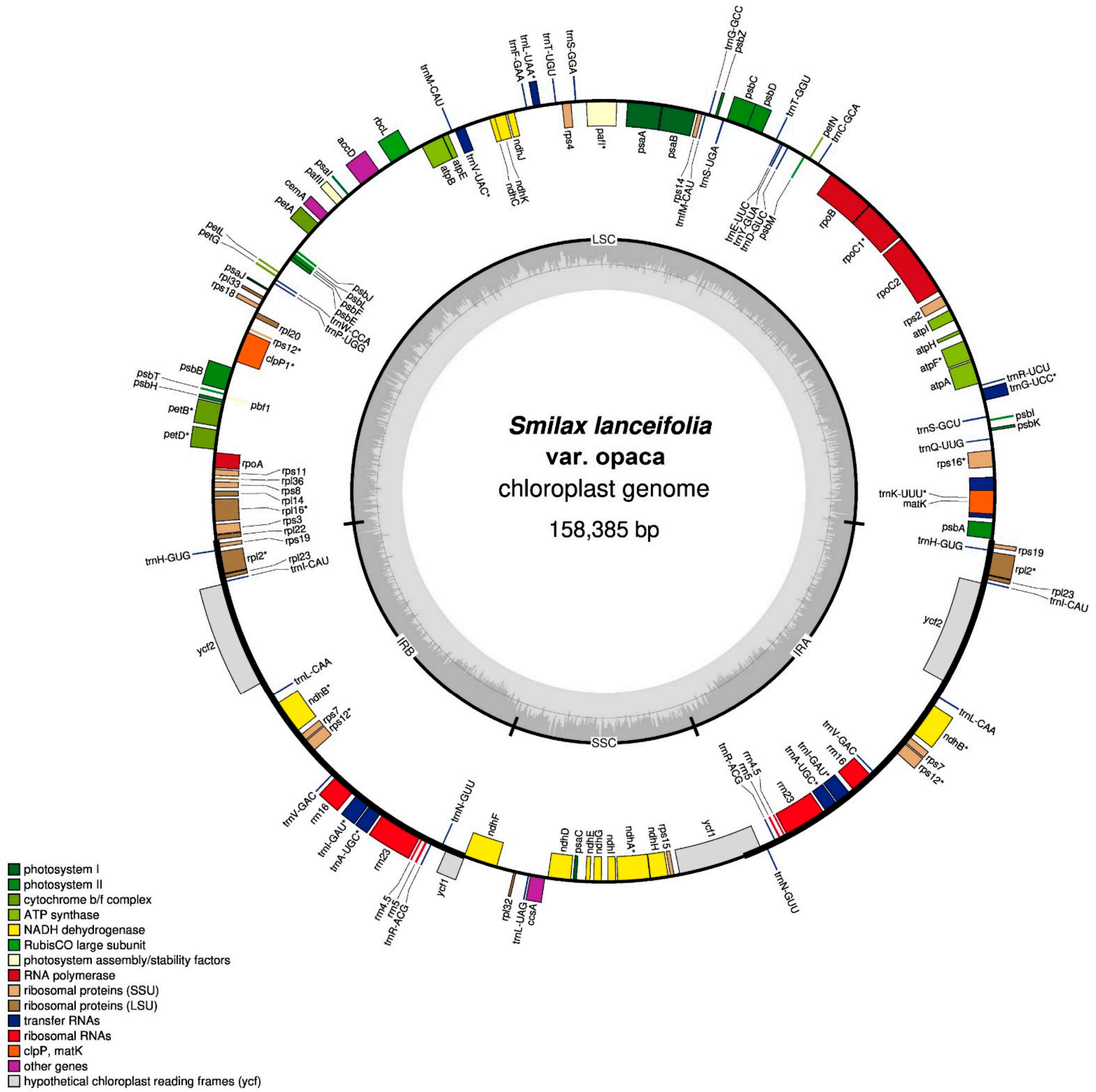

Genes are color-coded based on their functions shown in the key. Genes located outside of the outer circle are transcribed anticlockwise, while those insides are transcribed clockwise. In the inner circle, the gradient in dark grey represents GC content, whereas light grey represents AT content.

Figure S1e. Circular map of the chloroplast genome of *Smilax china* (OP076942)

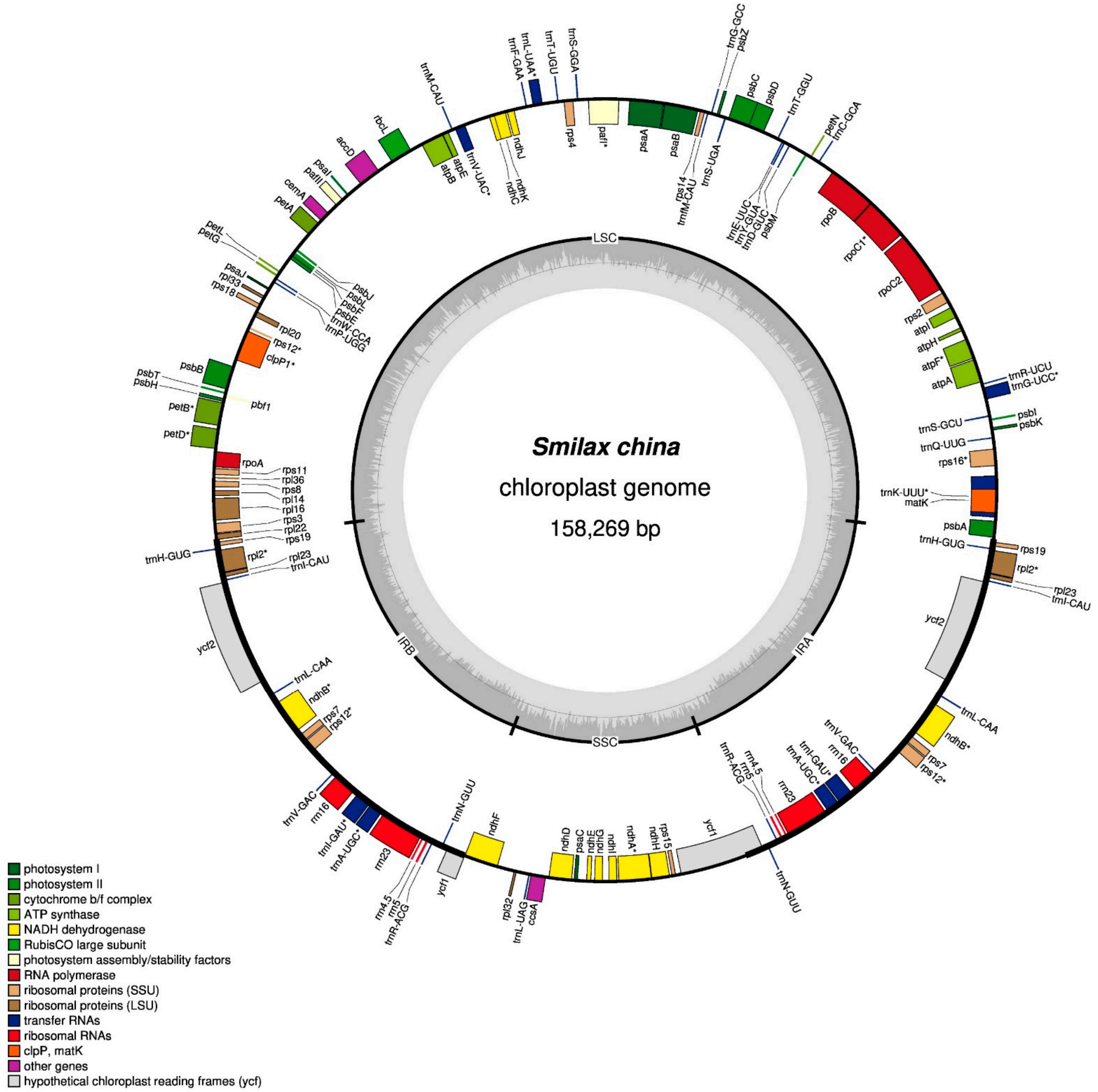

Genes are color-coded based on their functions shown in the key. Genes located outside of the outer circle are transcribed anticlockwise, while those insides are transcribed clockwise. In the inner circle, the gradient in dark grey represents GC content, whereas light grey represents AT content.

Figure S1f. Circular map of the chloroplast genome of *Smilax cocculoides* (OP076943)

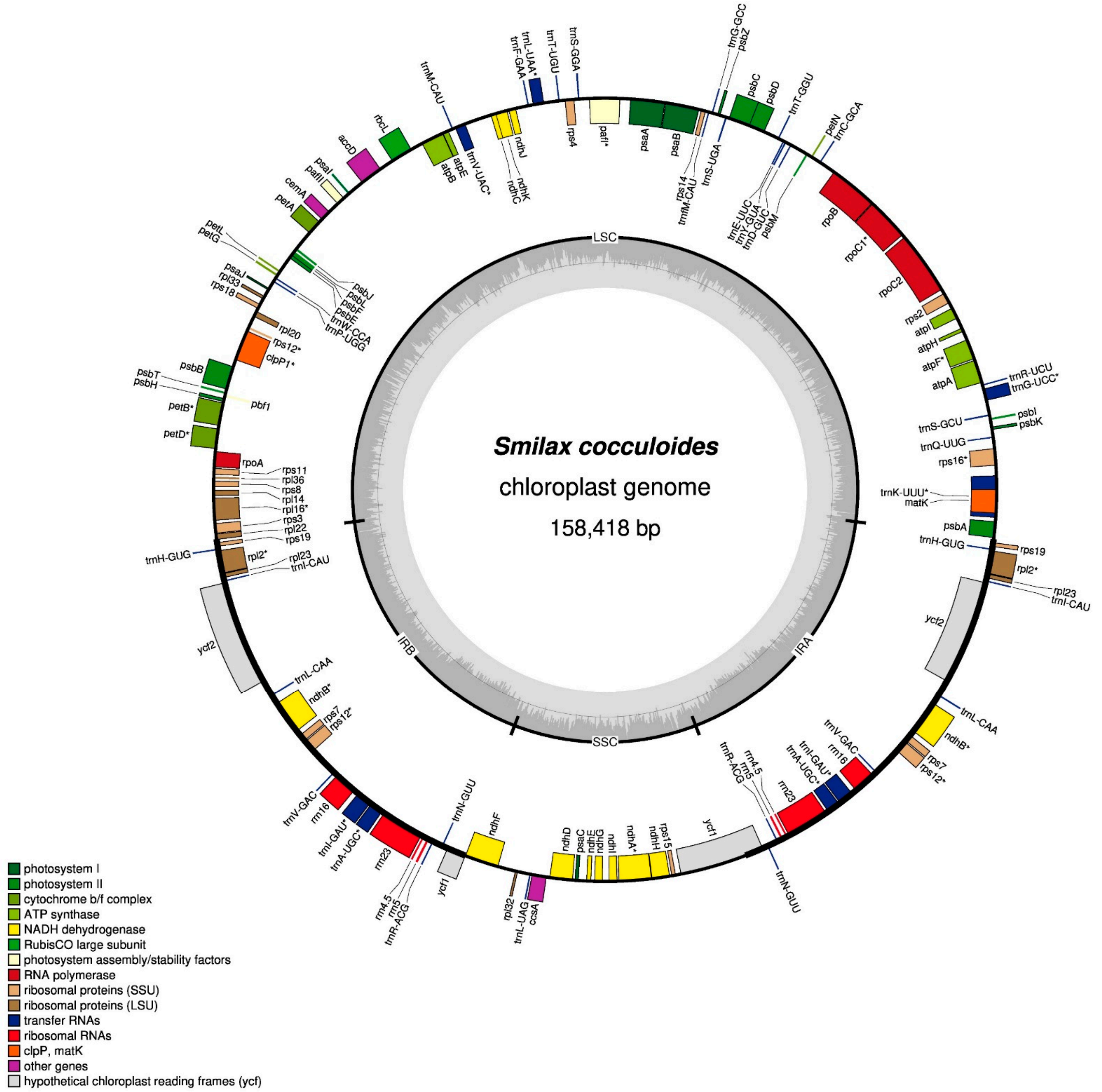

Genes are color-coded based on their functions shown in the key. Genes located outside of the outer circle are transcribed anticlockwise, while those insides are transcribed clockwise. In the inner circle, the gradient in dark grey represents GC content, whereas light grey represents AT content.

Figure S1g. Circular map of the chloroplast genome of *Smilax retroflexa* (OP076944)

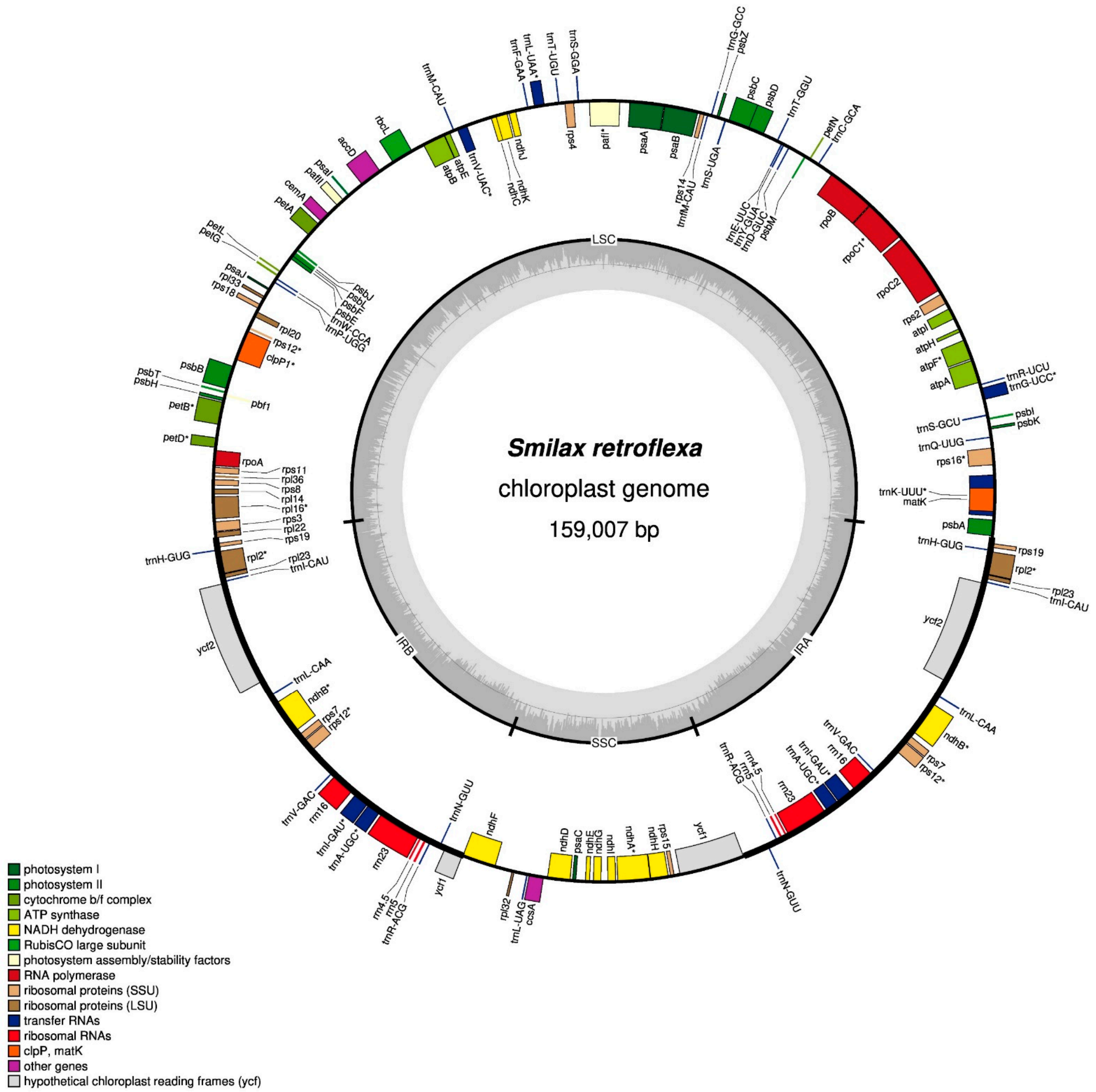

Genes are color-coded based on their functions shown in the key. Genes located outside of the outer circle are transcribed anticlockwise, while those insides are transcribed clockwise. In the inner circle, the gradient in dark grey represents GC content, whereas light grey represents AT content.

Figure S1h. Circular map of the chloroplast genome of *Heterosmilax gaudichaudiana* (OP076945)

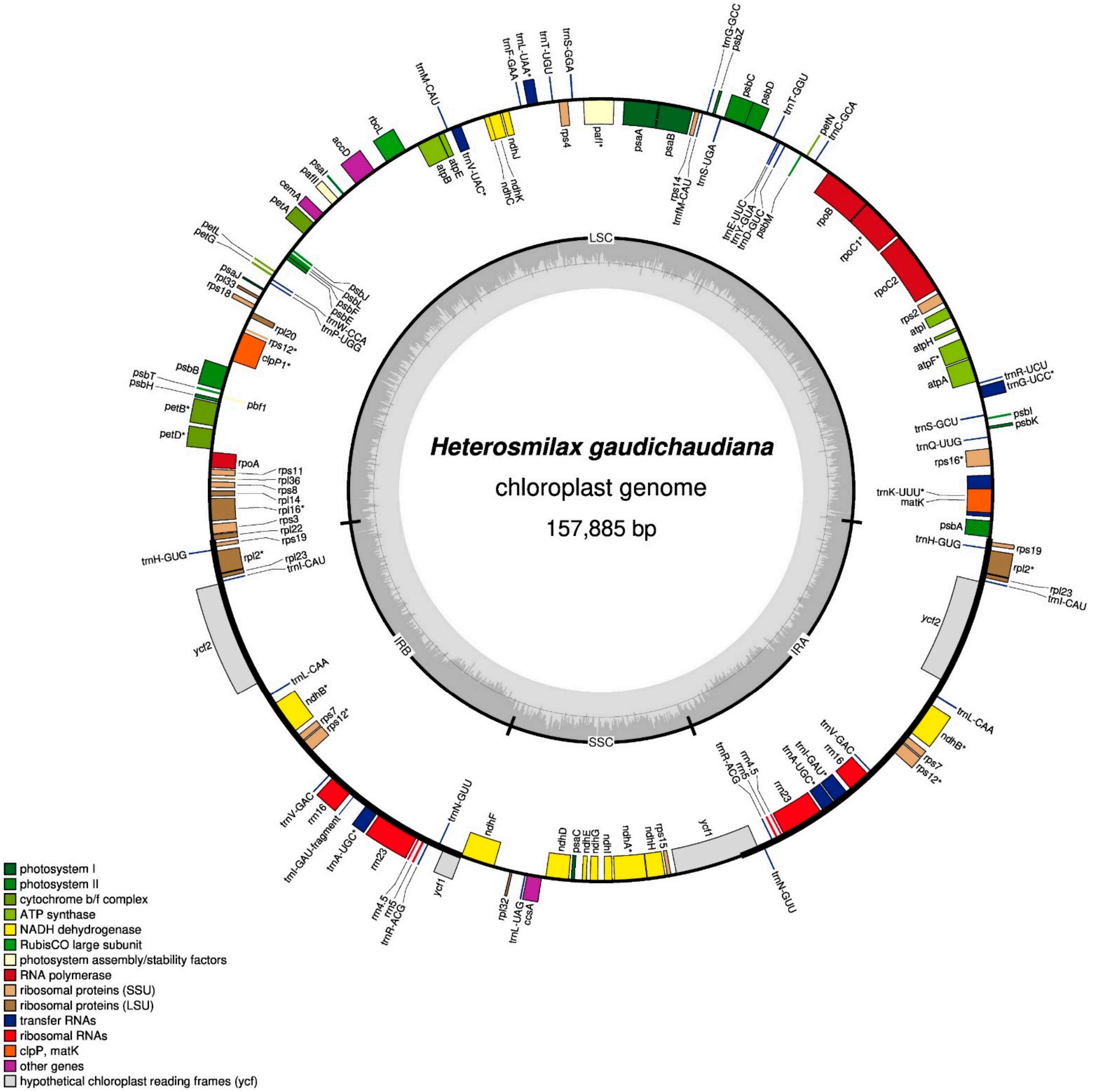

Genes are color-coded based on their functions shown in the key. Genes located outside of the outer circle are transcribed anticlockwise, while those insides are transcribed clockwise. In the inner circle, the gradient in dark grey represents GC content, whereas light grey represents AT content.

Figure S1i. Circular map of the chloroplast genome of *Smilax ocreata* (OP076946)

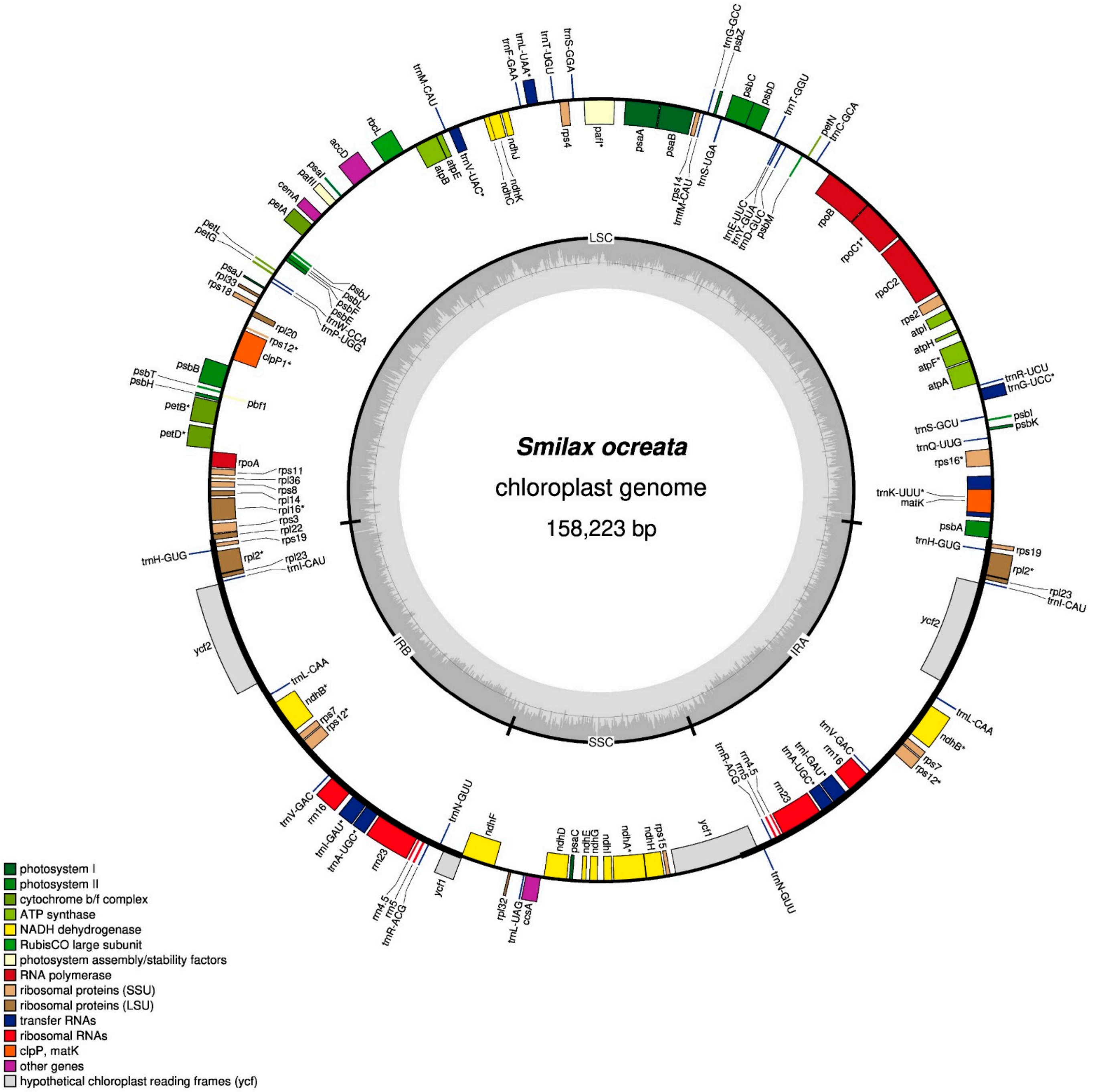

Genes are color-coded based on their functions shown in the key. Genes located outside of the outer circle are transcribed anticlockwise, while those insides are transcribed clockwise. In the inner circle, the gradient in dark grey represents GC content, whereas light grey represents AT content.

Figure S2. Maximum Likelihood tree of Liliales excluding Campynemataceae and Corisaceae accessions

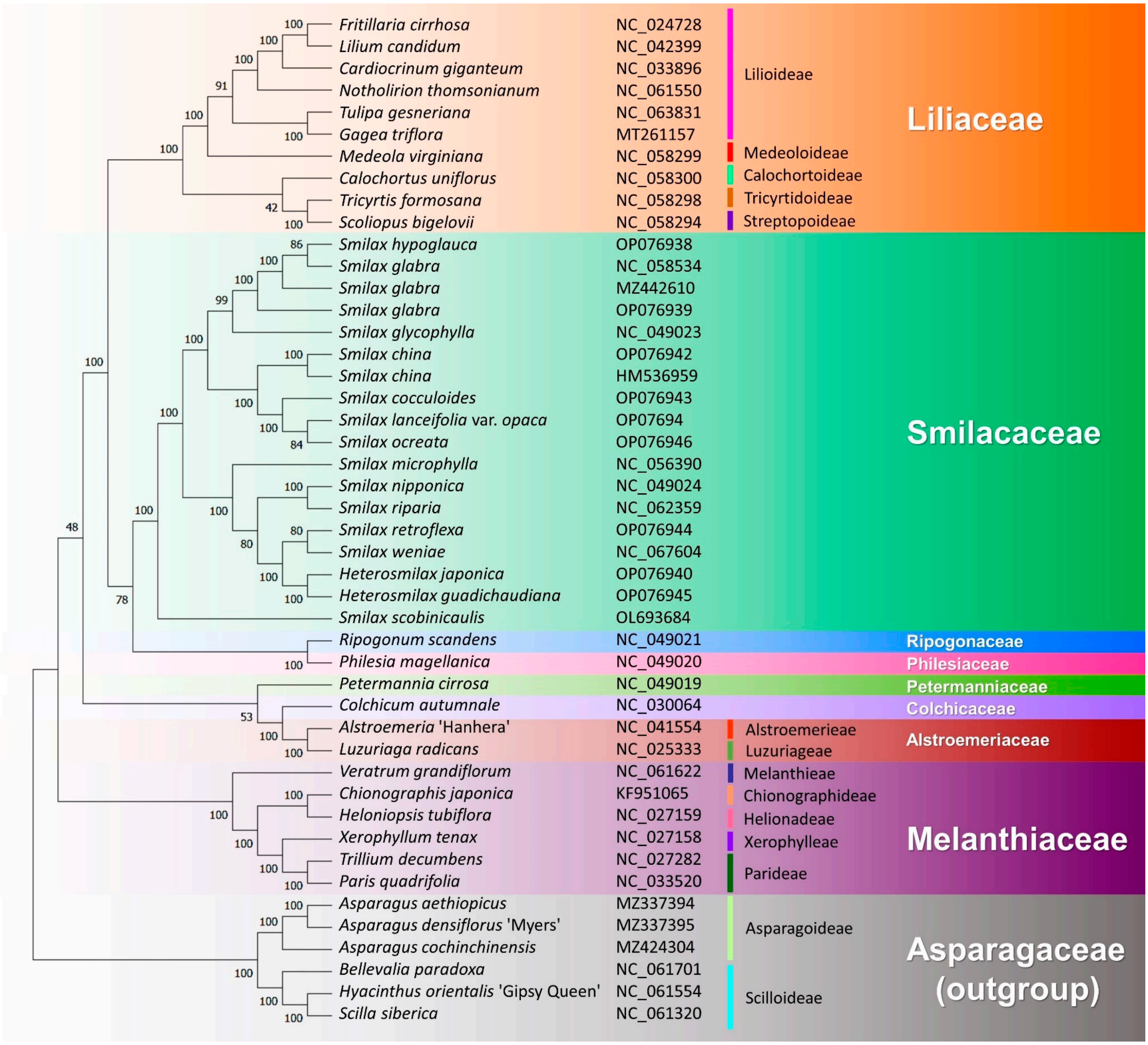

Six cpGenomes of Asparagales-Asparagaceae were selected as the outgroups. Value of Bootstrap Percentage (BP) is shown next to the node of branches. The right side of the accessions is the classified subfamilies (if stated) and families *sensu* APG IV for each accession.

Figure S3. Bayesian Inference tree of Liliales excluding Campynemataceae and Corisaceae accessions

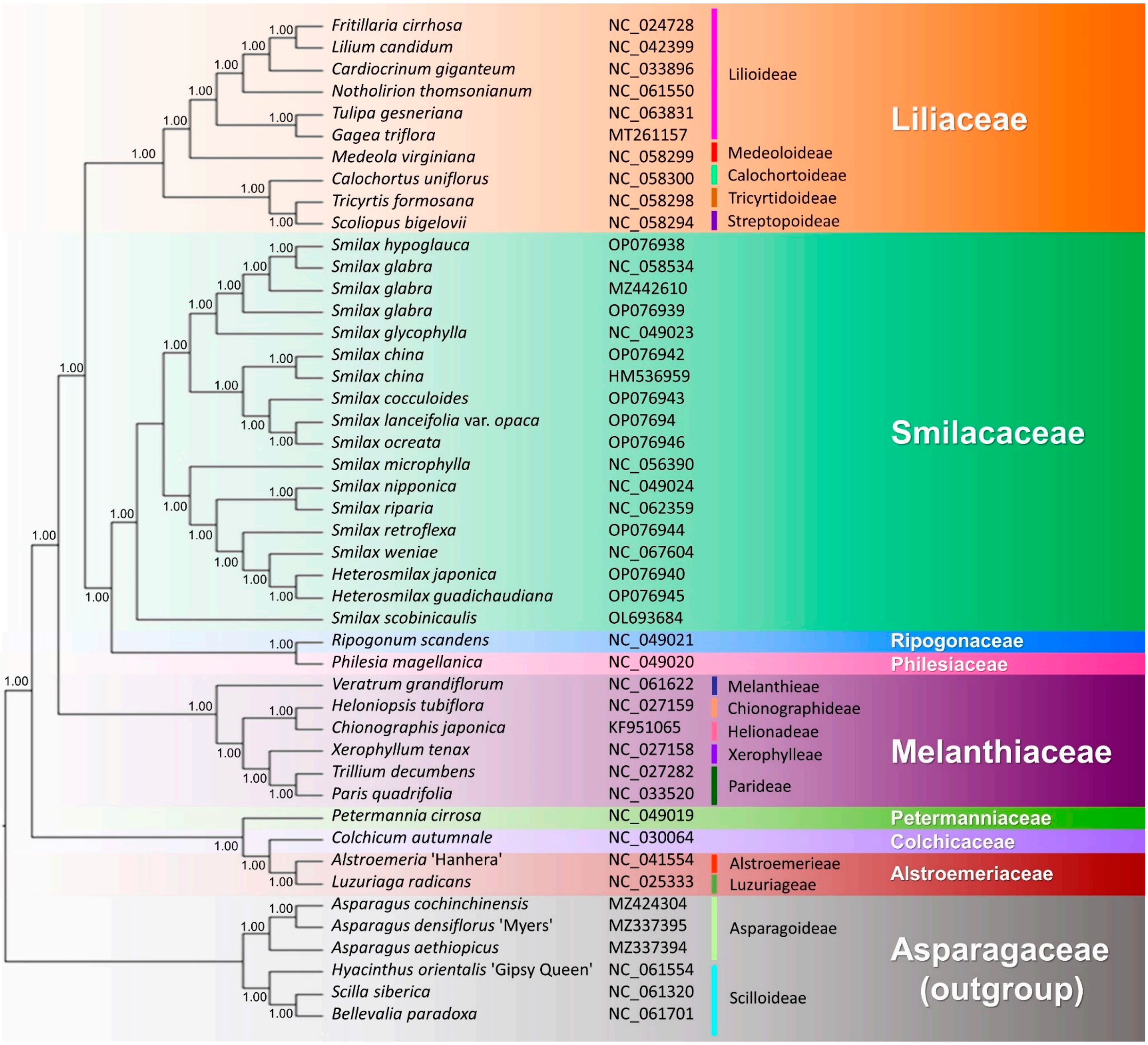

Six cpGenomes of Asparagales-Asparagaceae were selected as the outgroups. Value of Posterior Probability (PP) is shown next to the node of branches. The right side of the accessions is the classified subfamilies (if stated) and families sensu APG IV for each accession.

Figure S4a. Voucher specimen of the *Smilax hypoglauca* (T. Y. Siu 426, CUHK05490)

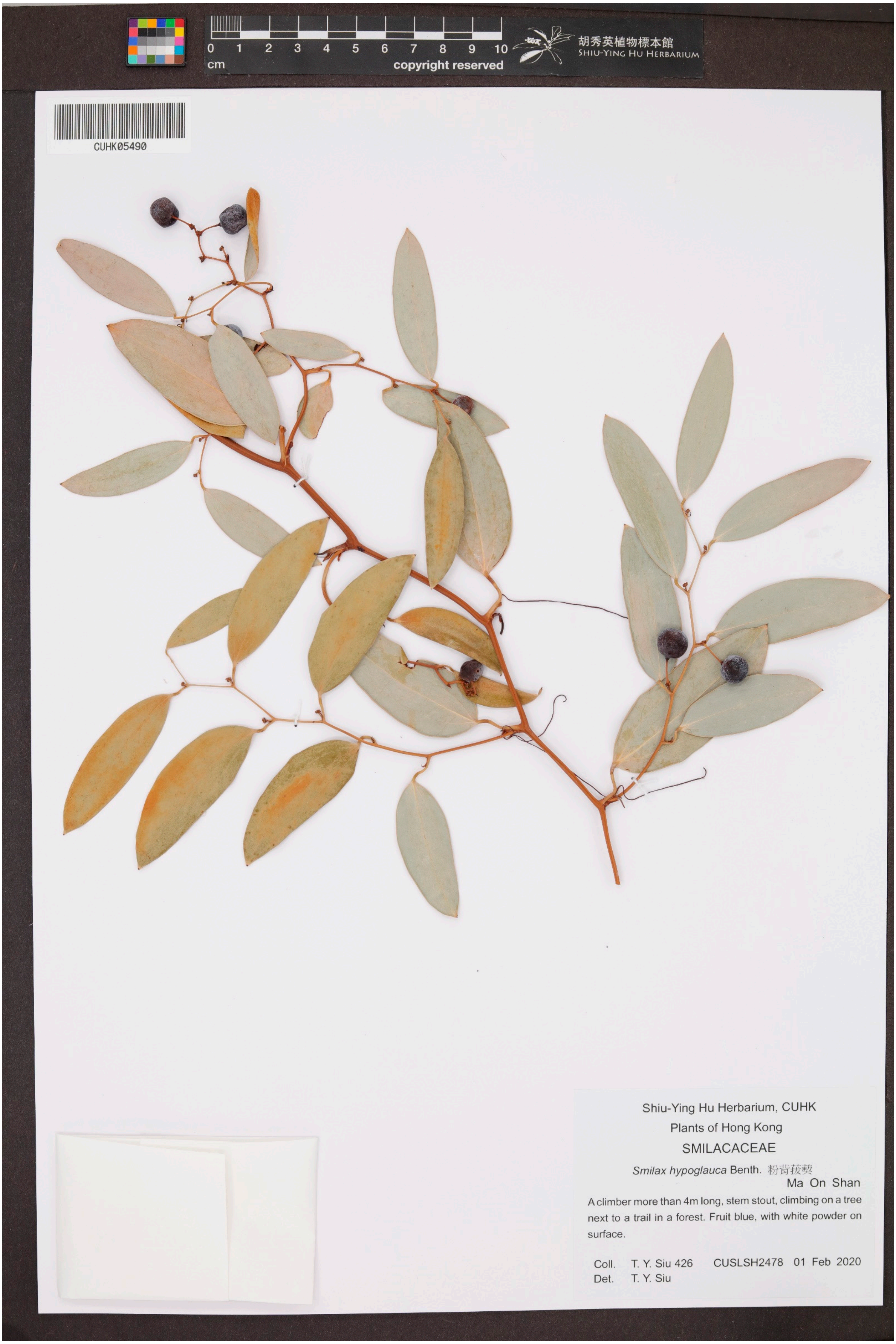

Figure S4b. Voucher specimen of the *Smilax glabra* (T. Y. Siu 658, CUHK05496)

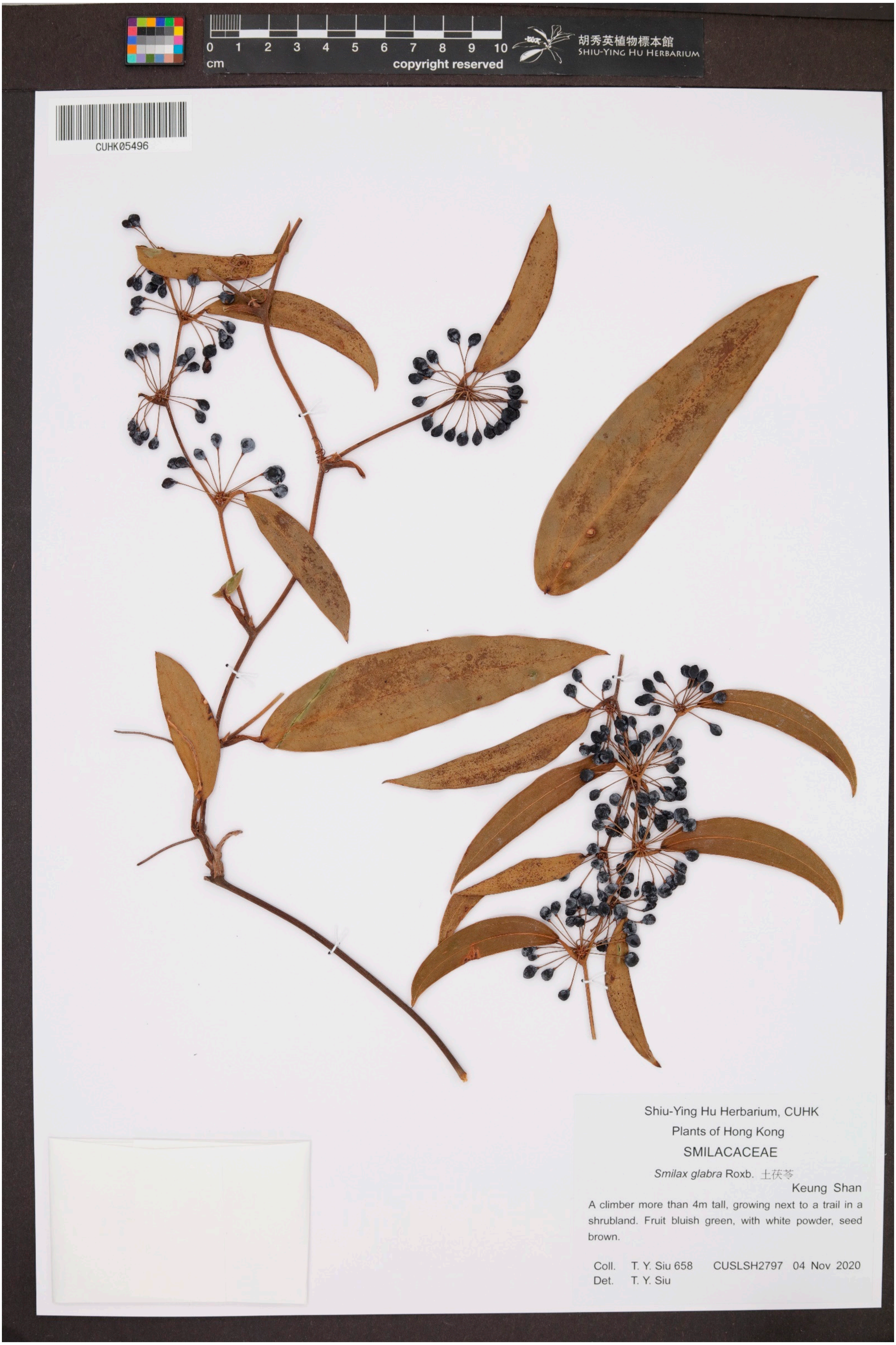

Figure S4c. Voucher specimen of the *Heterosmilax japonica* (T. Y. Siu 812, CUHK06147)

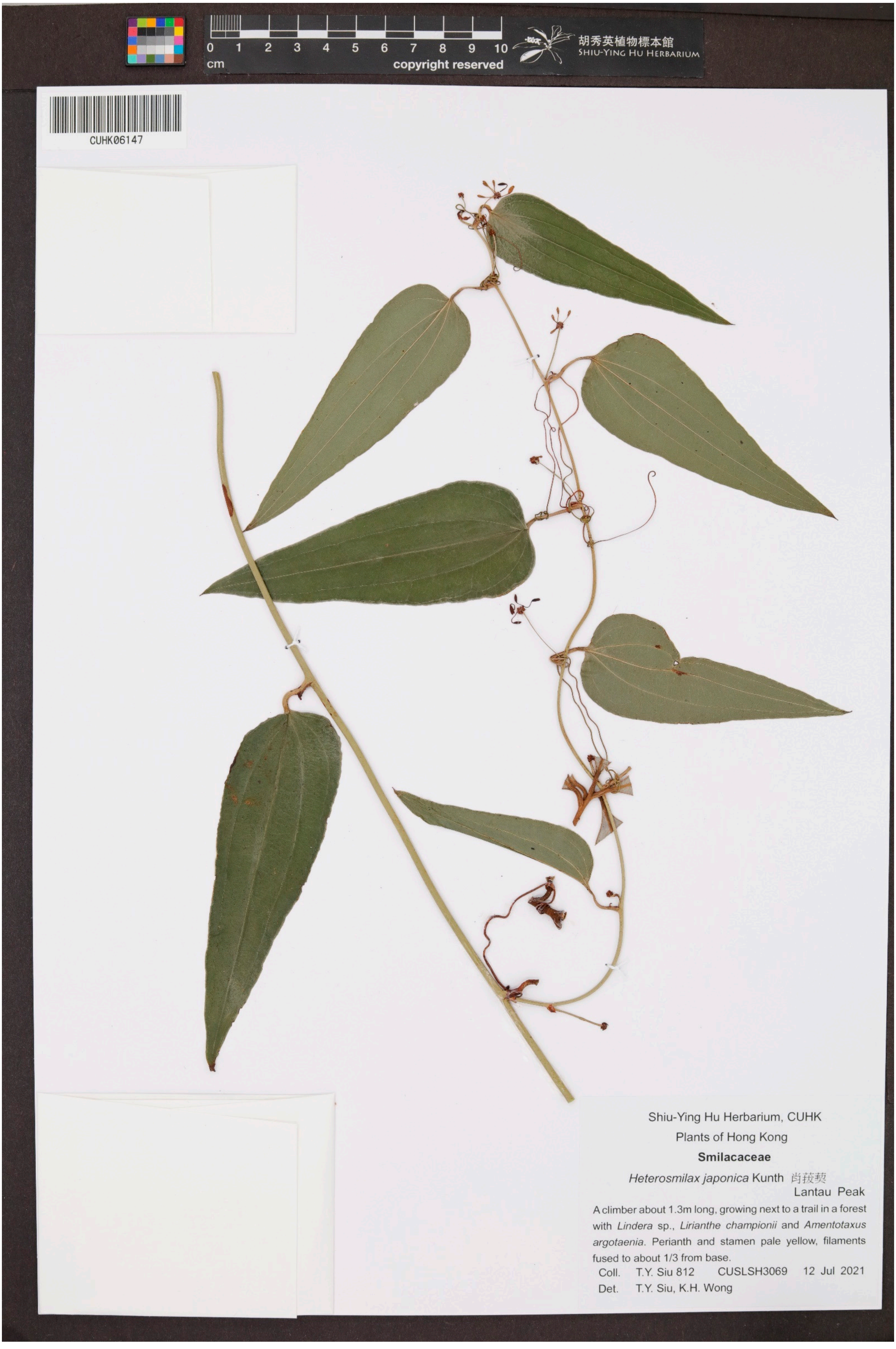

Figure S4d. Voucher specimen of the *Smilax lanceifolia* var. *opaca* (K. H. Wong 150, CUHK06148)

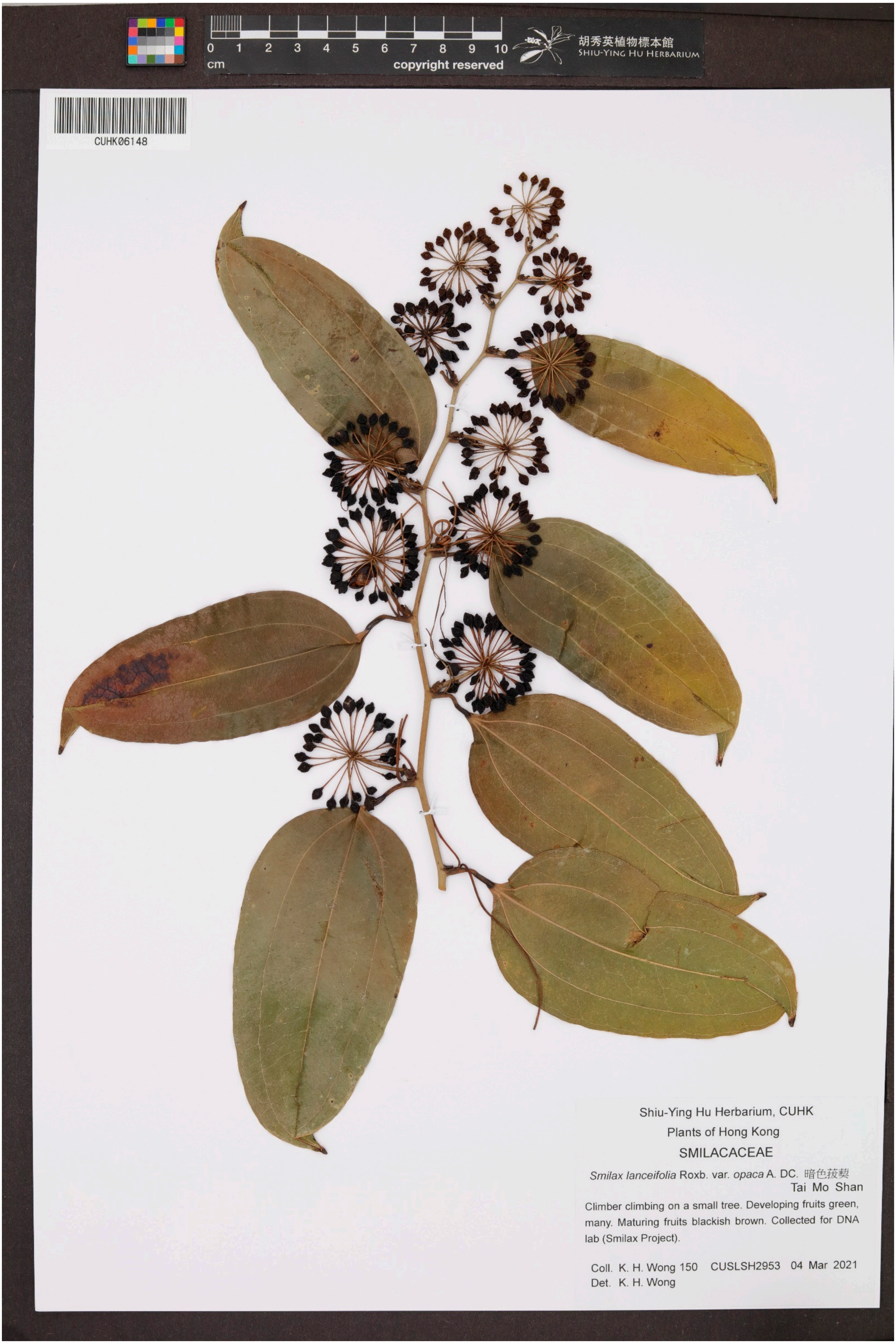

Figure S4e. Voucher specimen of the *Smilax lanceifolia* var. *opaca* (K. H. Wong 150, CUHK06149)

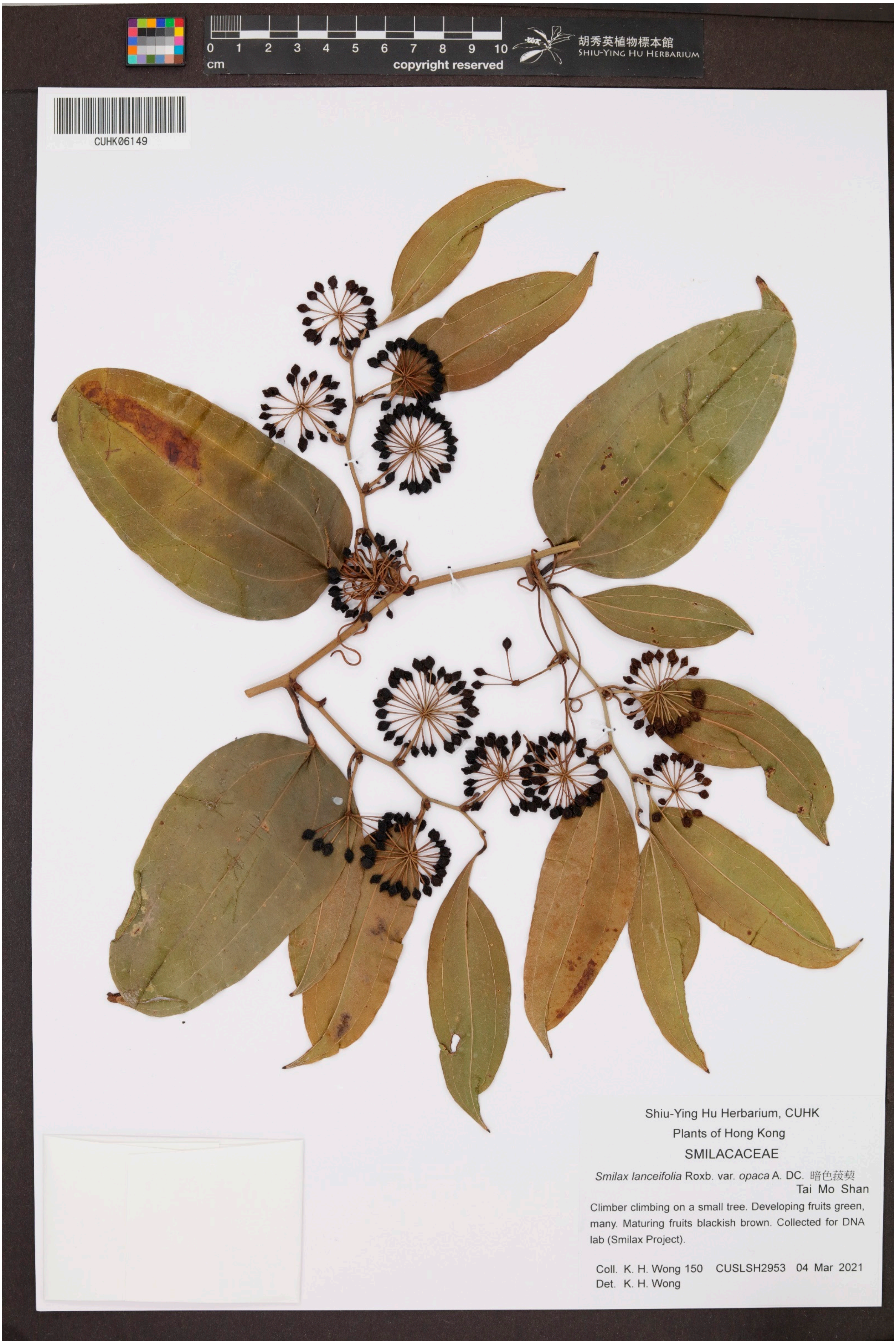

Figure S4f. Voucher specimen of the *Smilax lanceifolia* var. *opaca* (K. H. Wong 150, CUHK06150)

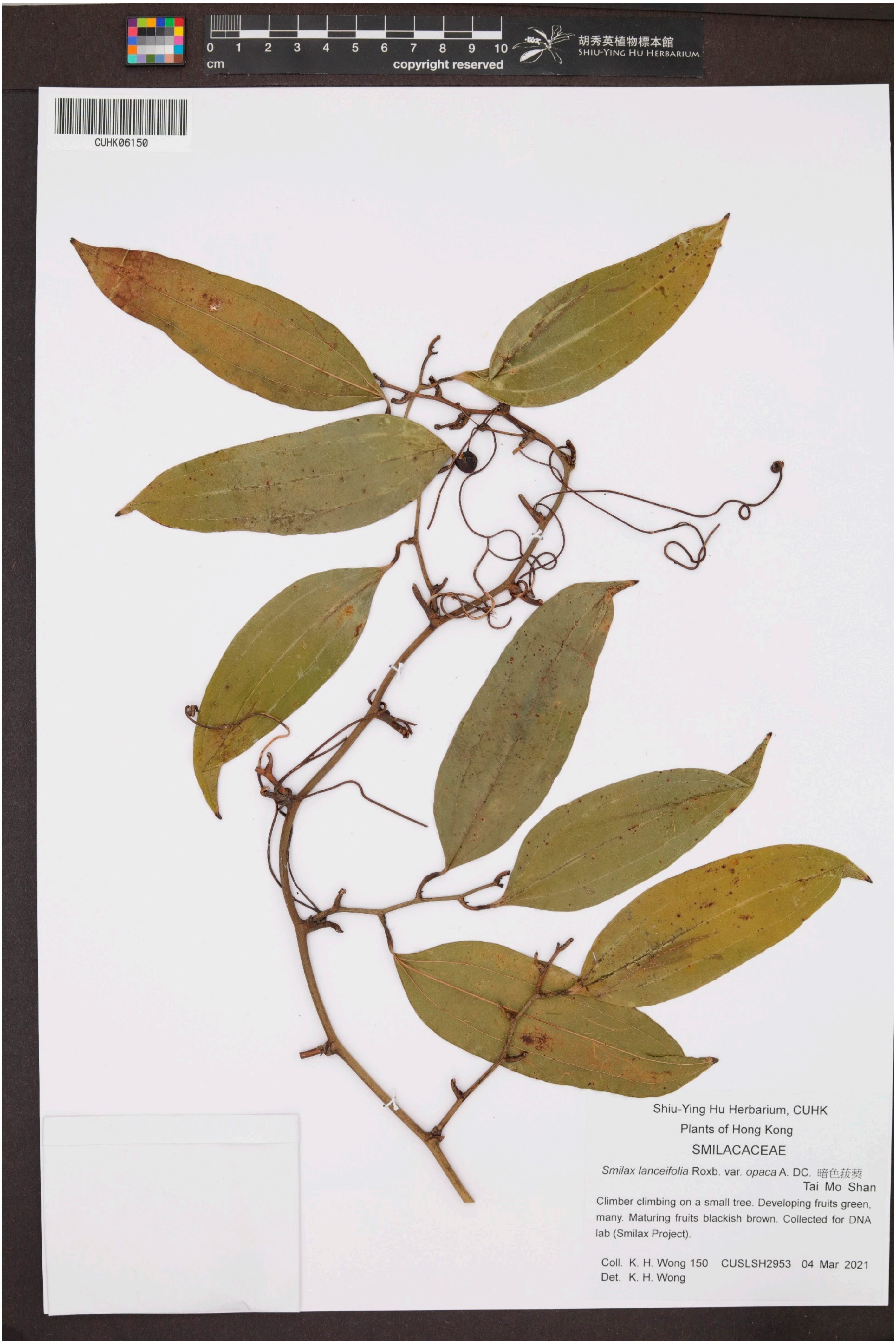

Figure S4g. Voucher specimen of the *Smilax china* (K. H. Wong 154, CUHK06151)

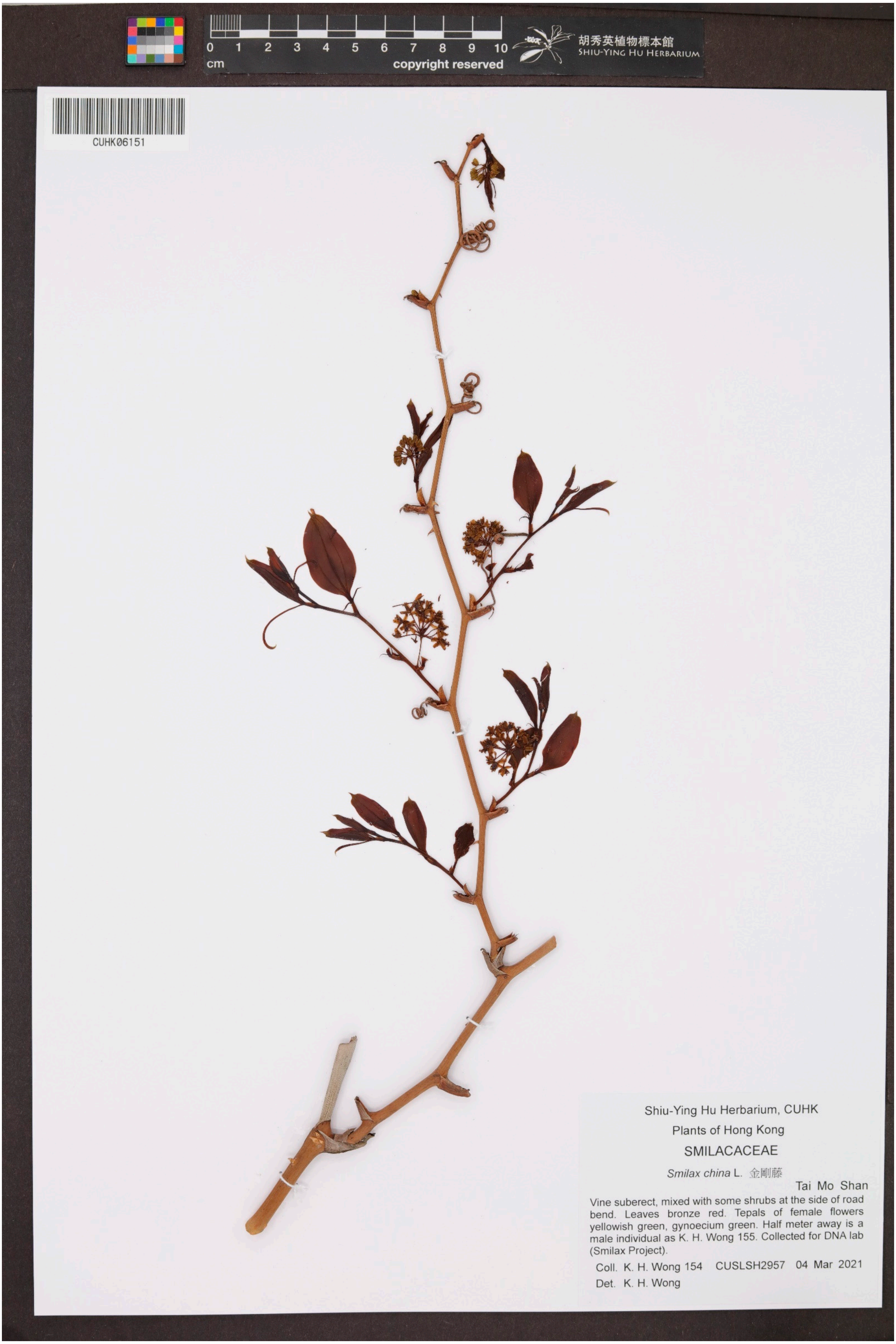

Figure S4h. Voucher specimen of the *Smilax china* (K. H. Wong 154, CUHK06152)

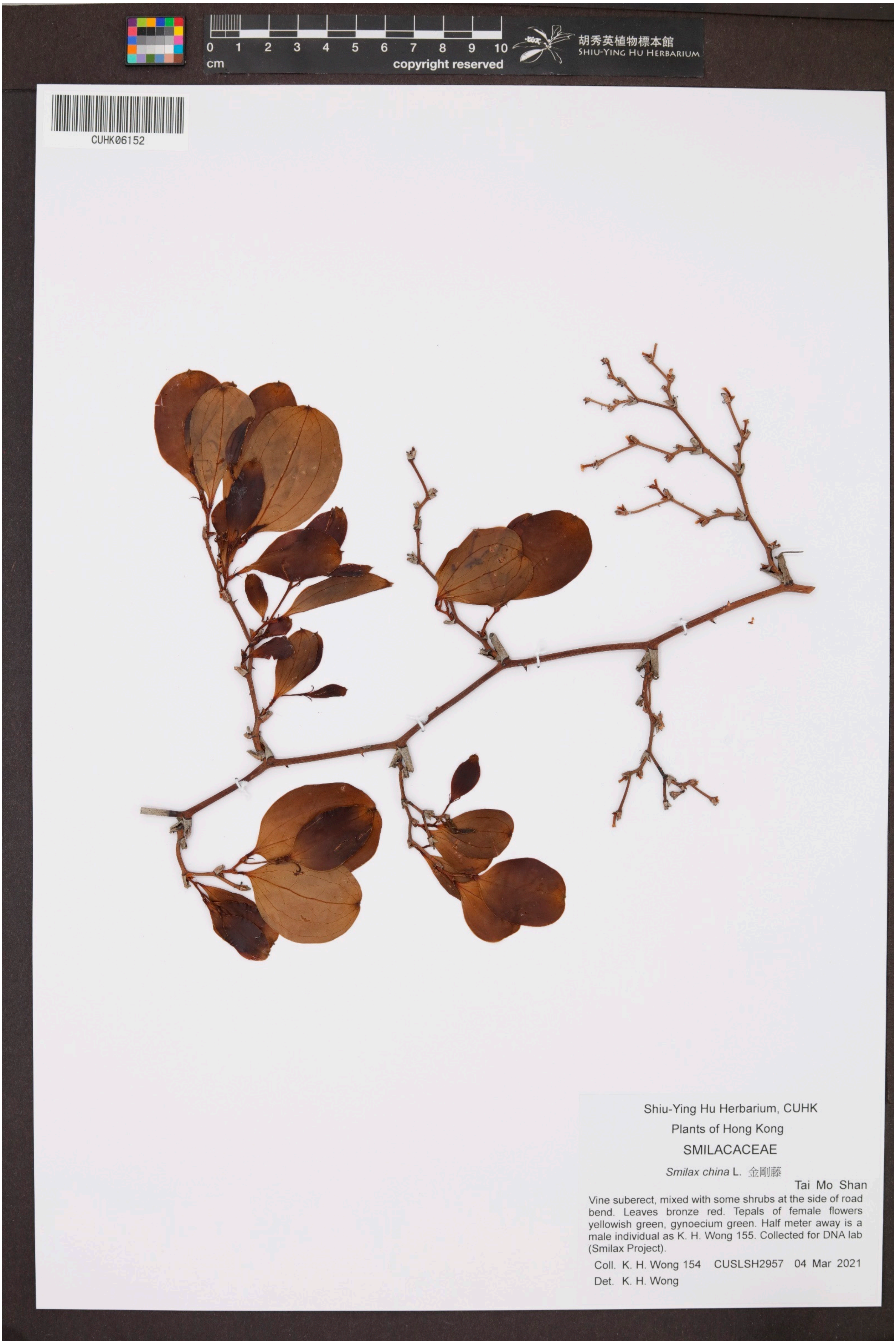

Figure S4i. Voucher specimen of the *Smilax cocculoides* (K. H. Wong 160, CUHK06153)

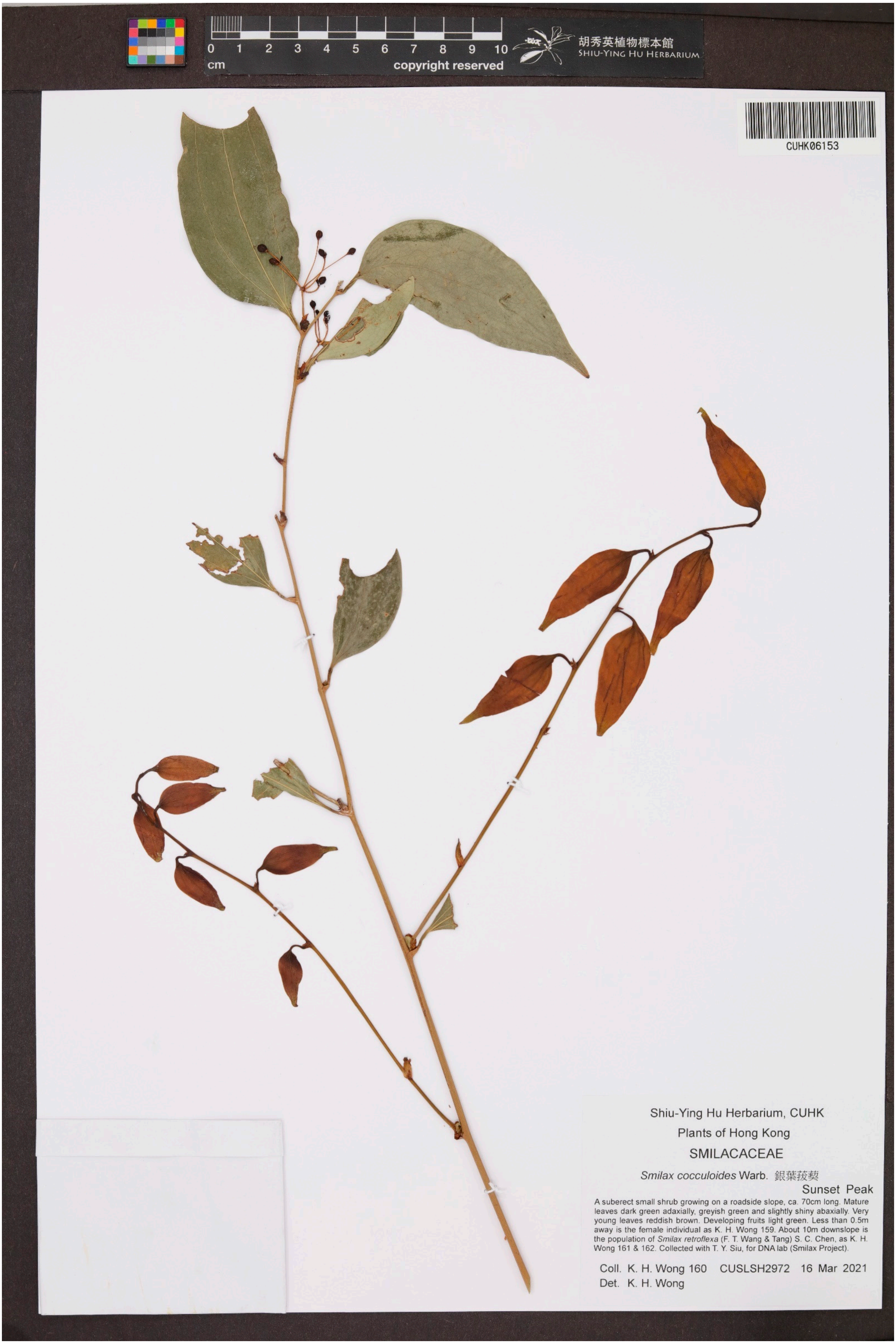

Figure S4j. Voucher specimen of the *Smilax cocculoides* (K. H. Wong 160, CUHK06154)

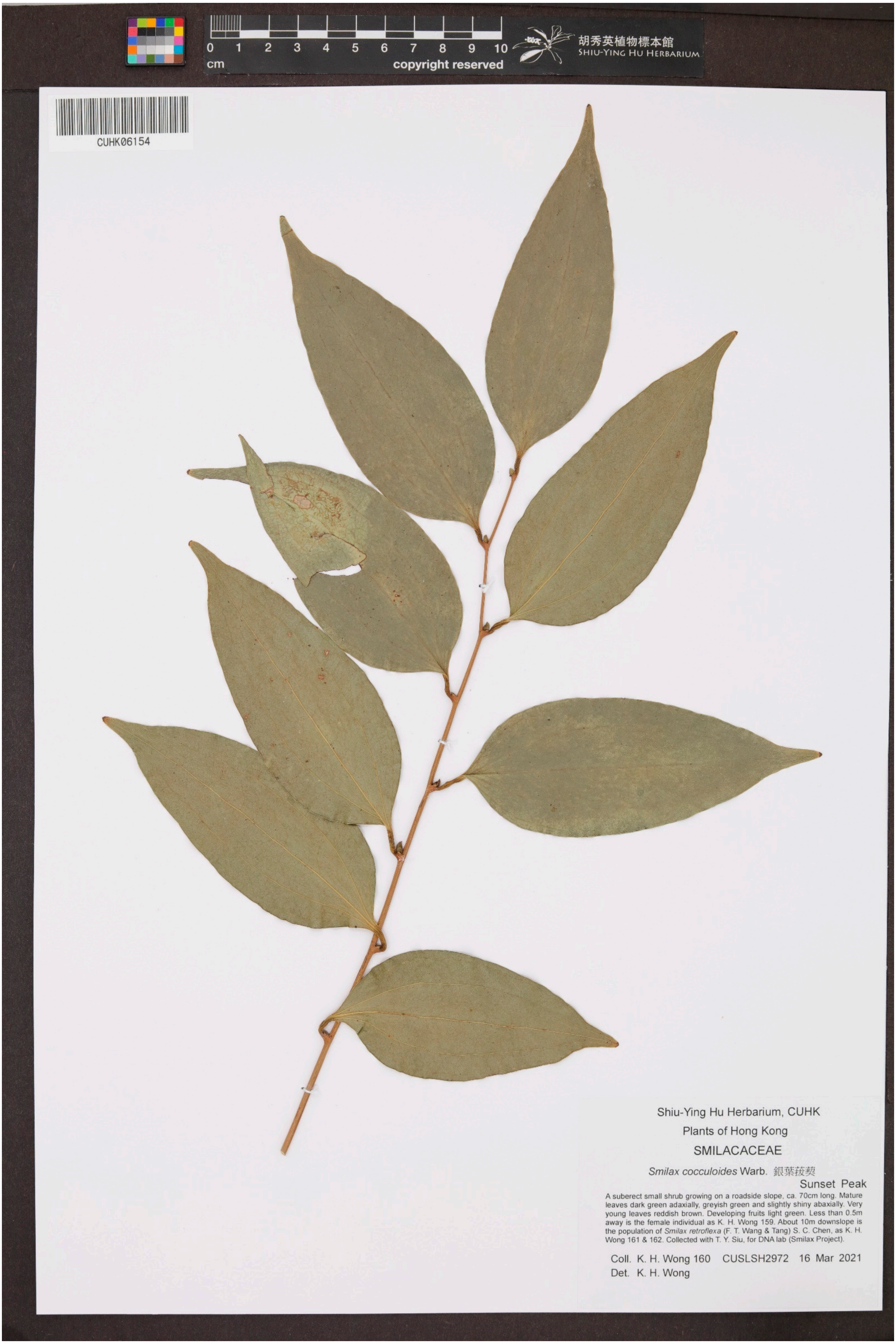

Figure S4k. Voucher specimen of the *Smilax retroflexa* (K. H. Wong 162, CUHK06155)

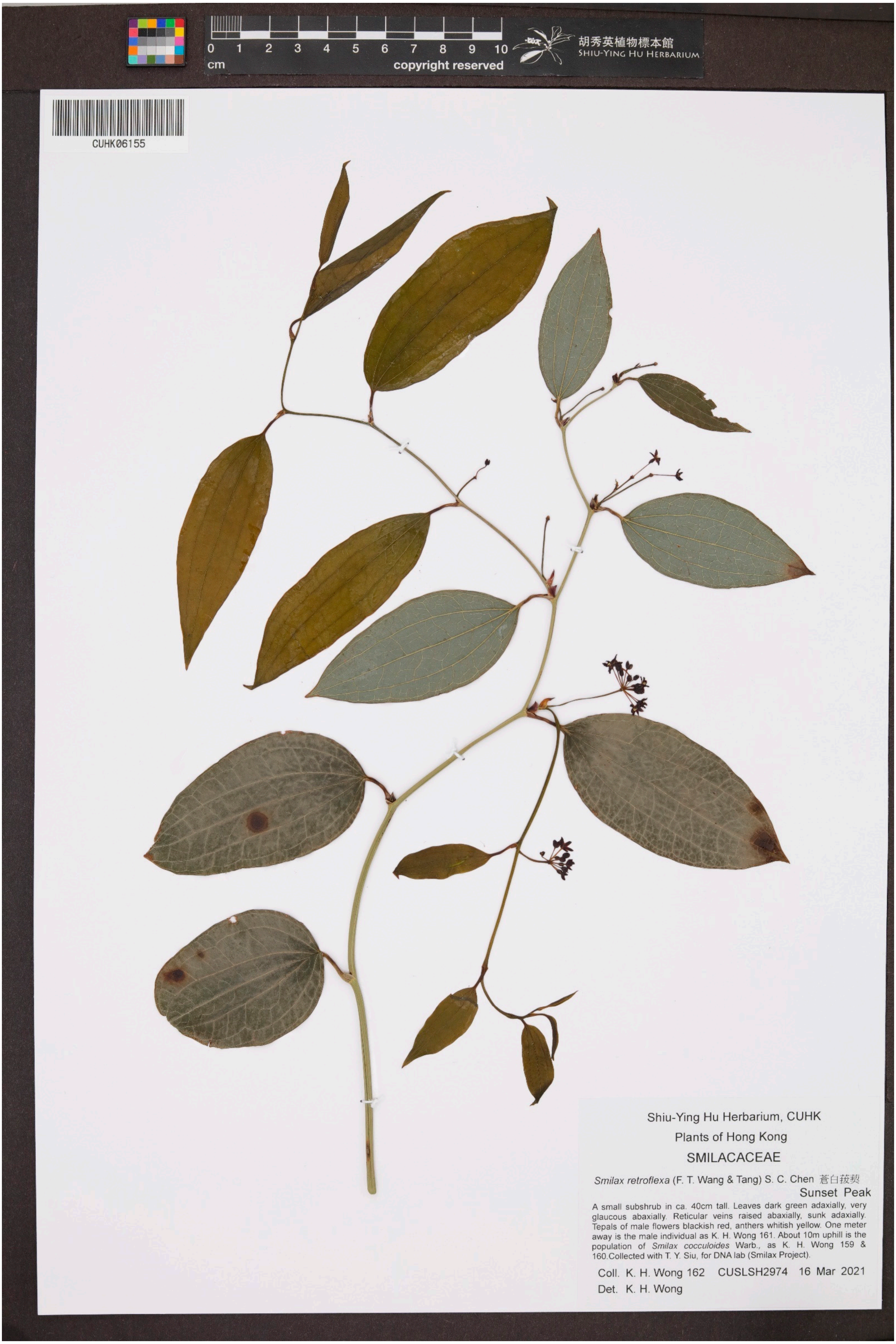

Figure S4m. Voucher specimen of the *Smilax retroflexa* (K. H. Wong 162, CUHK06156)

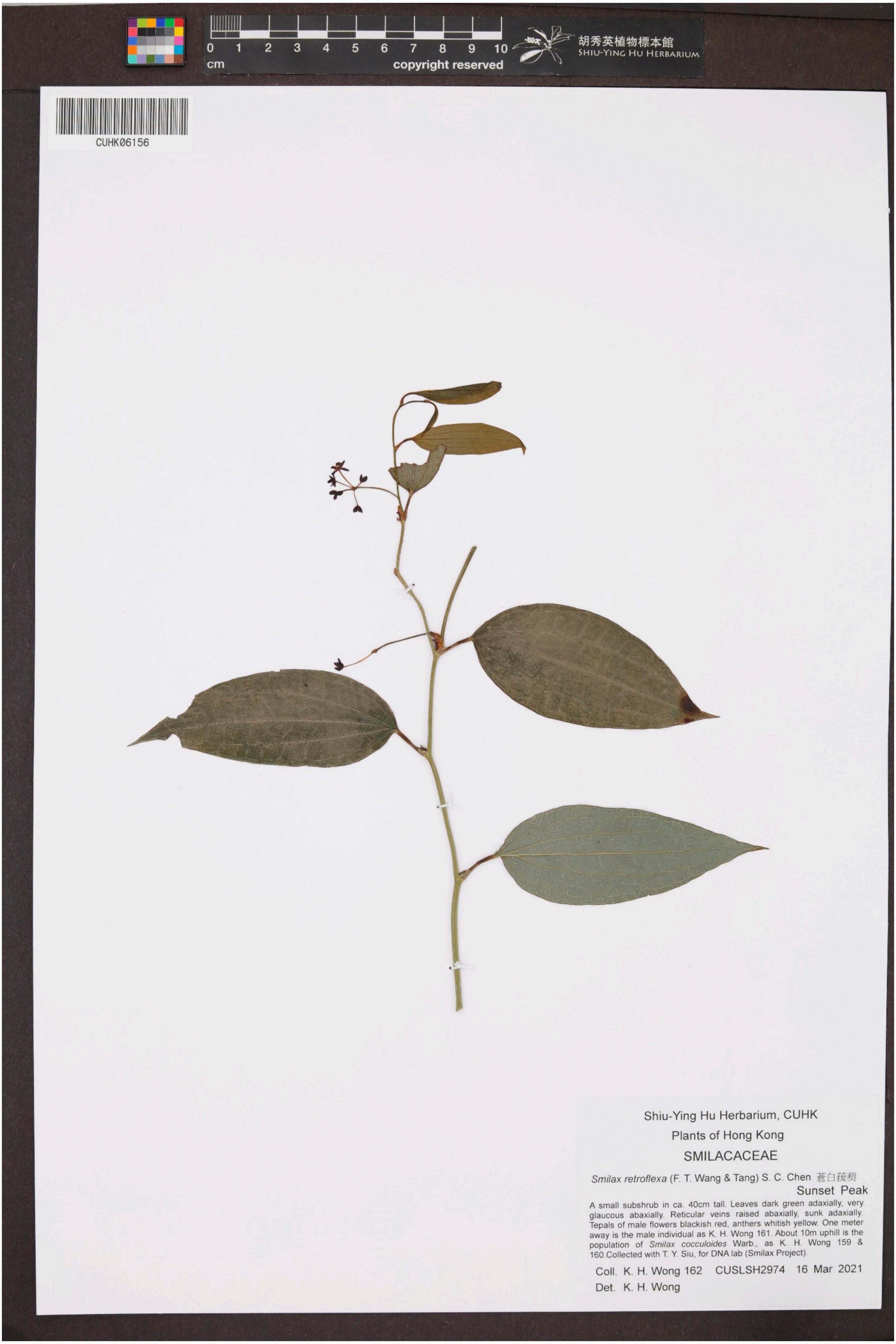

Figure S4n. Voucher specimen of the *Smilax retroflexa* (K. H. Wong 162, CUHK06157)

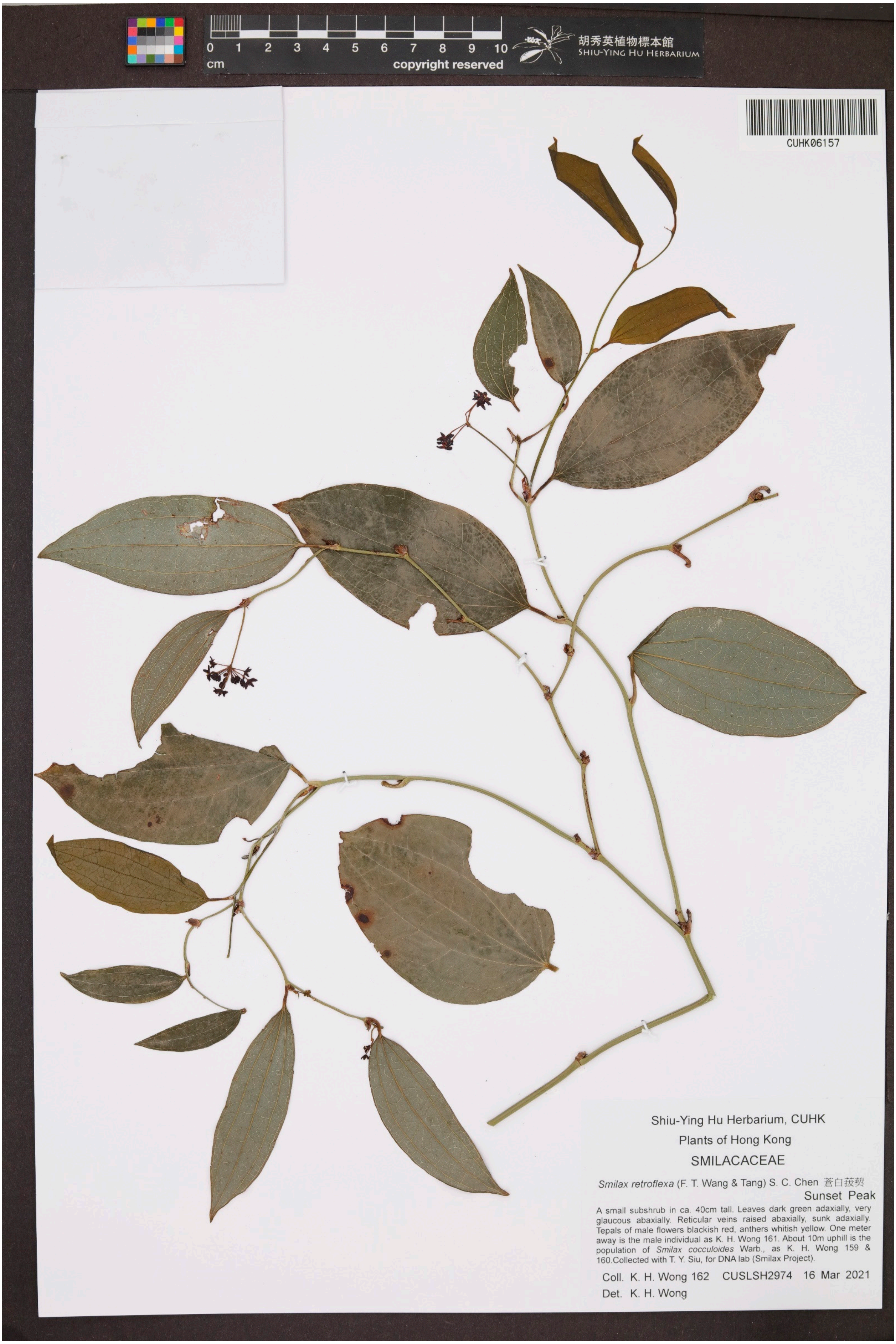

Figure S4o. Voucher specimen of the *Heterosmilax gaudichaudiana* (K. H. Wong 169, CUHK06158)

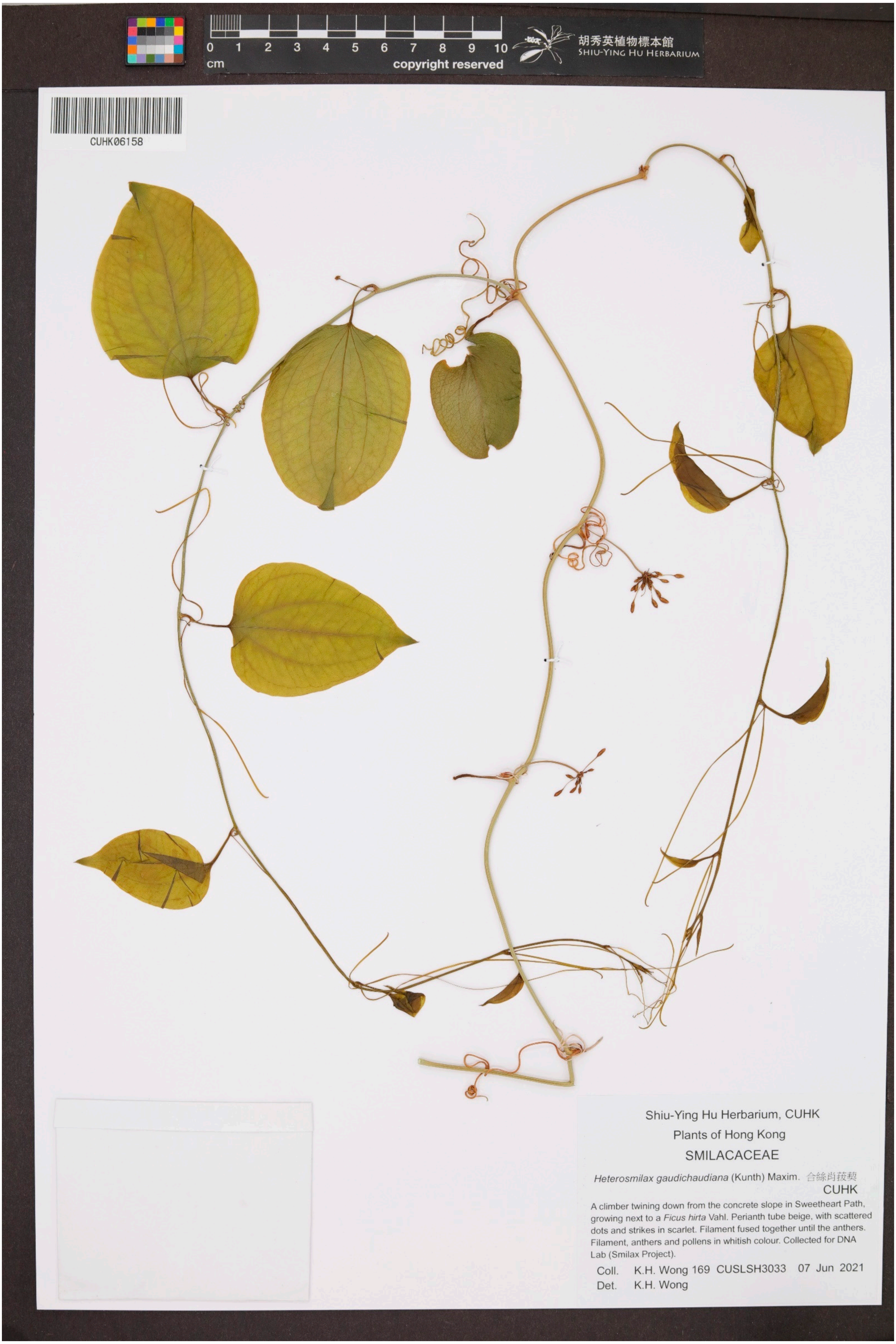

Figure S4p. Voucher specimen of the *Smilax ocreata* (K. H. Wong 176, CUHK06159)

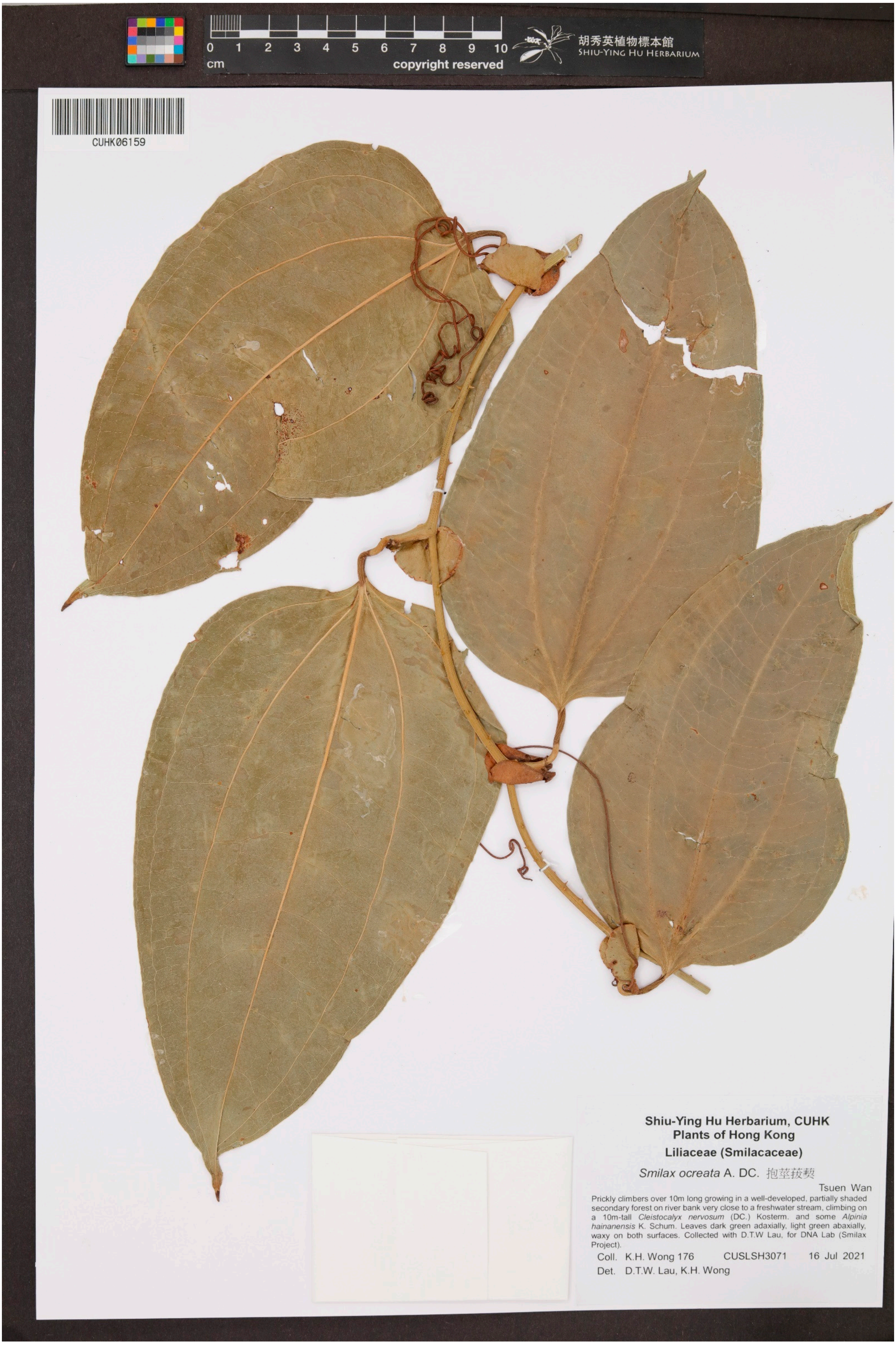

Figure S4q. Voucher specimen of the *Smilax ocreata* (K. H. Wong 176, CUHK06160)

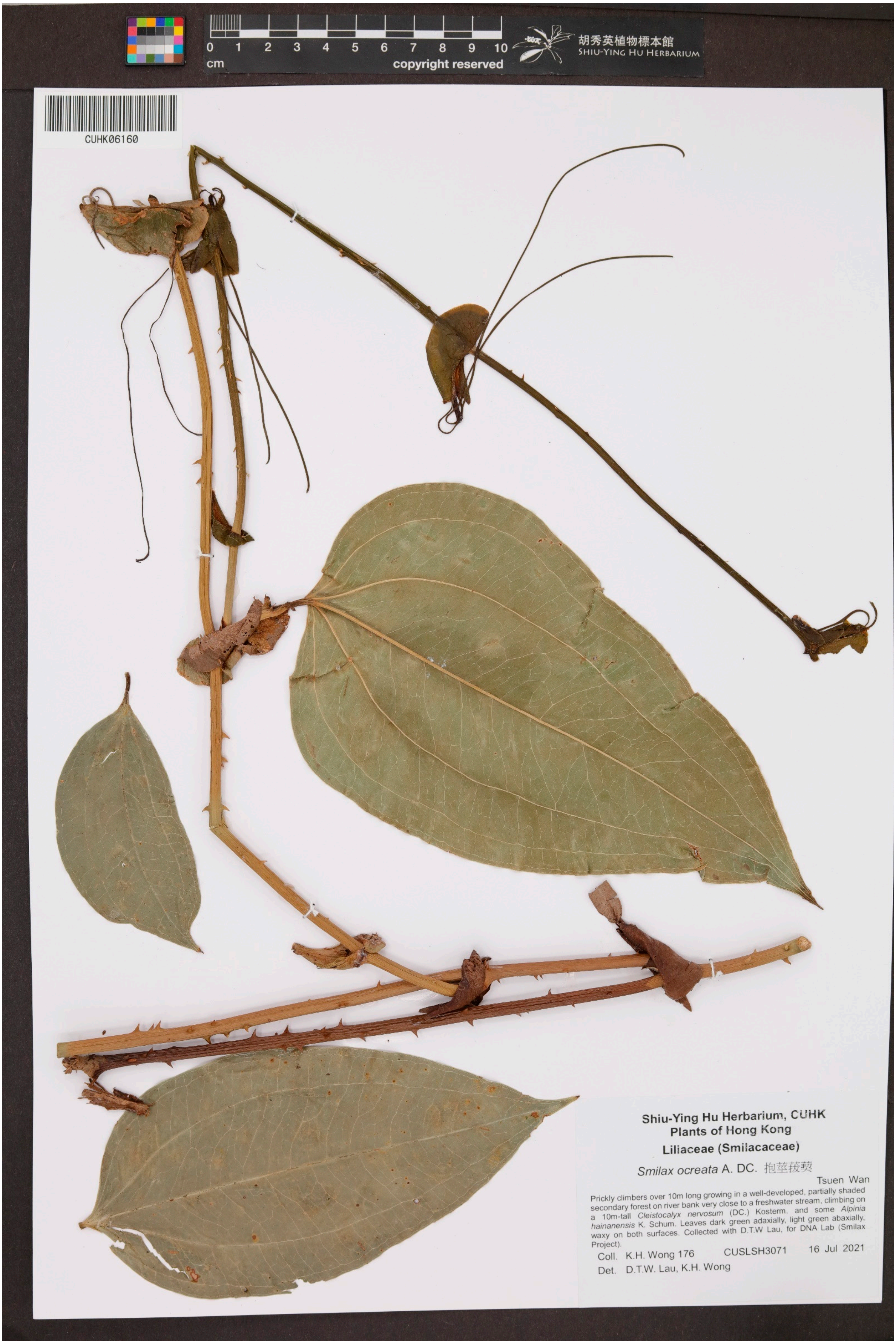

Figure S4r. Voucher specimen of the *Smilax ocreata* (K. H. Wong 176, CUHK06161)

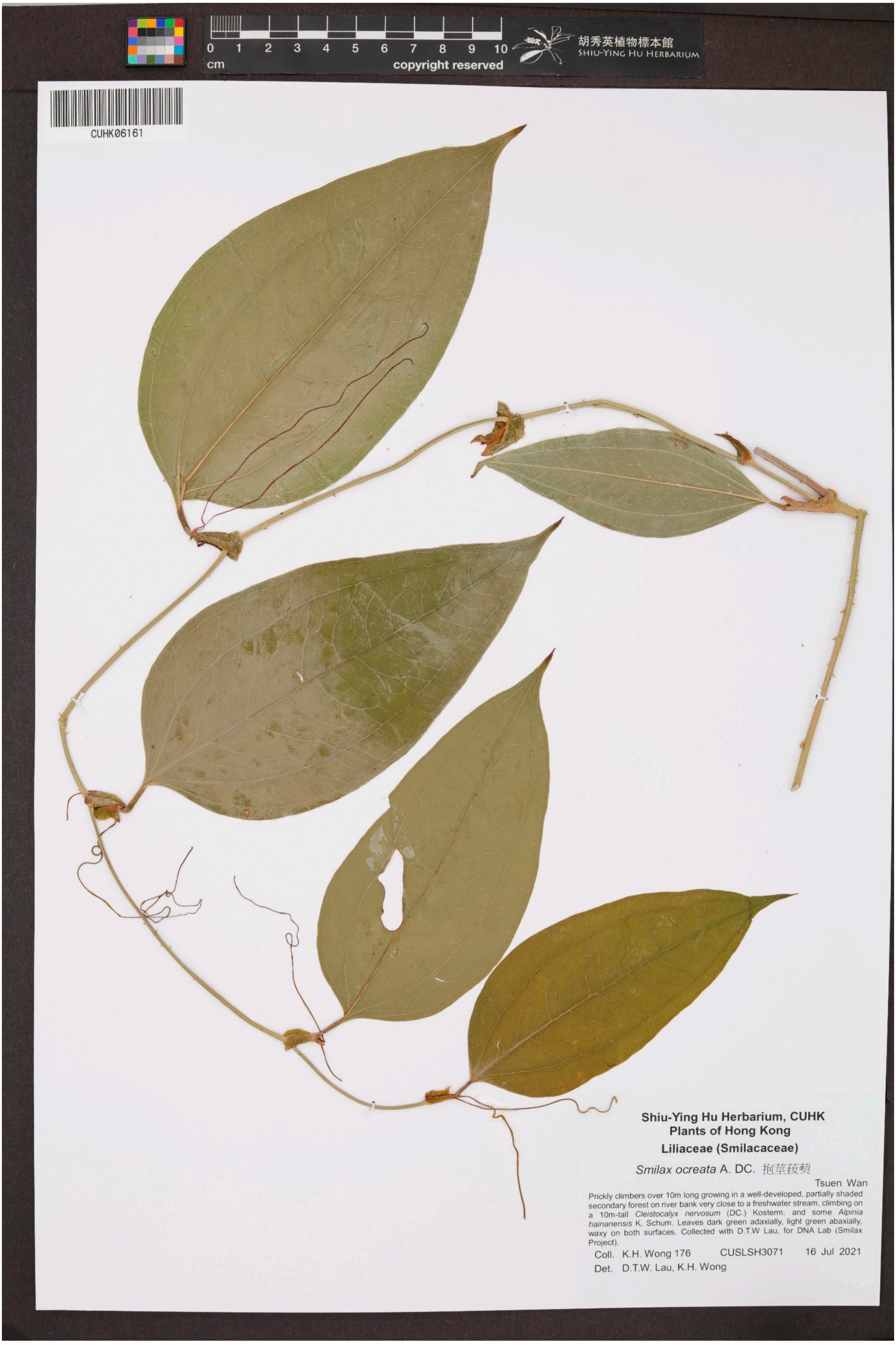

Figure S4s. Voucher specimen of the *Smilax ocreata* (K. H. Wong 176, CUHK06162)

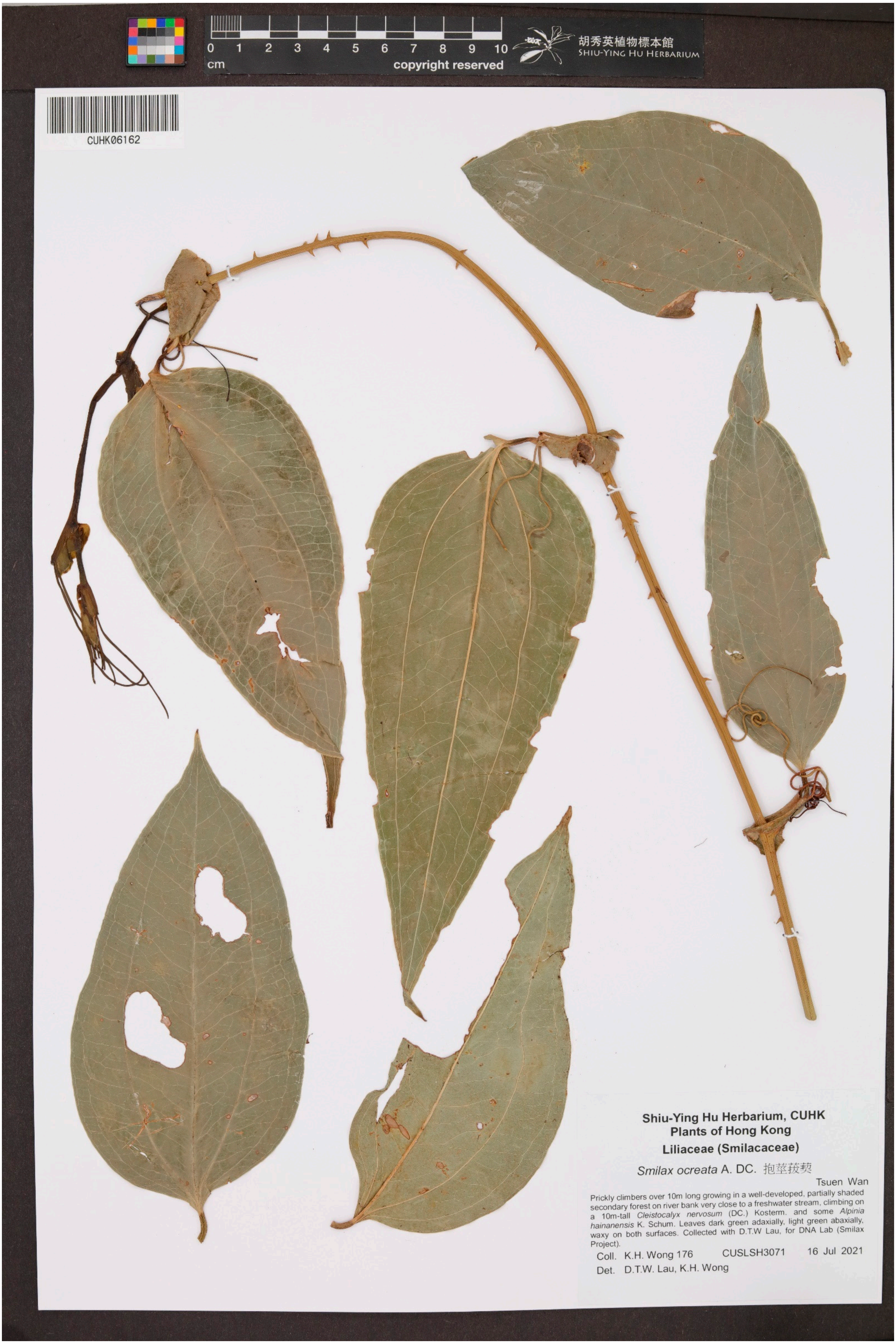

Figure S4t. Voucher specimen of the *Smilax ocreata* (K. H. Wong 176, CUHK06163)

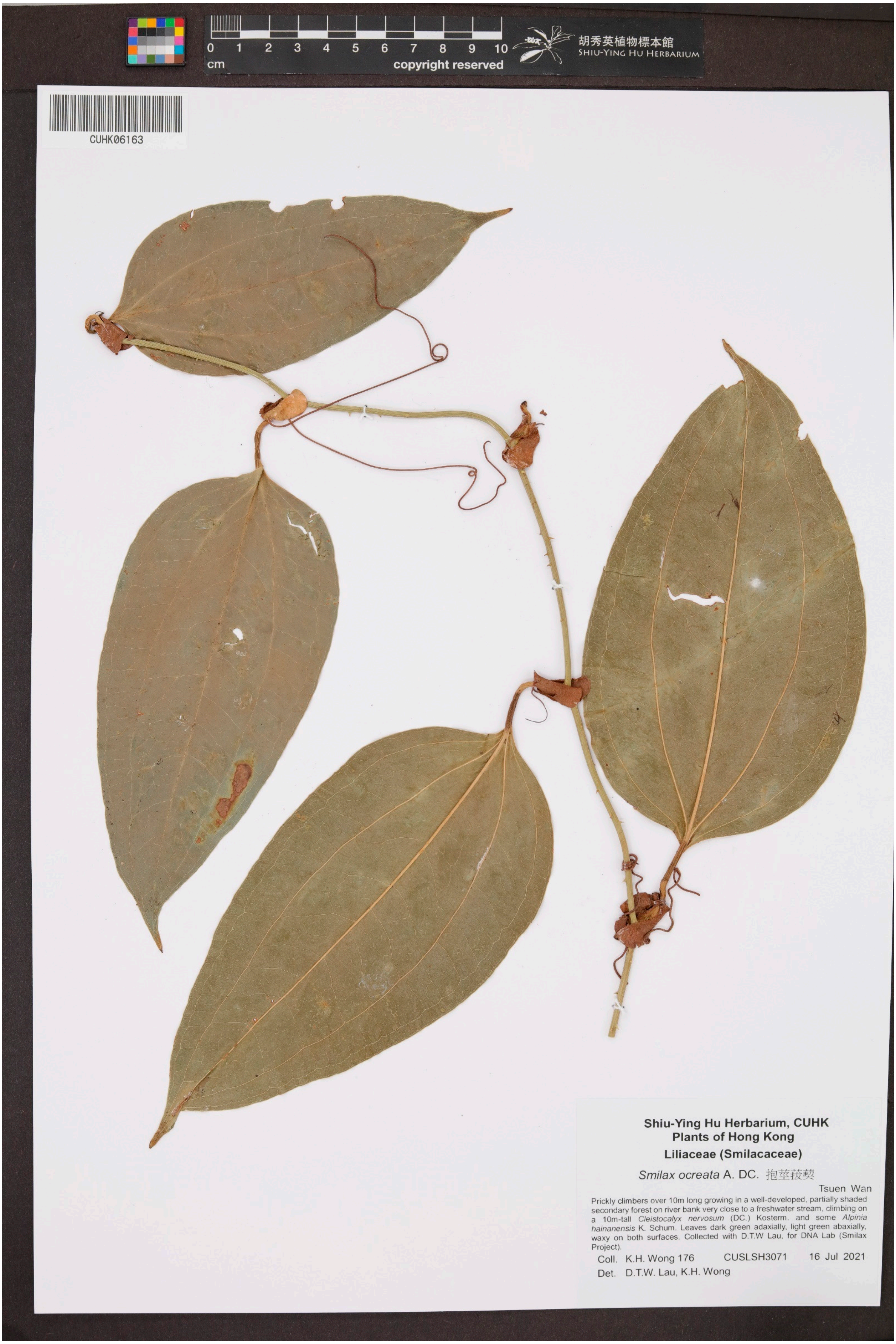

Figure S4u. Voucher specimen of the *Smilax ocreata* (K. H. Wong 176, CUHK06164)

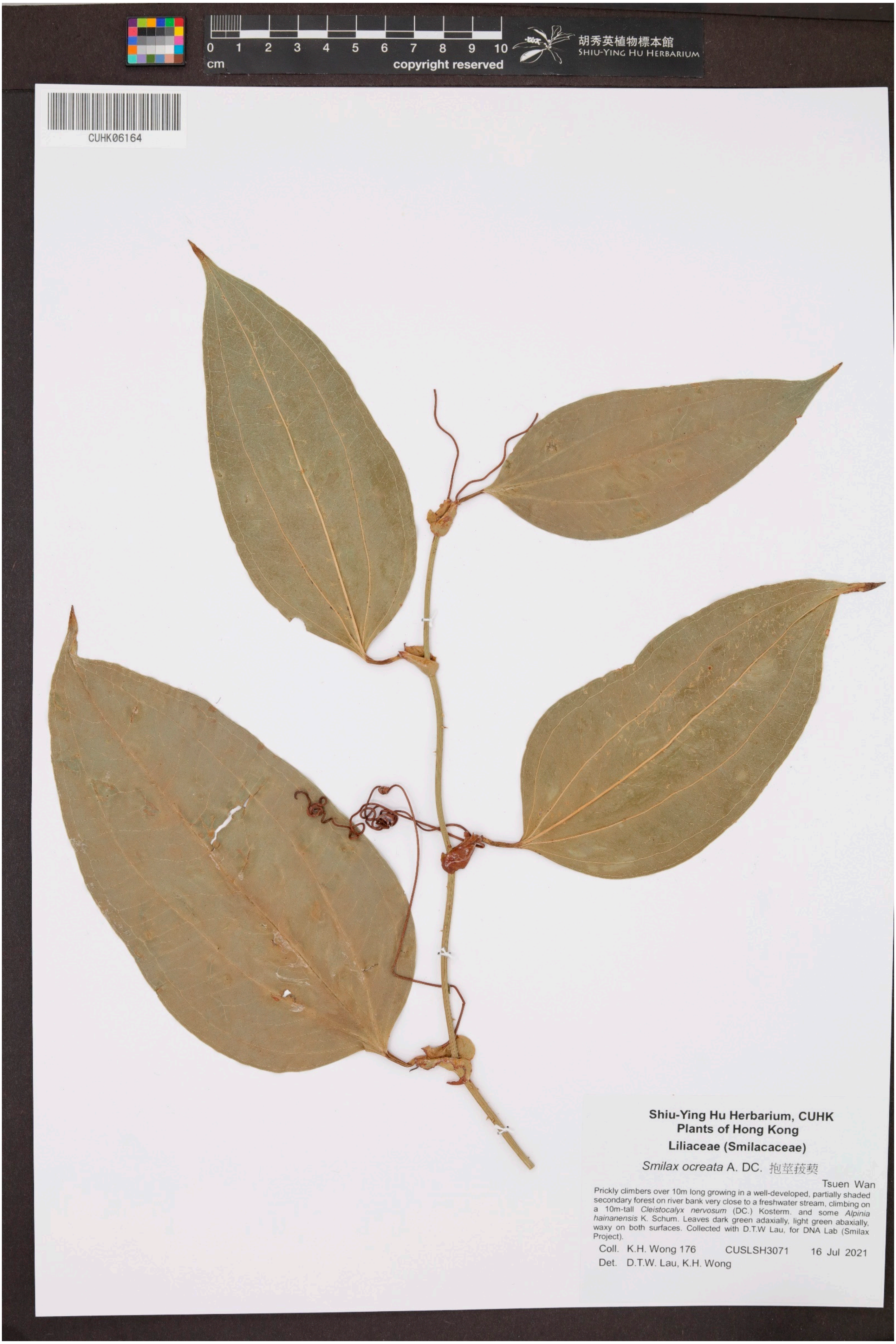

Figure S4v. Voucher specimen of the *Smilax ocreata* (K. H. Wong 176, CUHK06165)

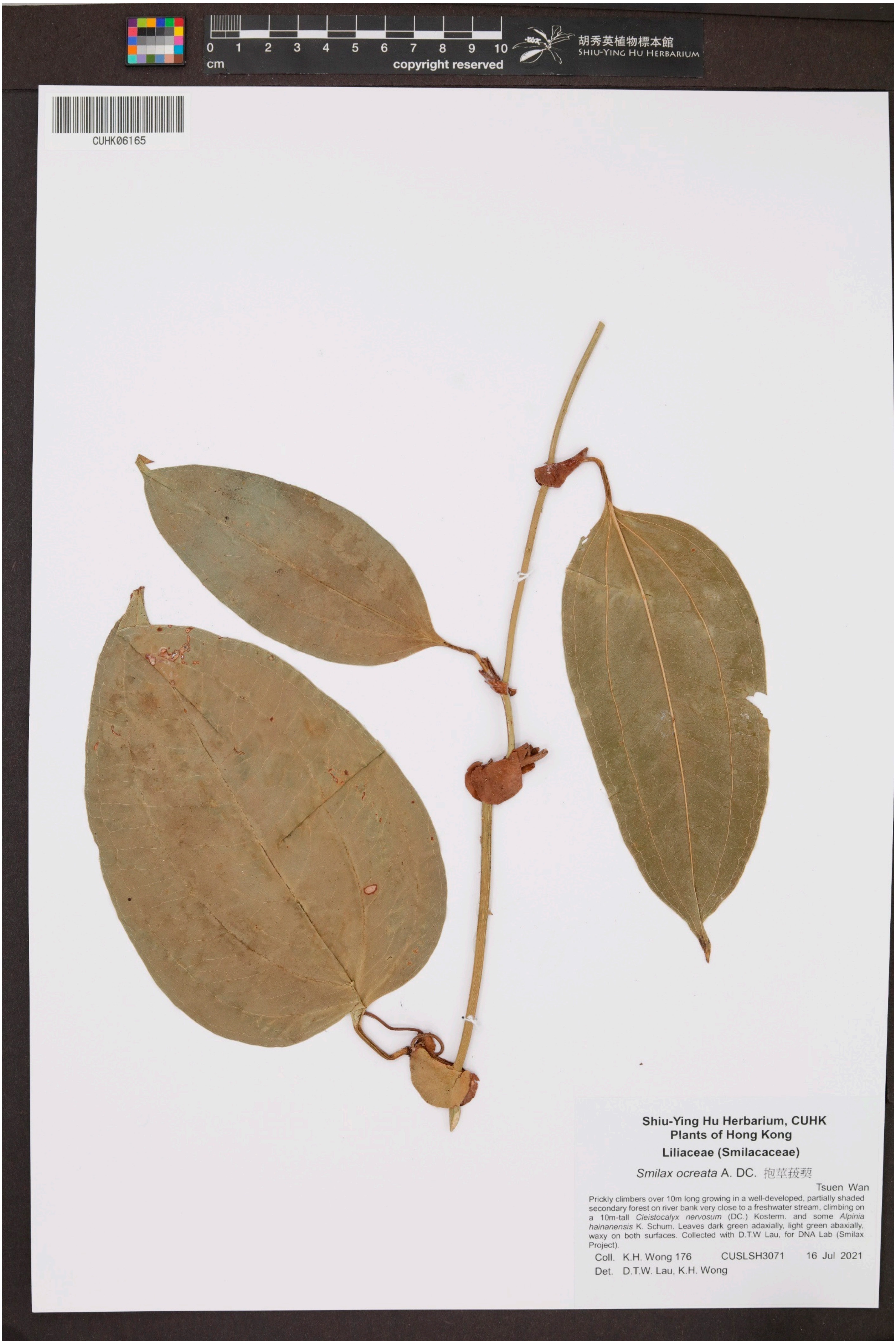

Figure S4w. Voucher specimen of the *Smilax ocreata* (K. H. Wong 176, CUHK06166)

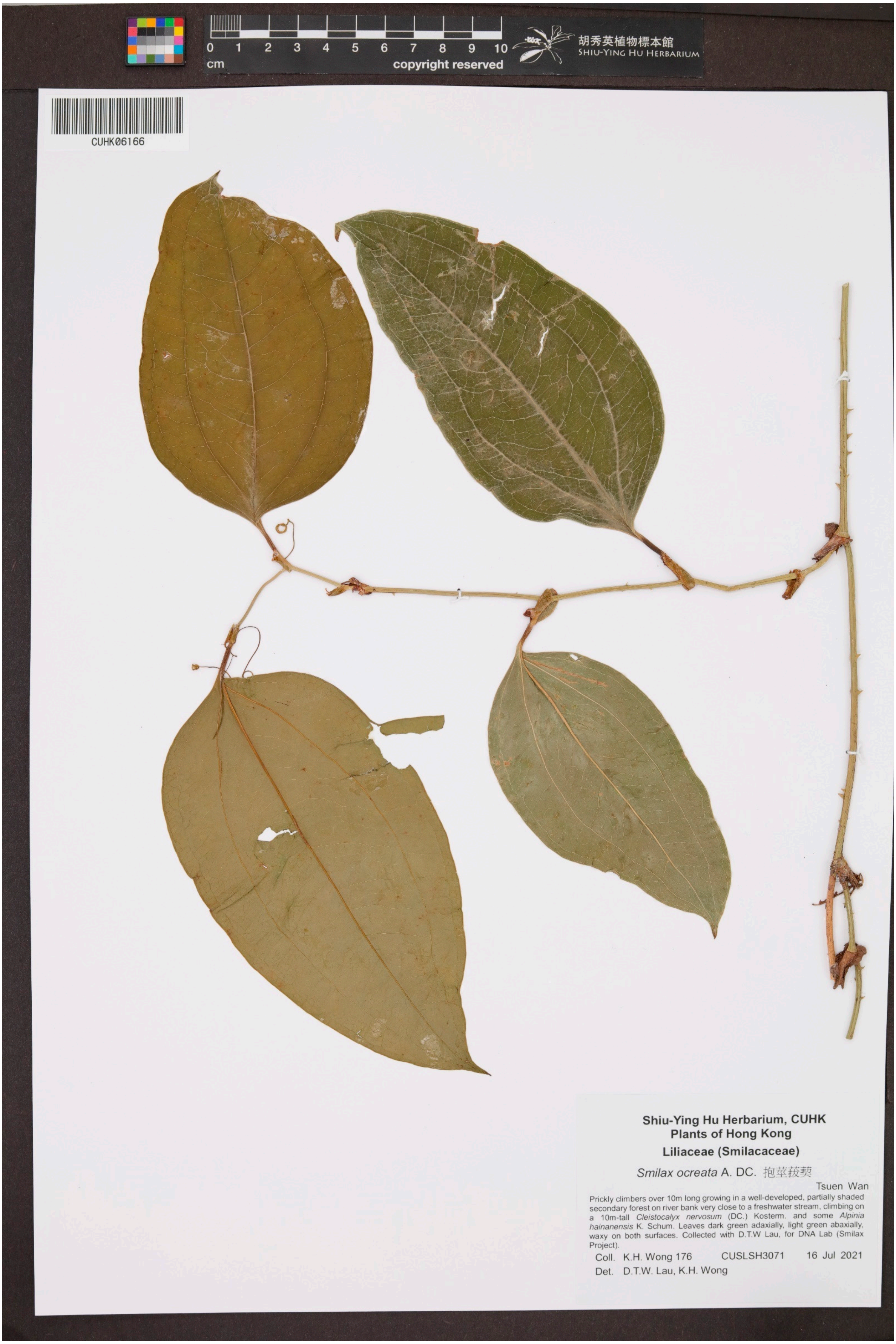

Figure S4x. Voucher specimen of the *Smilax ocreata* (K. H. Wong 176, CUHK06167)

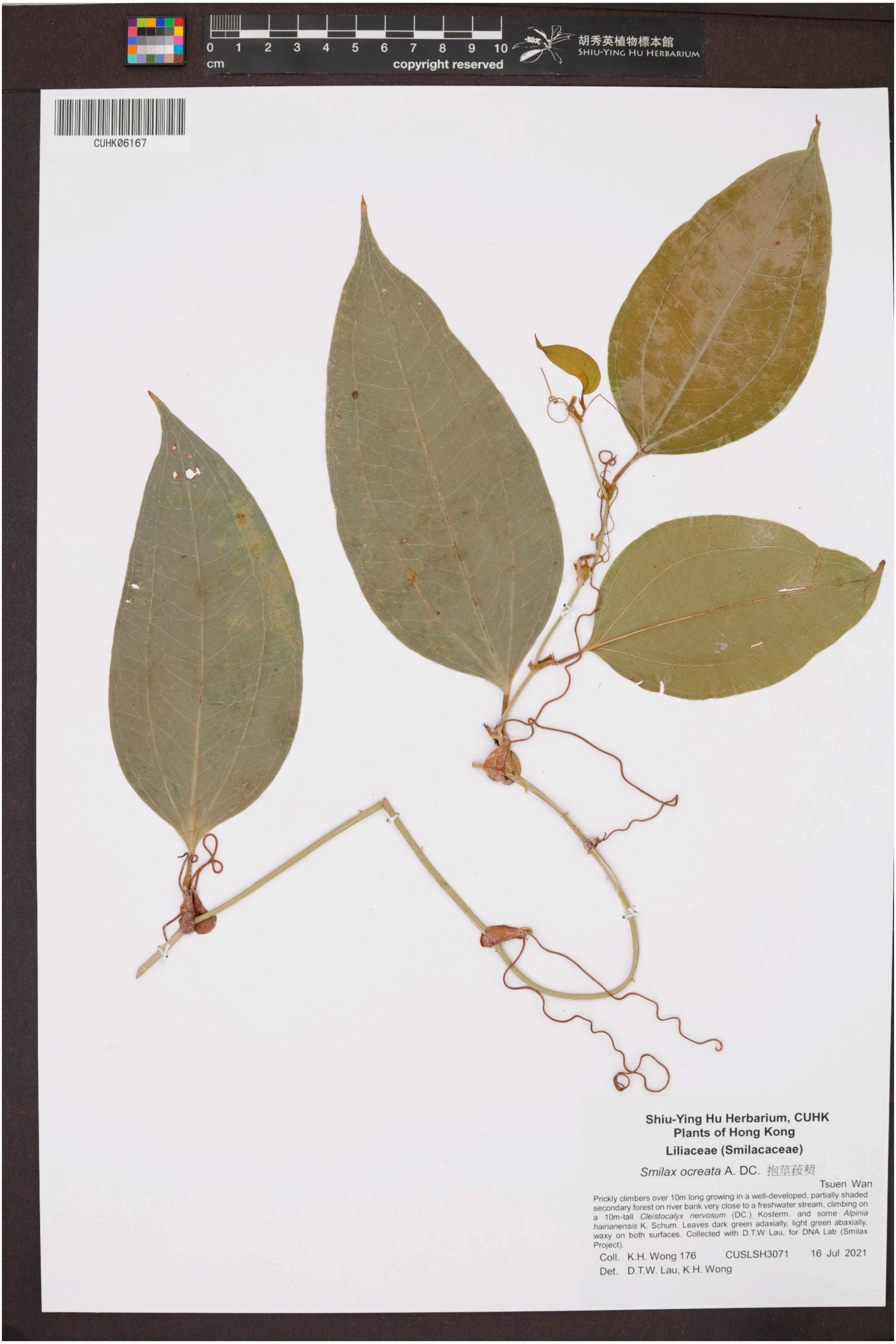

Figure S4y. Voucher specimen of the *Smilax ocreata* (K. H. Wong 176, CUHK06168)

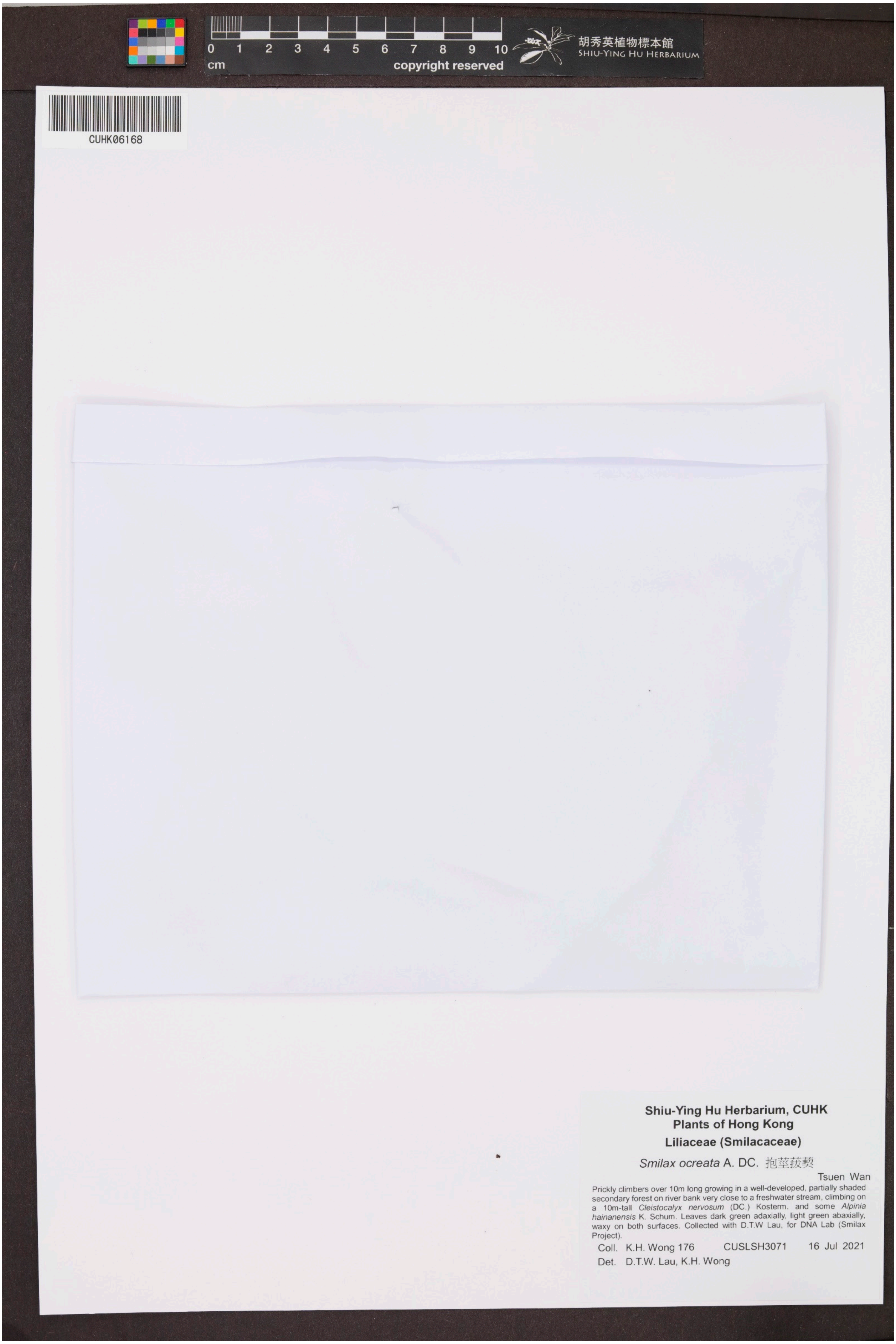

Figure S4z. Voucher specimen of the *Smilax ocreata* (K. H. Wong 176, CUHK06168)

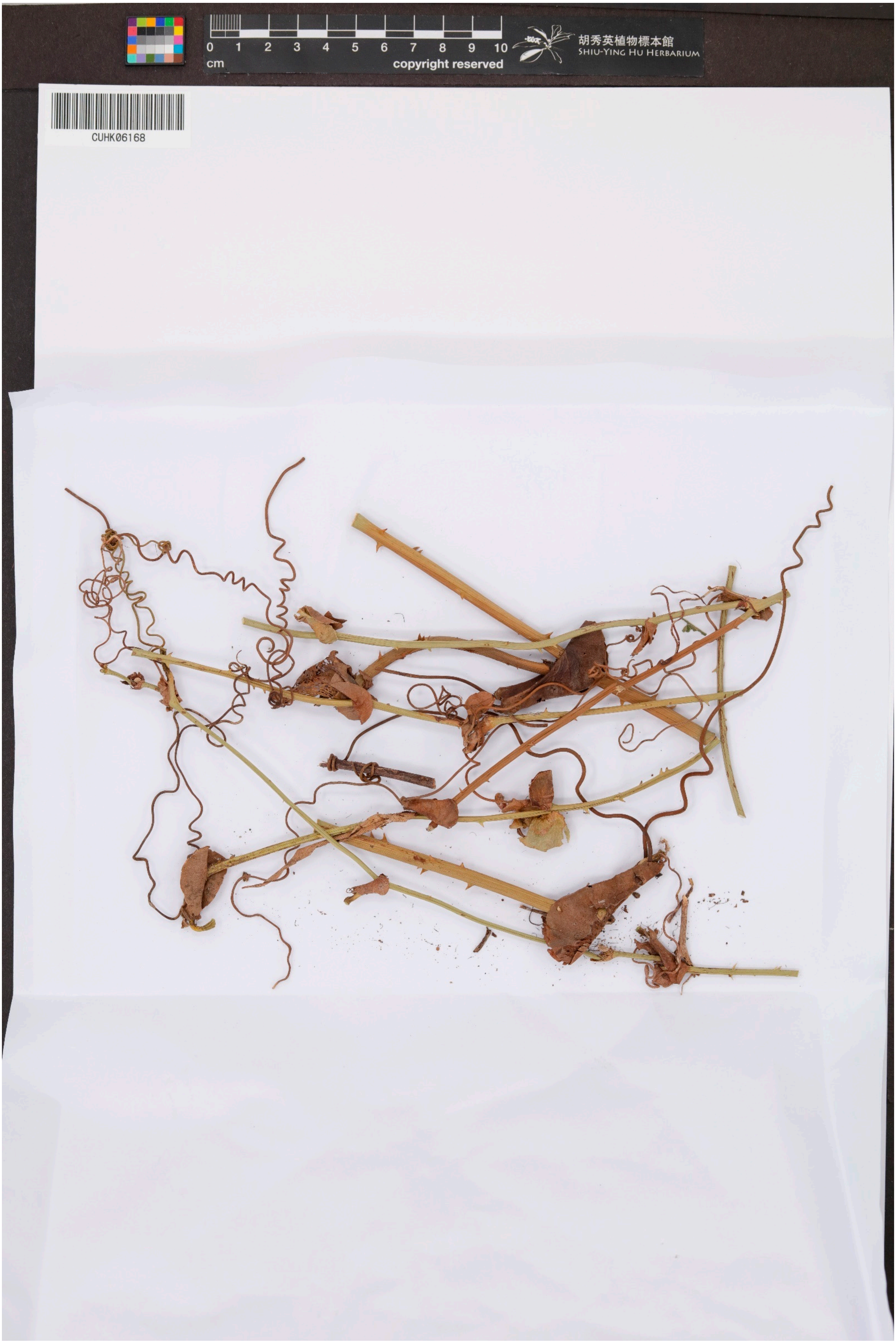

Figure S5a. Alignment visualisation of the complete chloroplast genomes of 14 *Smilax* species using *Smilax hypoglauca* as a reference (1 bp to 25,000 bp)

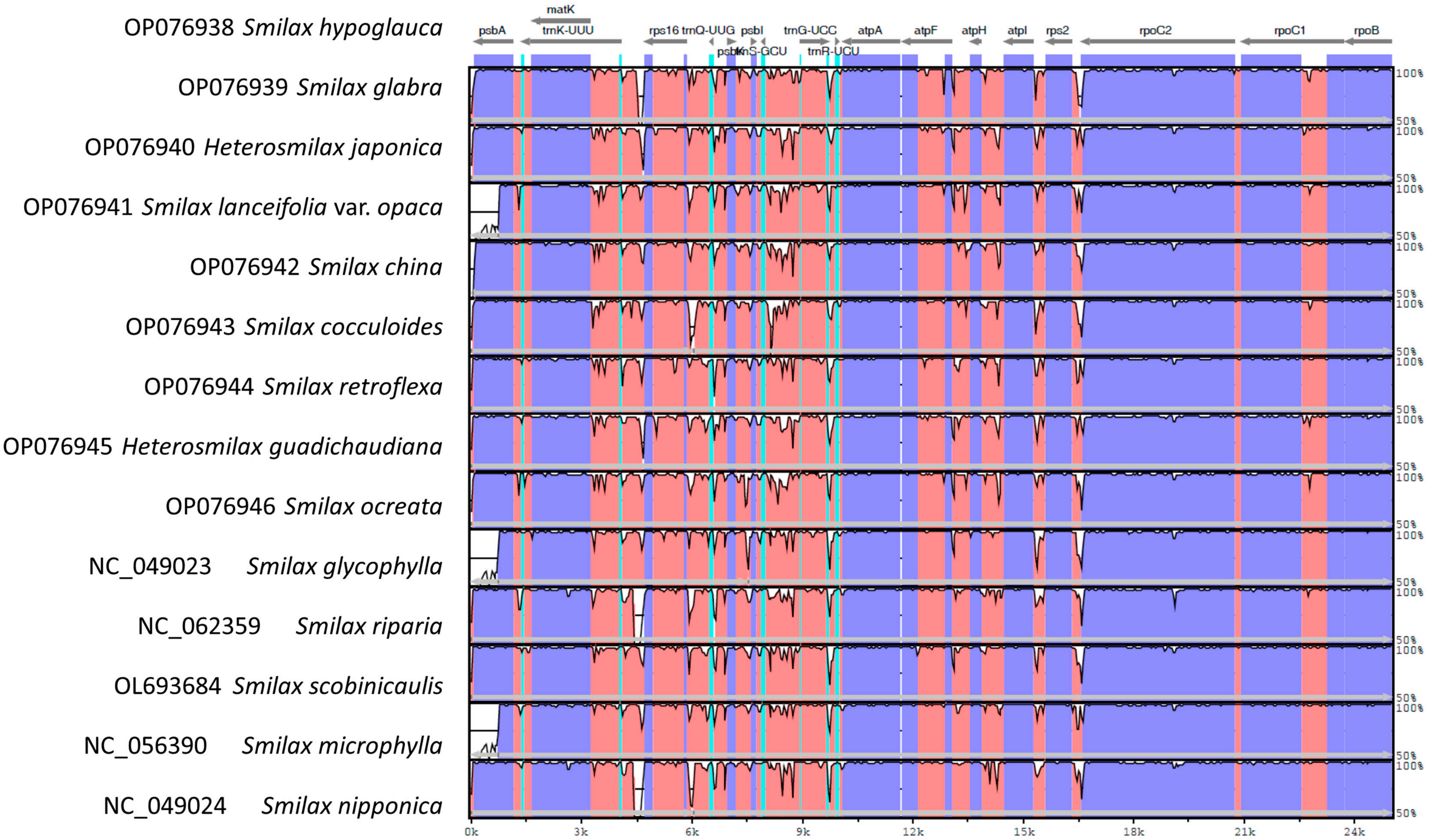

The vertical scale indicates the percentage of identity, ranging from 50 to 100%. The horizontal axis indicates the coordinates within the chloroplast genome. Grey arrows represent annotated genes; purple represent exon; light blue represent intron; pink represent untranslated region (UTR).

Figure S5b. Alignment visualisation of the complete chloroplast genomes of 14 *Smilax* species using *Smilax hypoglauca* as a reference (25,000 bp to 50,000 bp)

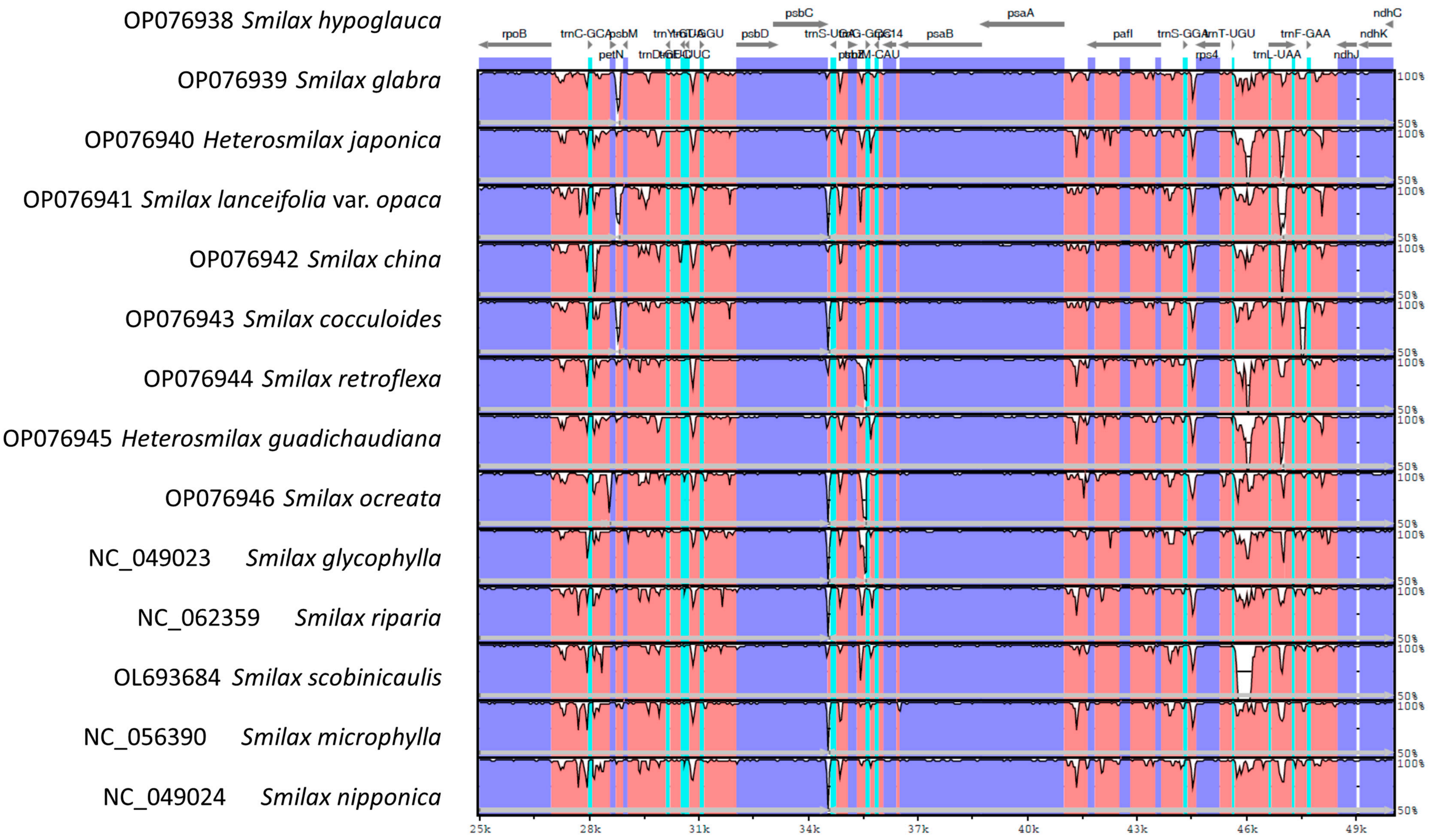

The vertical scale indicates the percentage of identity, ranging from 50 to 100%. The horizontal axis indicates the coordinates within the chloroplast genome. Grey arrows represent annotated genes; purple represent exon; light blue represent intron; pink represent untranslated region (UTR).

Figure S5c. Alignment visualisation of the complete chloroplast genomes of 14 *Smilax* species using *Smilax hypoglauca* as a reference (50,000 bp to 75,000 bp)

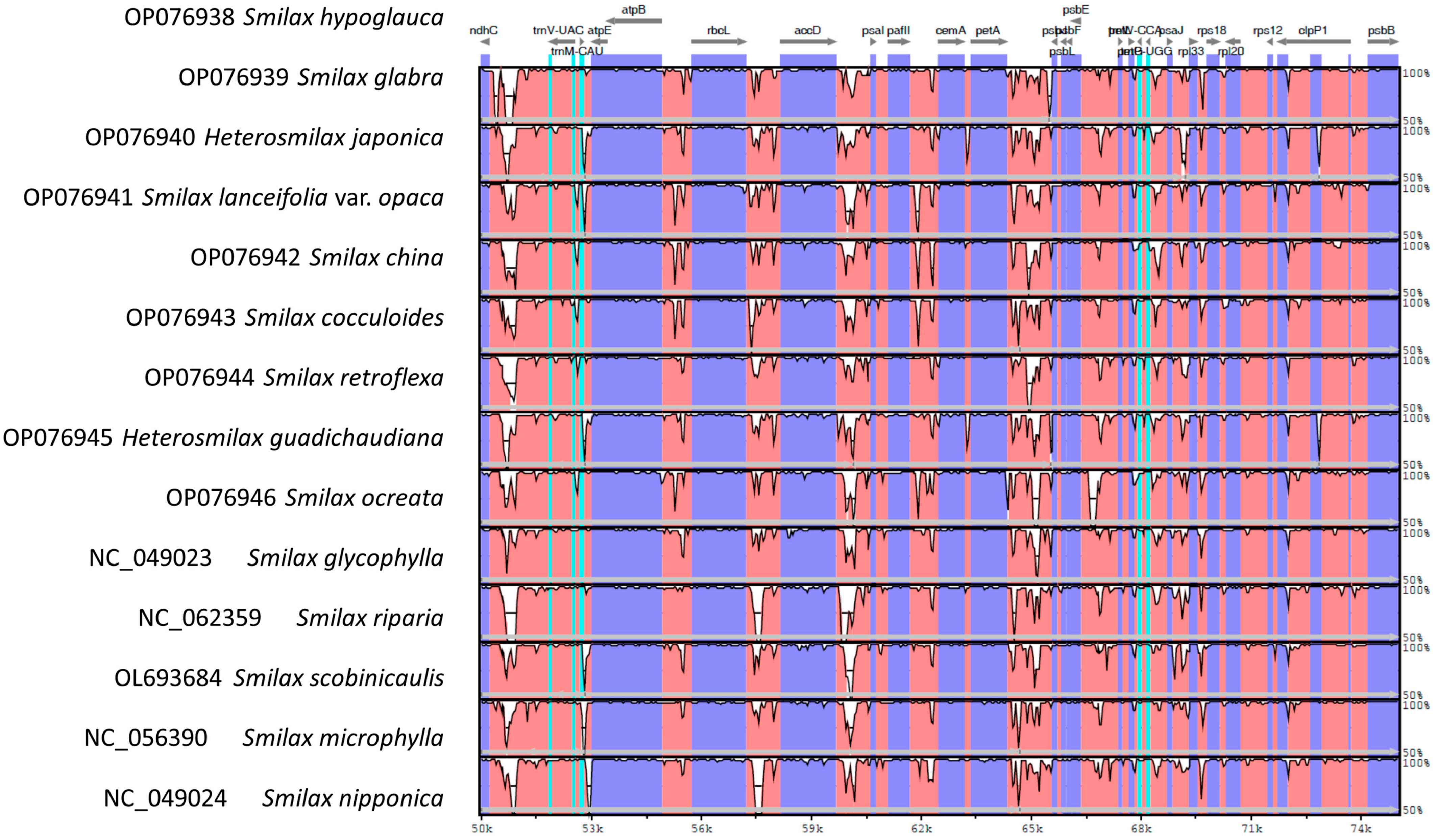

The vertical scale indicates the percentage of identity, ranging from 50 to 100%. The horizontal axis indicates the coordinates within the chloroplast genome. Grey arrows represent annotated genes; purple represent exon; light blue represent intron; pink represent untranslated region (UTR).

Figure S5d. Alignment visualisation of the complete chloroplast genomes of 14 *Smilax* species using *Smilax hypoglauca* as a reference (75,000 bp to 100,000 bp)

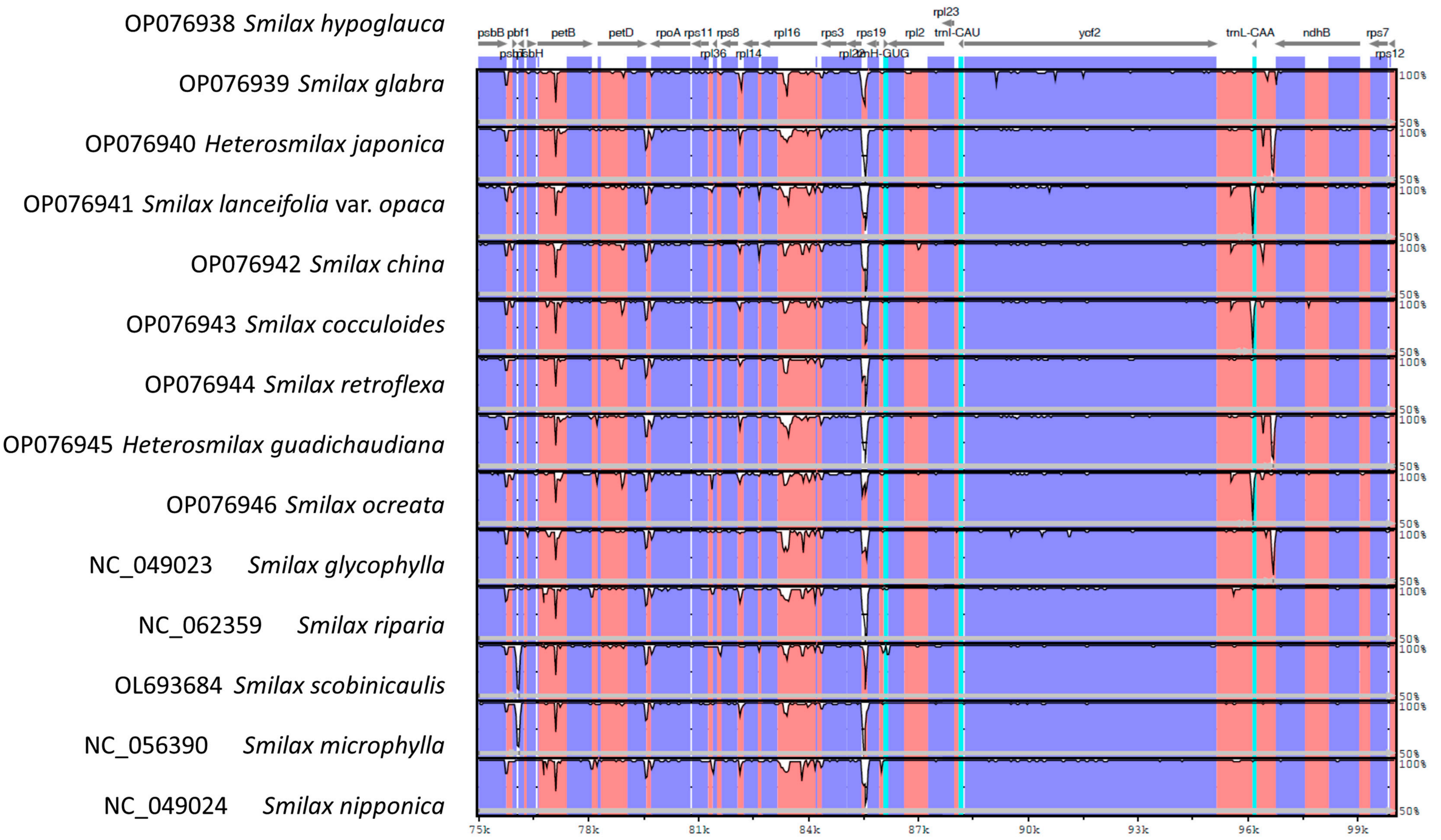

The vertical scale indicates the percentage of identity, ranging from 50 to 100%. The horizontal axis indicates the coordinates within the chloroplast genome. Grey arrows represent annotated genes; purple represent exon; light blue represent intron; pink represent untranslated region (UTR).

Figure S5e. Alignment visualisation of the complete chloroplast genomes of 14 *Smilax* species using *Smilax hypoglauca* as a reference (100,000 bp to 125,000 bp)

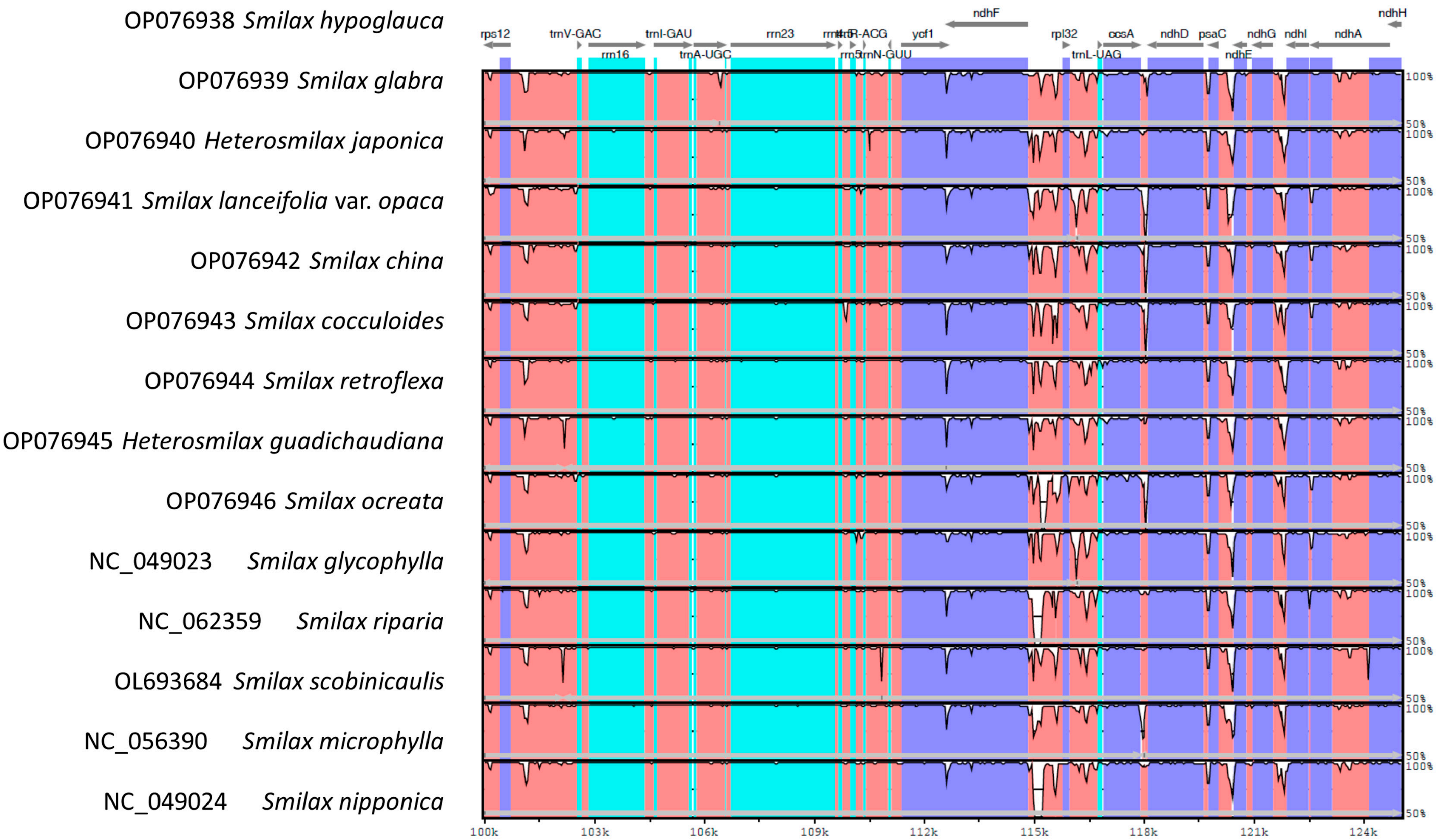

The vertical scale indicates the percentage of identity, ranging from 50 to 100%. The horizontal axis indicates the coordinates within the chloroplast genome. Grey arrows represent annotated genes; purple represent exon; light blue represent intron; pink represent untranslated region (UTR).

Figure S5f. Alignment visualisation of the complete chloroplast genomes of 14 *Smilax* species using *Smilax hypoglauca* as a reference (125,000 bp to 150,000 bp)

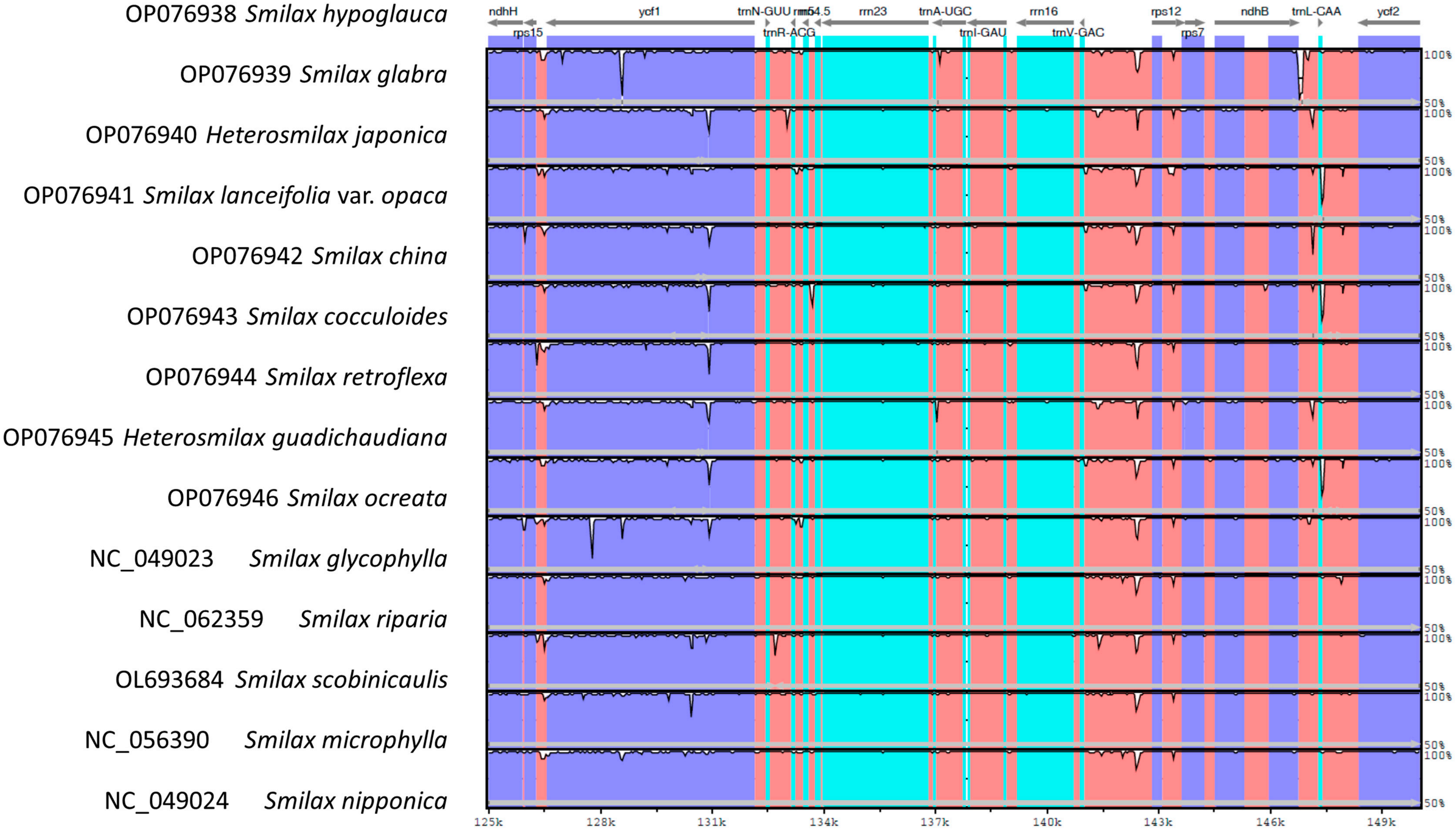

The vertical scale indicates the percentage of identity, ranging from 50 to 100%. The horizontal axis indicates the coordinates within the chloroplast genome. Grey arrows represent annotated genes; purple represent exon; light blue represent intron; pink represent untranslated region (UTR).

Figure S5g. Alignment visualisation of the complete chloroplast genomes of 14 *Smilax* species using *Smilax hypoglauca* as a reference (150,000 bp to 154,629 bp)

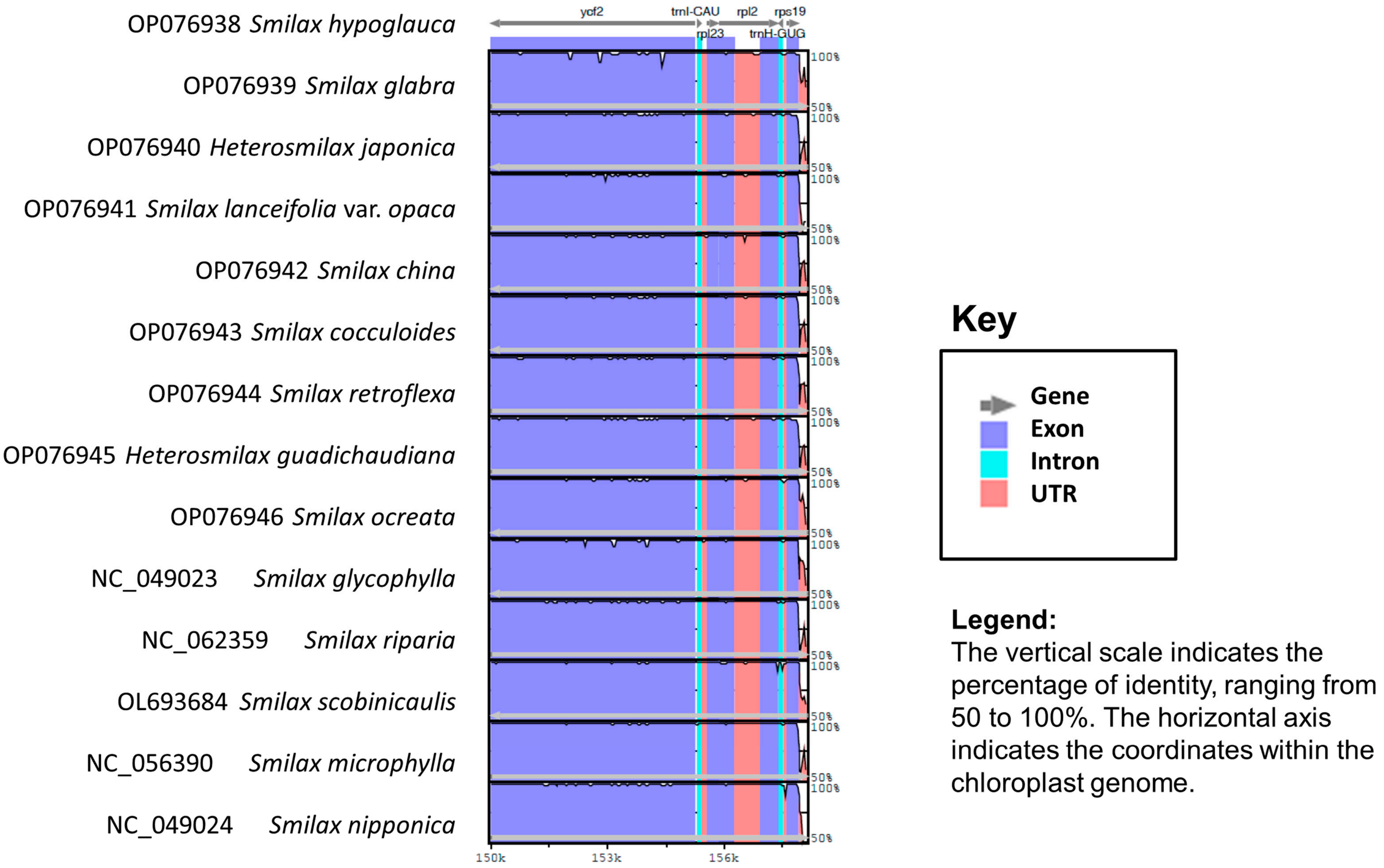

The vertical scale indicates the percentage of identity, ranging from 50 to 100%. The horizontal axis indicates the coordinates within the chloroplast genome. Grey arrows represent annotated genes; purple represent exon; light blue represent intron; pink represent untranslated region (UTR).
